# Exploring the Role and Pathophysiological Significance of Aldehyde Dehydrogenase 1B1 (ALDH1B1) in Human Lung Adenocarcinoma

**DOI:** 10.3390/ijms251910301

**Published:** 2024-09-25

**Authors:** Ilias Tsochantaridis, Dimitris Brisimis, Margaritis Tsifintaris, Anastasia Anastasiadou, Efthymios Lazos, Antreas Ermogenous, Sylia Christou, Nefeli Antonopoulou, Mihalis I. Panayiotidis, Michail I. Koukourakis, Alexandra Giatromanolaki, Aglaia Pappa

**Affiliations:** 1 Department of Molecular Biology & Genetics, School of Health Sciences, Democritus University of Thrace, 68100 Alexandroupolis, Greece; iliatsoc@gmail.com (I.T.); dimitrisbrisimis98@gmail.com (D.B.); mtsifintaris@gmail.com (M.T.); anastasiaanastasiadou5@gmail.com (A.A.); efthimioslazos@hotmail.com (E.L.); antreas_ermogenous@hotmail.com (A.E.); sylia.i.christou@gmail.com (S.C.); nefeliant@gmail.com (N.A.); 2 Department of Cancer Genetics, Therapeutics & Ultrastructural Pathology, The Cyprus Institute of Neurology & Genetics, Nicosia 2371, Cyprus; mihalisp@cing.ac.cy; 3 Radiotherapy/Oncology, Radiobiology & Radiopathology Unit, Department of Medicine, School of Health Sciences, Democritus University of Thrace, 68100 Alexandroupolis, Greece; targ@her.forthnet.gr; 4 Department of Pathology, University General Hospital of Alexandroupolis, Democritus University of Thrace, 68100 Alexandroupolis, Greece; agiatrom@med.duth.gr

**Keywords:** aldehyde dehydrogenases (ALDHs), ALDH1B1, A549, cell proliferation, cell morphology, chemoresistance, EMT, cancer stem cells (CSCs), CSC-related pathways, migration, lung adenocarcinoma

## Abstract

Aldehyde dehydrogenases (ALDHs) constitute a diverse superfamily of NAD(P)^+^-dependent enzymes pivotal in oxidizing endogenous and exogenous aldehydes to carboxylic acids. Beyond metabolic roles, ALDHs participate in essential biological processes, including differentiation, embryogenesis and the DNA damage response, while also serving as markers for cancer stem cells (CSCs). Aldehyde dehydrogenase 1B1 (ALDH1B1) is a mitochondrial enzyme involved in the detoxification of lipid peroxidation by-products and metabolism of various aldehyde substrates. This study examines the potential role of ALDH1B1 in human lung adenocarcinoma and its association with the CSC phenotype. To this end, we utilized the lung adenocarcinoma cell line A549, engineered to stably express the human ALDH1B1 protein tagged with green fluorescent protein (GFP). Overexpression of ALDH1B1 led to notable changes in cell morphology, proliferation rate and clonogenic efficiency. Furthermore, ALDH1B1-overexpressing A549 cells exhibited enhanced resistance to the chemotherapeutic agents etoposide and cisplatin. Additionally, ALDH1B1 overexpression correlated with increased migratory potential and epithelial–mesenchymal transition (EMT), mediated by the upregulation of transcription factors such as *SNAI2*, *ZEB2* and *TWIST1*, alongside the downregulation of *E-cadherin*. Moreover, Spearman’s rank correlation coefficient analysis using data from 507 publicly available lung adenocarcinoma clinical samples revealed a significant correlation between *ALDH1B1* and various molecules implicated in CSC-related signaling pathways, including Wnt, Notch, hypoxia, Hedgehog, retinoic acid, Hippo, NF-κΒ, TGF-β, PI3K/PTEN-AKT and glycolysis/gluconeogenesis. These findings provide insights into the role of ALDH1B1 in lung tumor progression and its relation to the lung CSC phenotype, thereby offering potential therapeutic targets in the clinical management of lung adenocarcinoma.

## 1. Introduction

Aldehyde dehydrogenases (ALDHs) constitute a superfamily of NAD(P)^+^-dependent enzymes responsible for oxidizing endogenous and exogenous aldehydes to their respective carboxylic acids. Found across diverse species, including primates, rodents, birds, fish and zebrafish, these enzymes exhibit specific chromosomal locations. In the human genome, 19 ALDH genes are categorized into 11 families and four subfamilies encoding various isoforms with distinct cellular localizations (cytoplasm, mitochondria, endoplasmic reticulum, nucleus), tissue distributions, substrate specificities and expression patterns [1]. Beyond their role in metabolizing aldehydes as part of the body’s antioxidative defense mechanisms, specific ALDH isoforms are involved in the synthesis of retinoic acid (RA), betaine and γ-aminobutyric acid (GABA). These enzymes have also been identified as markers of cancer stem cells (CSCs) [2] due to their involvement in aldehyde detoxification, protection against oxidative stress, stemness maintenance, metabolic adaptation and other biological processes such as differentiation, embryogenesis and DNA damage response [1,3]. ALDH bright (ALDH^br^) cells exhibit stem-like characteristics such as self-renewal, clonogenic growth, tumor-initiating ability and chemoresistance in different malignancies [2]. Plenty of clinical studies indicated that tumor tissues that survive after the chemotherapeutic approach contain an increased percentage of CSCs and, thus, ALDH^br^ cells compared to original tumor tissues [2].

Aldehyde dehydrogenase 1B1 (ALDH1B1) is a mitochondrial enzyme consisting of 517 amino acids, which is involved in ethanol metabolism. It primarily detoxifies lipid peroxidation by-products, such as malondialdehyde (MDA) and 4-hydroxy-nonenal (4-HNE). It also metabolizes nitroglycerin and all-trans retinaldehyde [4]. Recent studies highlight its involvement in pancreatic stem cells, beta cell development and various CSC-related signaling pathways, including PI3K/Akt, Wnt/β-catenin and Notch, underscoring its potential association with the CSC phenotype [5].

Lung cancer ranks as the second most common type of cancer and the leading cause of cancer-related deaths in the USA [6]. ALDH1B1 has been identified as a biomarker for colorectal cancer [1,7] and has been linked to cancer stem-like features [3]. Recent studies have demonstrated its correlation with lung tumorigenesis [8,9]. ALDH1B1 transcriptional and translational expression levels were significantly increased in A549/DDP (cisplatin-resistant) cells compared to A549 cells, suggesting the association between ALDH1B1 and chemoresistance [8]. Moreover, the relative expression levels of ALDH1B1 were higher in lung adenocarcinoma TCGA samples compared to normal tissue [9,10].

In light of the association between ALDH1B1-mediated lung tumor formation, chemoresistance and CSC phenotype, our study focused on elucidating the contribution of ALDH1B1 to the pathophysiological mechanisms underlying human lung adenocarcinoma. For this purpose, we employed a gene overexpression strategy utilizing the human lung adenocarcinoma (A549) cell line and data analysis of lung adenocarcinoma clinical samples [11,12]. This enabled us to examine the impact of ALDH1B1 overexpression on various aspects, including (i) cell parameters of morphology, proliferation and colony formation efficiency, (ii) chemoresistance, (iii) gene expression profile of EMT-related markers and CSC signaling pathway-associated molecules and (iv) cell migration. Our research contributes to our understanding of the role of ALDH1B1 in malignancies, highlighting its potential as an important biomarker in human lung adenocarcinoma.

## 2. Results

### 2.1. Generation and Characterization of A549 Isogenic Cell Line Pair

**Figure 1:**
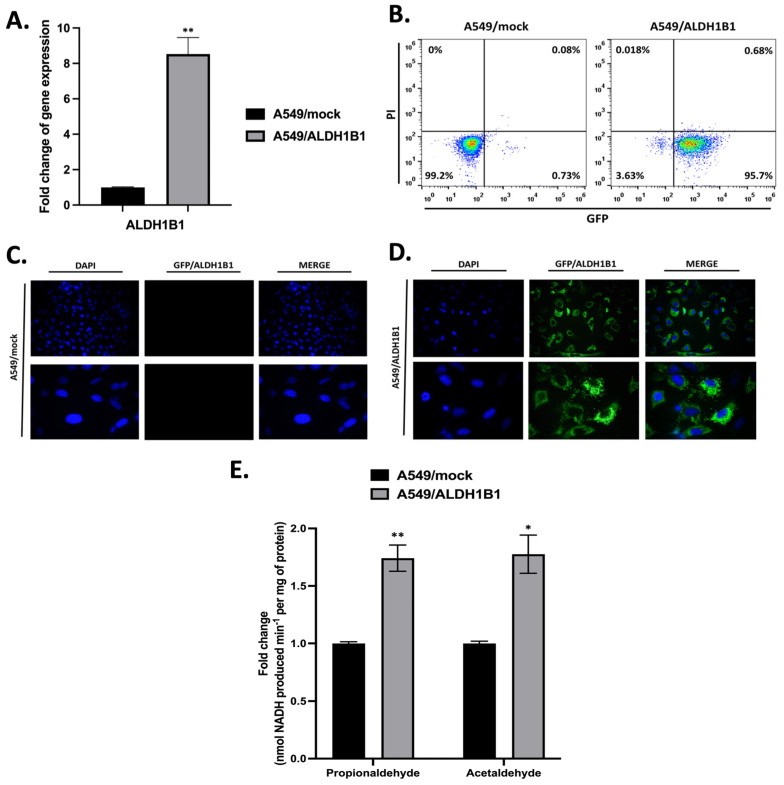
**Overexpression of ALDH1B1 in A549 cells.** (**A**) ALDH1B1 gene expression levels were measured by real-time PCR in A549/mock and A549/ALDH1B1 cells. (**B**) GFP-positive cells (green) were assessed in A549/mock and A549/ALDH1B1 cells by using flow cytometry. Nuclei were counterstained with propidium iodide (PI) (red). Fluorescence microscopy was used to visualize GFP-positive cells (green) in A549/mock (**C**) and A549/ALDH1B1 (**D**) cells with images captured at 40× magnification (upper panel) and 100× magnification (lower panel). Nuclei were stained with DAPI (4′,6-diamino-2-phenylindole) (blue). (**E**) ALDH1B1 enzymatic activity was assessed in A549/mock and A549/ALDH1B1 cells using propionaldehyde or acetaldehyde as substrates. Results are represented as mean ± S.D. of three independent experiments. * *p* < 0.05, ** *p* < 0.01.

Stable transfection of human ALDH1B1 cDNA into A549 cells led to the generation of the A549/ALDH1B1 polyclonal cell line (Figure 1). ALDH1B1 expression was assessed by real-time PCR, revealing 8-fold higher transcriptional levels of ALDH1B1 in A549/ALDH1B1 cells compared to control (A549/mock) cells. (Figure 1A). The transfection efficiency was calculated by monitoring the expression of GFP-tagged ALDH1B1 by flow cytometry. In A549/ALDH1B1 cells, 95.7% were GFP+ (0.7% were PI+), while, in A549/mock, 99.2% were GFP- (0.08% were PI+) (Figure 1B). Regular observation of ALDH1B1 mRNA levels, along with GFP tag fluorescence, confirmed the maintenance of stable ALDH1B1 expression. The increased GFP fluorescence in A549/ALDH1B1 compared to A549/mock cells was also validated by fluorescence microscopy (Figure 1C,D). ALDH1B1 enzymatic activity levels were measured using the aldehyde substrates, propionaldehyde and acetaldehyde, commonly used to determine the activity of ALDH1/2 isozymes [13]. ALDH1B1 activity levels were approximately 1.8-fold higher in A549/ALDH1B1 compared to A549/mock cells using both aldehyde substrates (Figure 1E, Table 1).

### 2.2. ALDH1B1 Is Associated with Altered Cell Morphology as Well as Decreased Cell Proliferation and Clonogenicity in A549 Cells

**Figure 2:**
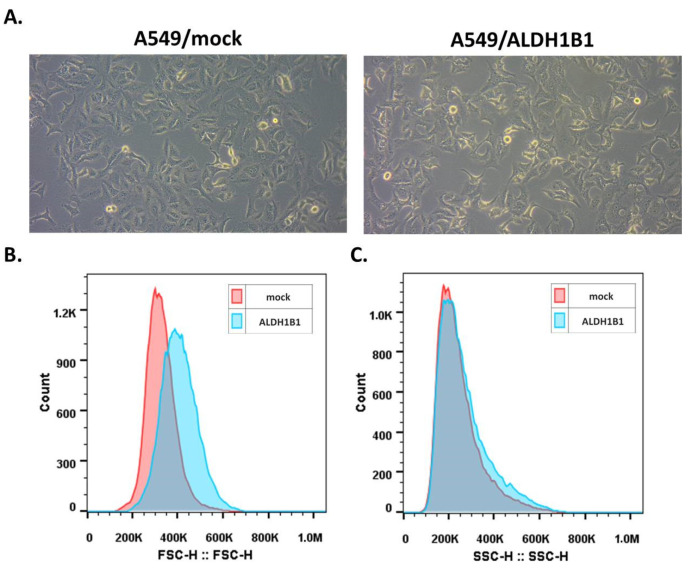
Expression of ALDH1B1 induces significant morphological changes in A549 cells. (**A**) Optical microscopy images (20× magnification) of the isogenic A549 cell line pair illustrating notable morphological differences. ALDH1B1-overexpressing cells display variations in (**B**) size and (**C**) granularity compared to control (mock) cells. Flow cytometry analysis of at least 30,000 events was performed to determine the median fluorescence intensities for forward scatter (FCS) and side scatter (SSC) parameters (see Table 2). Representative histograms of FSC (**B**) and SSC (**C**) of A549/mock and A549/ALDH1B1 cells are shown. The FCS histogram of ALDH1B1-overexpressing cells (light blue) is markedly shifted to the right, indicating a larger cell size relative to the A549/mock cells (pink). Graphs represent the findings of three independent experiments.

We observed that overexpressing ALDH1B1 caused notable morphological changes in A549 cells (Figure 2A). Specifically, cells with elevated ALDH1B1 expression levels exhibited a more elongated shape compared to control (mock) cells. To assess cell morphology, we employed a standard flow cytometry using forward scatter (FSC) and side scatter (SSC) parameters for analysis [3]. The resulting histograms for FSC (Figure 2B) and SSC (Figure 2C) revealed an increase in cell size in A549/ALDH1B1 cells compared to A549/mock cells. The median fluorescence intensity values for FSC and SSC are also shown in Table 2.

**Figure 3:**
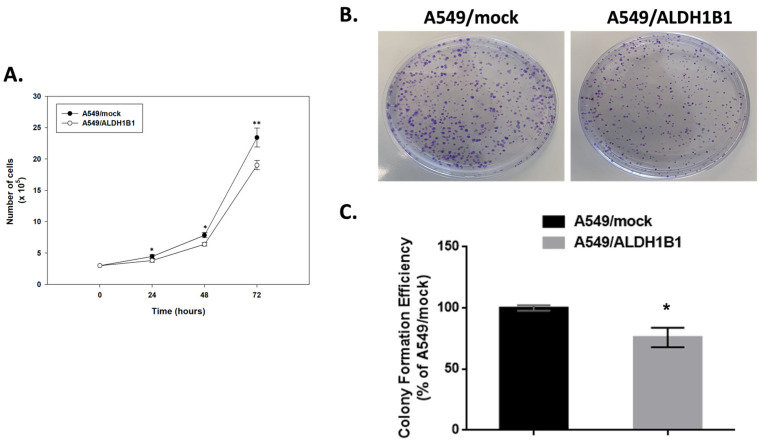
ALDH1B1 overexpression reduces cell proliferation rate and clonogenic efficiency in A549 cells. (**A**) Growth rates of A549/mock and A549/ALDH1B1 cells. Cells were plated at a density of 3 × 10^5^ cells/plate in 10 cm culture plates and the total number of cells was recorded every 24 h over a 3-day period. (**B**) Representative images showing colony formation efficiency of A549/mock and A549/ALDH1B1 cells. Cells (10^3^) were seeded in 10 cm culture plates, and colony formation was evaluated approximately 20 days after seeding. Colonies were fixed, stained with crystal violet, and counted. (**C**) Quantitative assessment of colony formation efficiency. Results are presented as mean ± SD of three independent experiments. * *p* < 0.05.

Next, we investigated how ALDH1B1 affects the proliferation rate of A549 cells. Notably, A549/ALDH1B1 cells demonstrated reduced growth rates compared to A549/mock cells (Figure 3A). Cells were seeded and counted at four time points (0, 24, 48 and 72 h). The cell count was significantly lower in A549/ALDH1B1 cells than in control (mock) cells (Figure 3A). In particular, the doubling time (tD) of A549/ALDH1B1 cells was prolonged, while their growth rates (GR) were lower compared to the corresponding values in A549/mock cells (Table 3). Furthermore, the number of colonies counted in ALDH1B1-overexpressing cells was significantly reduced compared to control (mock) cells, implying a potential effect of ALDH1B1 on A549 cell clonogenicity (Figure 3B). Finally, the estimated colony formation efficiency of A549/ALDH1B1 cells was approximately 20% lower than that of the control (mock) cells (Figure 3C).

### 2.3. ALDH1B1 Overexpression Is Associated with Enhanced Chmeresistance in A549 Cells

**Figure 4:**
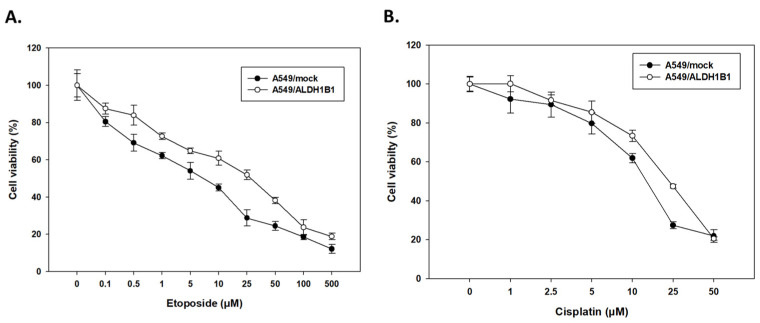
Concentration–response curves of (**A**) etoposide and (**B**) cisplatin in A549/mock and A549/ALDH1B1 cells. All cells were treated with increasing concentrations of the chemotherapeutic agents for 48 h. The concentration–response curves of the A549/ALDH1B1 cells were shifted to the right, indicating increased cellular tolerance to the cytotoxic effect of the chemotherapeutic agents.

Next, we examined the association of ALDH1B1 with chemoresistance. To this end, A549/mock and A549/ALDH1B1 cells were exposed to increasing concentrations of etoposide (0–500 μΜ) or cisplatin (0–50 μΜ) for 48 h. For each chemotherapeutic agent, a concentration–response curve was plotted (Figure 4A,B) and the corresponding IC_50_ values were determined (Table 4). Our findings indicated that ALDH1B1 correlates with a chemoresistant phenotype, as evidenced by the shifted concentration–response curves of A549/ALDH1B1 cells to the right compared to A549/mock cells (Figure 4A,B). ALDH1B1-overexpressing cells exhibited approximately three-fold resistance to etoposide (as shown in Figure 4A and Table 4) and approximately two-fold resistance to cisplatin (as depicted in Figure 4B and Table 4) when compared to control (mock) cells.

### 2.4. ALDH1B1 Enhances Migration and Induces EMT by Upregulating SNAI2, ZEB2 and TWIST1 in A549 Cells

**Figure 5:**
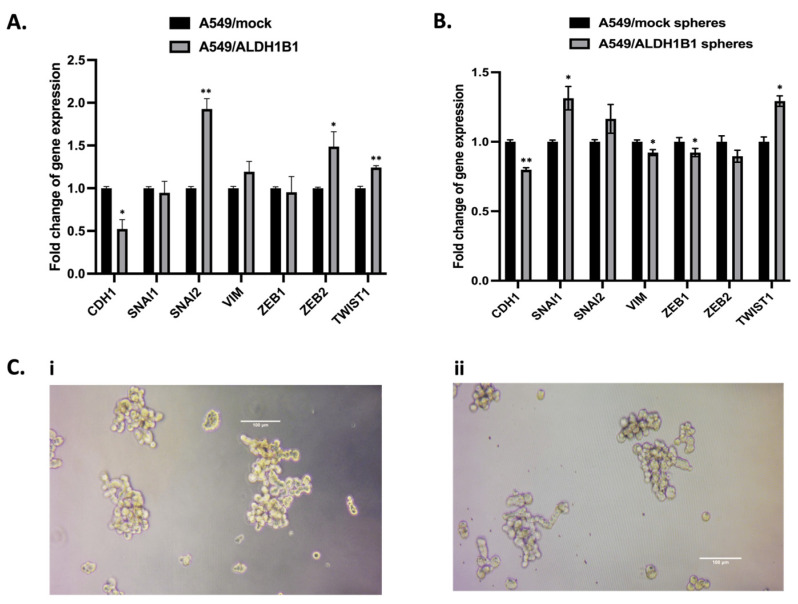
ALDH1B1 overexpression induces EMT in A549 cells and spheres. (**A**) Effects of ALDH1B1 on the gene expression levels of EMT-related transcription factors. (**B**) Effects of ALDH1B1 on the gene expression levels of EMT-related transcription factors in spheres (cancer stem-like cells) of A549/mock and A549/ALDH1B1 cells. Gene expression fold-changes were analyzed using the ΔΔCt method with *β-actin* as the endogenous control for normalization. (**C**) Representative images of (**i**) A549/mock and (**ii**) A549/ALDH1B1 spheres (magnification 20×), typically formed in approximately 10 days. Results are presented as mean ± SD from at least three independent experiments. * *p* < 0.05, ** *p* < 0.01.

Decreased cell proliferation and morphological alterations of ALDH1B1-overexpressing cells, combined with their resistance to chemotherapy, led us to investigate whether ALDH1B1 expression is associated with the EMT phenotype and affects cell migration in A549 cells. EMT typically involves slower cell proliferation and alterations in cytoskeleton structure [3]. Induction of EMT is often associated with cell stemness characteristics and increased metastatic potential [3]. In general, it is characterized by disruption of cellular junctions, loss of apical–basal polarity and morphological changes in the cytoskeleton, thus promoting cell invasiveness [3]. Therefore, we examined the transcriptional expression levels of various effector molecules and transcription factors involved in EMT. Specifically, we analyzed the mRNA expression levels of E-cadherin, SNAI1, SNAI2, vimentin, ZEB1, ZEB2, TWIST1 and N-cadherin in A549/mock and A549/ALDH1B1 cells. Our findings revealed that ALDH1B1 upregulated the mRNA levels of SNAI2, ZEB2 and TWIST1, leading to down-regulation of E-cadherin. (Figure 5A). Since EMT is relevant to both normal as well as cancer stem epithelial cells and contributes to the formation of metastatic cancer stem cells, we also evaluated the expression of EMT-related genes in A549-generated spheres (Figure 5C). Our results showed that ALDH1B1 promotes EMT by increasing the levels of SNAI1/2, ZEB2 and TWIST1, consequently reducing the expression of CDH1 (E-cadherin) followed by a slight decrease in vimentin and ZEB1 levels in A549-generated spheres (Figure 5A).

**Figure 6:**
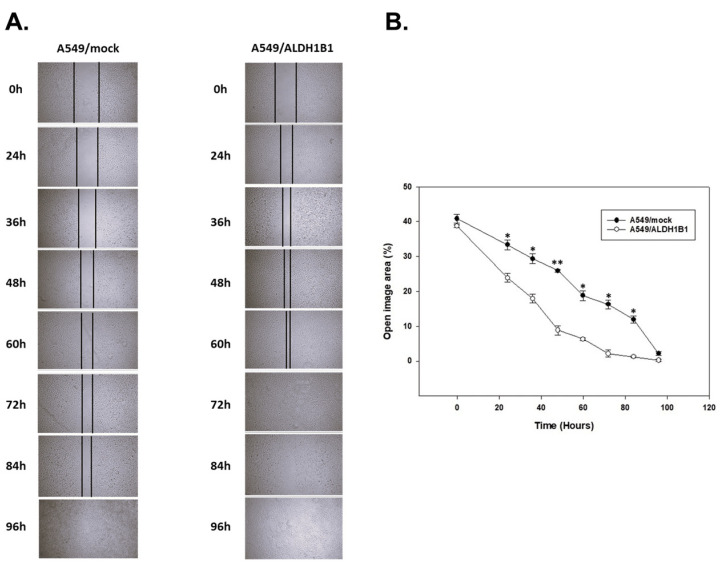
ALDH1B1 is associated with increased migratory potential in A549 cells. (**A**) Scratch assay in A549/mock (left panel) and A549/ALDH1B1 (right panel) cells. Cell migration was monitored under an optical microscope (magnification 20×) at the indicated time points. (**B**) Quantification of the percentage of wound closure by ImageJ software Version 1.54 analysis. Data are presented as mean ± SD of three independent experiments. * *p* < 0.05, ** *p* < 0.01.

Finally, we evaluated the migratory potential of the A549 isogenic cell line pair using the scratch assay. Our findings indicated that the A549/ALDH1B1 cells showed increased migration compared to the A549/mock cells, as demonstrated in Figure 6A. The percentage of wound closure for the ALDH1B1-overexpressing cells was approximately two-fold lower compared to the control (mock) cells, indicating the enhanced migratory potential of ALDH1B1-overexpressing cells, as depicted in Figure 6B.

### 2.5. ALDH1B1 Expression Is Correlated with Important CSC Pathway-Related Molecules in Human Adenocarcinoma Clinical Specimens and the Preclinical A549 Model

**Figure 7:**
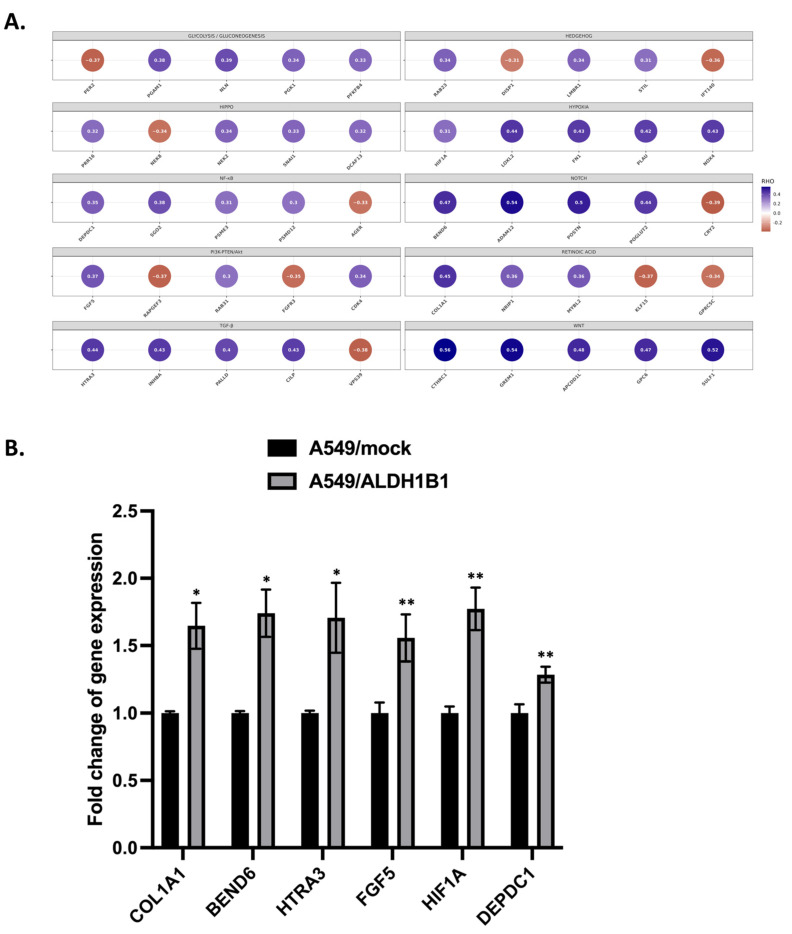
Correlation of ALDH1B1 expression with CSC-related pathways in lung adenocarcinoma. (**A**) Analysis of *ALDH1B1* gene expression with CSC-related molecules in clinical samples of lung adenocarcinoma (RNA-seq, TCGA). A heat map displays the Spearman correlation coefficient for pairwise comparisons. (**B**) Effect of ALDH1B1 overexpression on mRNA levels of selected genes involved in various CSC-related pathways in A549 cells. Total RNA from A549/mock and A549/ALDH1B1 cells was extracted, and gene expression levels were quantified using real-time PCR. Fold changes were determined using the comparative ΔΔCt method with *β-actin* as the internal control for normalization. Results are represented as mean ± SD from at least three independent experiments that were performed. * *p* < 0.05, ** *p* < 0.01.

To further validate the association of ALDH1B1 with the CSC phenotype, we first constructed a list (approximately 3400) with molecules involved in several CSC-related signaling pathways, such as Wnt, Notch, JAK/STAT, Hedgehog, retinoic acid, Hippo, NF-κΒ, hypoxia, TGF-β and PI3K/PTEN-AKT and glycolysis/gluconeogenesis. Subsequently, we investigated the association between ALDH1B1 gene expression and the expression of CSC-related molecules using data from 507 clinical cancer samples data downloaded from the TCGA database. This analysis utilized Spearman’s rank correlation coefficient (Spearman’s rho). In Figure 7A, we present a short list of five genes per pathway that exhibited a substantial and statistically significant correlation (rho > +0.3 and rho < −0.3) with ALDH1B1 gene expression. The full list of the Spearman’s rho analysis results is presented in Supplementary Materials (Tables S1–S13). More specifically, ALDH1B1 was significantly positively correlated with PRR16 (rho = 0.319, *p* < 0.0001) (Table S1), NEK2 (rho = 0.337, *p* < 0.0001) (Table S1), SNAI1 (rho = 0.332, *p* < 0.0001) (Tables S1 and S7–S9), DCAF13 (rho = 0.322, *p* < 0.0001) (Table S1), RAB23 (rho = 0.344, *p* < 0.0001) (Table S2), LMBR1 (rho = 0.337, *p* < 0.0001) (Table S2), STIL (rho = 0.309, *p* < 0.0001) (Table S2), HIF1A (rho = 0.313, *p* < 0.0001) (Tables S3, S9 and S12), LOXL2 (rho = 0.444, *p* <0.0001) (Table S3), FN1 (rho = 0.432, *p* < 0.0001) (Table S3), PLAU (rho = 0.424, *p* < 0.0001) (Table S3), NOX4 (rho = 0.429, *p* < 0.0001) (Table S3), FGF5 (rho = 0.371, *p* < 0.0001) (Table S5), RAB31 (rho = 0.305, *p* < 0.0001) (Table S5), CDK4 (rho = 0.345, *p* < 0.0001) (Table S5), COL1A1 (rho = 0.454, *p* < 0.0001) Tables S6 and S7), NRIP1 (rho = 0.356, *p* < 0.0001) (Table S6), MYBL2 (rho = 0.363, *p* < 0.0001) (Table S6), CTHRC1 (rho = 0.555, *p* < 0.0001) (Table S7), GREM1 (rho = 0.545, *p* < 0.0001) (Table S7), APCDD1L (rho = 0.476, *p* < 0.0001) (Table S7), GPC6 (rho = 0.469, *p* < 0.0001) (Table S7), SULF1 (rho = 0.519, *p* < 0.0001) (Table S7), HTRA3 (rho = 0.438, *p* < 0.0001) (Table S8), INHBA (rho = 0.434, *p* < 0.0001) (Table S8), PALLD (rho = 0.4, *p* < 0.0001) (Table S8), CILP (rho = 0.426, *p* < 0.0001) (Table S8), BEND6 (rho = 0.465, *p* < 0.0001) (Table S9), ADAM12 (rho = 0.535, *p* < 0.0001) (Table S9), POSTN (rho = 0.5, *p* < 0.0001) (Table S9), POGLUT2 (rho = 0.437, *p* < 0.0001) (Table S9), DEPDC1 (rho = 0.352, *p* < 0.0001) (Table S10), SGO2 (rho = 0.384, *p* < 0.0001) (Table S10), PSME3 (rho = 0.309, *p* < 0.0001) (Tables S2, S9 and S10), PSMD12 (rho = 0.304, *p* < 0.0001) (Tables S2, S9 and S10), PGAM1 (rho = 0.382, *p* < 0.0001) (Tables S11 and S12), NLN (rho = 0.390, *p* < 0.0001) (Table S11), PGK1 (rho = 0.341, *p* < 0.0001) (Tables S3 and S12), and PFKFB4 (rho = 0.328, *p* < 0.0001) (Tables S3 and S12). Furthermore, ALDH1B1 is significantly negatively correlated with NEK8 (rho = −0.336, *p* < 0.0001) (Table S1), DISP1 (rho = −0.309, *p* < 0.0001) (Table S2), IFT140 (rho = −0.355, *p* < 0.0001) (Table S2), STAT6 (rho = −0.369, *p* < 0.0001) (Table S4), RAPGEF3 (rho = −0.371, *p* < 0.0001) (Table S5), FGFR3 (rho = −0.347, *p* < 0.0001) (Tables S5 and S7), KLF15 (rho = −0.369, *p* < 0.0001) (Tables S6 and S7), GPRC5C (rho = −0.337, *p* < 0.0001) (Table S6), VPS39 (rho = −0.376, *p* < 0.0001) (Table S8), CRY2 (rho = −0.386, *p* < 0.0001) (Tables S9 and S11), AGER (rho = −0.331, *p* < 0.0001) (Tables S3, S9 and S10) and PER2 (rho = −0.372, *p* < 0.0001) (Table S11) (Figure 7A, Supplementary Materials Tables S1–S12). Additionally, we employed Spearman’s rho to correlate ALDH1B1 mRNA expression with the expression of various cell surface markers and other ALDH isoforms, as shown in Table S13. ALDH1B1 was significantly positively correlated with the cell surface marker CD90 (rho = 0.435, *p* < 0.0001) and ALDH1L2 (rho = 0.361, *p* < 0.0001), while strong negative correlation was observed for ALDH2 (rho = −0.309, *p* < 0.0001), ALDH3B1 (rho = −0.345, *p* < 0.0001), and ALDH5A1 (rho = −0.330, *p* < 0.0001) (Table S13).

Next, we further confirmed the ALDH1B1 correlation with CSC-related molecules, depicted by Spearman’s rho in the 507 TCGA clinical lung adenocarcinoma specimens in vitro, using the A549 isogenic cell line pair. Interestingly, A549/ALDH1B1 exhibited increased transcriptional expression levels of COL1A1 (1.7-fold), BEND6 (1.8-fold), HTRA3 (1.8-fold), FGF5 (1.6-fold), HIF1A (1.8-fold) and DEPDC1 (1.3-fold) (Figure 7B), compared to the A549/mock cells.

## 3. Discussion

To date, the physiological and pathophysiological roles of ALDH1B1 remain undefined. However, recent investigations have contributed to a more comprehensive characterization of its enzymatic functionalities. ALDH1B1 is particularly significant as a biomarker for colon cancer [7]. It may also act as a prognostic biomarker in various cancer types, correlating with immune infiltration [10]. Additionally, ALDH1B1 has been implicated in several biological processes, including antiviral response [14], pancreatic β-cell development (in mouse models) [15], the maintenance of sperm motility (in horses) [16], and the metabolism of ethanol and retinaldehyde (in humans) [17]. Further research has revealed its association with diabetes, colorectal cancer, pancreatic cancer and other types of cancer such as osteosarcoma and lung cancer [1]. ALDH1B1 has emerged as a potential marker associated with chemoresistance. The expression profiles of various ALDH subtypes were studied in A549 and cisplatin-resistant A549/DDP cell lines reavealing significantly elevated levels of ALDH1B1 mRNA and protein in A549/DDP cells, further suggesting a possible link between ALDH1B1 expression and cisplatin resistance in these cellular models [8]. Concurrently, ALDH1B1 was found to be associated with lung tumorigenesis, as evidenced by its enhanced transcriptional expression levels in TCGA lung adenocarcinoma clinical samples compared to normal lung tissue [9].

The main aim of our study was to elucidate the role of ALDH1B1 in human lung adenocarcinoma. For this reason, we generated an isogenic pair of A549 cell lines that differed only in terms of ALDH1B1 expression, which was shown to be correlated with altered cell morphology, lower cell proliferation rate and clonogenic efficiency. Furthermore, ALDH1B1 conferred an enhanced chemoresistant phenotype against both etoposide and cisplatin and promoted migration and EMT through SNAI2, ZEB2 and TWIST1 upregulation in A549 cells. Finally, the correlation of ALDH1B1 with molecules involved in CSC-related pathways (Wnt, Notch, PI3K/Akt, Hedgehog, retinoic acid, Hippo, NF-κΒ, TGF-β, glycolysis/gluconeogenesis) was confirmed by analyzing lung adenocarcinoma clinical specimens obtained by TCGA.

We demonstrated that ALDH1B1 induced cell morphological changes in A549 cells, which is in line with our previous observations of ALDH1B1-induced morphological alterations in human colorectal adenocarcinoma (HT29) cells. ALDH1B1-overexpressing HT29 cells appeared more elongated with lower internal structural complexity [3]. The ALDH1B1-overexpressing A549 cells exhibited a more elongated morphology in contrast to the A549/mock cells, which is likely attributed to the activation of EMT, a process associated with enhanced migratory capacity and an increased vimentin area leading to the elongation of the cell nucleus and cytoplasm [18,19]. In addition, the A549/ALDH1B1 cells displayed slower proliferation rates and reduced colony-formation capacity compared to the A549/mock cells. A similar effect of ALDH1B1 expression was recently demonstrated in HT29 cells, in which p53-mediated G2/M cell cycle arrest potentially triggered slower proliferation of ALDH1B1-overexpressing cells [3]. Further studies have elucidated the impact of ALDH1 on cellular processes such as proliferation, invasion and migration. In instances where ALDH1A3 expression was inhibited in human cancer cell lines, an opposite effect on both cell proliferation and invasion was noted, which was correlated with the differential expression of the CXC chemokine receptor 4 [20]. ALDH1 expression was also associated with CSC characteristics in cervical carcinoma, with ALDH-positive cell populations exhibiting signigicantly higher levels of cell proliferation, sphere formation capacity and migratory potential [21].

On another note, the ALDH1B1-overexpressing A549 cells demonstrated increased resistance against several chemotherapeutic agents, such as etoposide and cisplatin. This finding is consistent with previous studies indicating that ALDH1B1 overexpression induced chemoresistance against etoposide, doxorubicin and 5-fluorouracil (5-FU) [3], which could be associated with the protein’s involvement in the DNA damage response [4]. However, ALDH1B1 could contribute to chemoresistance through multiple mechanisms, including EMT induction, regulation of the cell cycle and interaction with key signaling CSC-related pathways, such as Wnt/*β*-catenin, Notch and PI3K/Akt [3,5]. Interestingly, cisplatin-resistant ovarian cell lines (SKB-R3 and A2780) demonstrated increased numbers of ALDH+ cells [22], and cisplatin-resistant A549 cells showed enhanced transcriptional and translational expression levels of ALDH1B1 [8]. It is noteworthy that ALDH+ cells, with stem cell traits, were implicated in conferring resistance to doxorubicin and etoposide in Ewing sarcoma [23]. Similarly, ALDH3A1-overexpressing breast cancer MCF7 cells exhibited increased tolerance to doxorubicin, 5-FU and etoposide [24]. The enhanced chemoresistant phenotype of A549/ALDH1B1 cells and the mechanistic link between EMT and chemoresistance [25] prompted us to further investigate the effect of ALDH1B1 on EMT transcription factors and regulators. The EMT process is widely considered as the main mechanism by which cancer cells acquire stemness and invasive characteristics, which are requisite for migration, distant invasion and subsequent metastasis [26]. We demonstrated that ALDH1B1 induces EMT through SNAI2, ZEB2 and TWIST1 upregulation, resulting in the down-regulation of E-cadherin and promotion of migration in A549 cells. Our findings are in agreement with previous experimental data indicating the ZEB1-driven EMT induction and the faster migration of the ALDH1B1-overexpressing HT29 cells [2]. The acquisition of the EMT phenotype was also confirmed through the increased levels of vimentin and SNAIL and subsequent down-regulation of E-cadherin in the ALDH_bright_ MKN-45 and SGC-7901 cells [27]. Furthermore, knocking down ALDH1A1 and ALDH3A1 resulted in decreased migratory capacity in A549 cells [28]. In another study, although the knockdown of ALDH1A1 led to decreased migration of MDA-MB-468 and SUM159 cells, ALDH1A3 knockdown increased the migratory potential of these cells [29]. Although the decreased proliferation rate of A549/ALDH1B1 cells combined with their increased migratory potential may appear contradictory, they may be two sides of the same coin [3,30].

As ALDH1B1 appeared to be involved in CSC-associated features in A549 cells, we set out to analyze the correlation between ALDH1B1 and molecules implicated in CSC-related pathways (Wnt, Notch, hypoxia, Hedgehog, Retinoic acid, Hippo, NF-κΒ, TGF-β and PI3K/AKT, glycolysis/gluconeogenesis). CSCs are a small subpopulation of cells within the tumor with stem-like properties, including the capacity for self-renewal and the ability to undergo asymmetrical cell division, multilineage differentiation potential, the ability to maintain a non-differentiated-quiescent state and the capability to initiate new heterogeneous tumors and demonstrate phenotypic plasticity [1]. Many signaling pathways are implicated in regulating CSCs, such as Notch, Wnt/β-catenin, Sonic hedgehog (Shh), TGF-β, JAK/STAT3, glycolysis, hypoxia, Hippo, retinoic acid and NF-κB [31,32,33]. Notch, Hedgehog and Wnt signaling pathways may synergistically induce the expression of specifically lung CSC markers and activate EMT, thus enabling cancer cells to acquire stemness and chemoresistance [34]. ALDH1B1 was shown to be statistically significantly correlated with many molecules involved in CSC-related pathways (Supplementary Materials), as analyzed in 507 lung adenocarcinoma clinical data derived from TCGA. This correlation was also confirmed in vitro by the transcriptional upregulation of selected molecules (COL1A1, BEND6, HTRA3, FGF5, HIF1A, DEPDC1) in A549/ALDH1B1 cells. These findings highlight ALDH1B1 as a lung CSC-associated molecule and a potential therapeutic target for lung adenocarcinoma, thereby providing a framework for further research into its mechanisms in lung cancer biology. High levels of ALDH1B1 expression could be used to identify lung cancer patients who are at higher risk of poor outcomes. While current inhibitors like disulfiram [35] and other ALDH-targeting compounds show potential, more selective and potent ALDH1B1 inhibitors are needed to reduce off-target effects and improve therapeutic outcomes. Recently, guanidinyl antagonists of ALDHs (IGUANAs) were discovered as specific inhibitors of ALDH1B1 with efficient potency against cancer cells in preclinical studies, inducing selective growth inhibition of colon cancer spheroids and organoids [36]. Combining ALDH1B1 inhibition with other therapies, such as chemotherapy, targeted therapies, or immunotherapies, could offer more effective strategies for lung adenocarcinoma in the future; however, these strategies require further exploration. Given the important role of ALDH1B1 in ethanol metabolism, targeting ALDH1B1 strategies may also hold promise for improving cancer treatment outcomes, particularly in patients with high ethanol consumption, by targeting key metabolic (e.g., normalizing the NADH/NAD+ ratio and shifting cancer cell metabolism) and protective mechanisms (e.g., reducing cancer stem cell populations, chemoresistance and ethanol-induced inflammatory and immunosuppressive effects) in cancer cells [37,38,39].

## 4. Materials and Methods

### 4.1. Materials

The A549 human lung cancer cell line was obtained from ATCC (Manassas, VA, USA). Culture medium, fetal bovine serum (FBS), penicillin/streptomycin (100×) solution and trypsin were sourced from Biosera (Boussens, France). Cell culture flasks and plates, Falcon tubes and Eppendorf tubes were obtained from SPL Life Sciences (Pocheon, Gyeonggido, South Korea). NucleoZOL was purchased from Macherey-Nagel (Düren, Germany). Primers, dNTPs, random hexamers and PrimeScript reverse transcriptase were acquired from Invitrogen (ThermoFischer Scientific, Waltham, MA, USA), and KAPA SYBR Fast Master Mix was obtained from Kapa Biosystems (Hoffmann-La Roche, Basel, Switzerland). Propidium iodide (PI) was sourced from Biotium (Landing Parkway, Fremont, CA, USA).

### 4.2. Cell Culture

Human lung cancer adenocarcinoma (A549) and human embryonic kidney (HEK) 293 cells were obtained from American Type Culture Collection (ATCC) (Manassas, VA, USA). Both cell lines were maintained in high glucose Dulbecco’s modified Eagle’s medium (DMEM) supplemented with 10% FBS, 100 units/mL penicillin and 100 μg/mL streptomycin. The A549 isogenic cell line pair was cultured with the extra addition of 1.5 μg/mL puromycin. Cells were cultivated at 37 °C with 5% CO_2_ in a humidified incubator.

### 4.3. Lentiviral Transduction

Lentiviral transduction was performed as described previously [3].

### 4.4. Real-Time PCR

Total RNA was isolated using NucleoZOL reagent following the manufacturer’s protocol. For cDNA synthesis, 4 μg of total RNA was reverse-transcribed using SuperScript™ First-Strand Synthesis kits as per the manufacturer’s instructions. Real-time PCR was carried out with KAPA SYBR Fast Master Mix according to the manufacturer’s protocol. Reactions were performed on the Applied Biosystems Step One instrument (Thermo Fisher Scientific, Waltham, MA, USA). Primer sequences are detailed in Table 5. Each reaction was performed in triplicate across three independent experiments. Gene expression levels were normalized to *β-actin* using the 2^−ΔΔCT^ method.

### 4.5. Flow Cytometry Analysis

#### 4.5.1. Assessment of Transfection Efficiency

Cells (A549/mock and A549/ALDH1B1) were washed with PBS, stained with PI for 3 min, and then transferred into fluorescence-activated cell sorting (FACS) tubes (BD Biosciences, Franklin Lakes, NJ, USA). Analysis was performed using an Attune NxT flow cytometer (Thermo Fisher Scientific, Waltham, MA, USA). The transfection efficiency of A549 carrying the ALDH1B1 gene was determined by comparing the percentage of GFP-stained cells (A549/ALDH1B1) to the control (A549/mock) cells.

#### 4.5.2. Flow Cytometric Analysis of Cell Size

PBS-washed cells were analyzed using the Attune NxT flow cytometer. Flowjo software v10.10 (FlowJo LLC, Ashland, OR, USA) was used to generate histograms of forward scatter (FCS) and side scatter (SCS) for A549/mock and A549/ALDH1B1 cells. Cell morphology was evaluated based on median fluorescence intensity values of FSC and SSC, as previously described [3].

#### 4.5.3. Cell Counting and Growth Rate Determination

Cell counting was performed by flow cytometry analysis, and the growth rate of cell cultures was estimated as described previously [3].

### 4.6. Fluorescence Microscopy

Cells (2 × 10^5^) were plated on coverslips and, after 24 h, fixed with 4% formaldehyde in PBS for 20 min. After washing thrice with PBS, a neutralization step was conducted using 1 M of glycine (pH 8.5). Nuclei were counterstained with 4′-6-diamidino-2-phenylindole (DAPI) (1 μg/mL), followed by another round of PBS washing (×3). Finally, cells were mounted with Fluoromount-G™ (Thermo Fisher Scientific, Waltham, MA, USA) and observed on a Nikon ECLIPSE E200 fluorescence microscope equipped with 40× and 100× lenses. Images were captured and analyzed using image analysis software (ImageJ; National Institute of Health (NIH), Bethesda, MD, USA).

### 4.7. ALDH1B1 Enzymatic Activity

ALDH1B1 enzymatic activity was assessed following established methods [13]. Briefly, a reaction mixture containing 75 mM sodium pyrophosphate pH 8.0, 1 mM pyrazole, 1 mM NAD^+^ and 50 μL of A549 cell lysates was prepared as a blank. The reaction was initiated by adding 100 μL of different substrates (1 mM acetaldehyde and 1 mM propionaldehyde) sequentially. The enzymatic activity of ALDH1B1 was determined by monitoring NADH production over 5 min at room temperature.

The following is the general reaction catalyzed by ALDHs:

Aldehyde + NAD(P)^+^ + H_2_O → Carboxylic acid + NAD(P)H + H^+^, (1)

### 4.8. Colony Formation Assay

The colony formation assay was conducted according to previously described protocols [3].

### 4.9. Sulforhodamine B (SRB) Assay

The SRB assay was performed as previously detailed [3]. Briefly, 3 × 103 A549 cells per well were cultured in 96-well plates. After incubation for one day, cells were treated with increasing concentrations of chemotherapeutic agents (etoposide, 0–500 μM and cisplatin, 0–50 μM) for 48 h. Following treatment, cells were fixed with ice-cold trichloroacetic acid (TCA) and stained with SRB dye in 1% (*v*/*v*) acetic acid. The bound dye was dissolved in a 10 mM Tris base, and absorbance was measured at 570 nm using a microplate reader (Enspire, Perkin Elmer, Waltham, MA, USA). Percent (%) cell viability was calculated using the following formula:

% cell viability = mean OD_570_ sample/mean OD_570_ control × 100

The IC_50_ values (half maximal inhibitory concentration) were calculated by Sigma Plot Software v.10 (Systat, San Jose, CA, USA) via a four-parameter logistic curve fitting method.

### 4.10. Sphere Formation Assay

The sphere-formation assay was conducted following previously established procedures [3]. Spheres were formed within 8–12 days, with medium changes occurring every 2 to 3 days. First-generation spheres were used for all subsequent experiments.

### 4.11. Scratch Assay

A549/mock and A549/ALDH1B1 (4 × 10^5^) cells were seeded in 6-well plates one day prior to the experiment. Plates were washed twice with PBS, and scratches were made on the confluent cell monolayer using a sterilized pipette tip. Cells were photographed at specified time points using a ZEISS Primovert light microscope (Zeiss, Göttingen, Germany) equipped with a digital camera (Axiocam ERc 5 s). Multiple photographs were analyzed for each time point using ImageJ software to calculate the average percentage of wound area (% open image area).

### 4.12. Data Acquisition and Bioinformatics Analysis

Clinical data from 507 lung adenocarcinoma patient samples were obtained from The Cancer Genome Atlas (TCGA) database via cBioportal.org (accessed on 1 February 2022) [11,12]. These data were utilized to investigate the correlation between ALDH1B1 and genes associated with CSC-related pathways. TCGA provides comprehensive data on cancer tissues, encompassing RNA-seq, mass spectroscopy, miRNA mutation, methylation and other types of data across 33 tumor types. The analysis focused on RNA-seq data from 507 lung cancer clinical samples and the Spearman’s correlation analysis was conducted on all available log2(x + 1) transformed RNA-seq data. Correlation heat maps were generated using R language and the corrplot package.

### 4.13. Statistical Analysis

All statistical analyses were performed using GraphPad Prism software, version 10 (San Diego, CA, USA). Data are presented as mean ± SD. Student’s *t*-test was employed for comparing results between two groups, while two-way ANOVA followed by Tukey’s multiple comparison tests was used to analyze two variables among multiple groups. Spearman’s rank correlation coefficient was calculated to assess the strength and direction of a relationship between *ALDH1B1* gene expression and genes involved in CSC-related pathways. A *p*-value < 0.05 was considered as statistically significant, and each experimental design was conducted in at least three independent experiments.

## 5. Conclusions

In conclusion, overexpression of ALDH1B1 in A549 cells resulted in altered cell morphology, slower proliferation rates and clonogenic efficiency, as well as enhanced chemoresistance against etoposide and cisplatin. In addition, A549/ALDH1B1 cells demonstrated increased migratory potential, perhaps by upregulating key molecules of the EMT process. To investigate the role of ALDH1B1 in the clinical setting, a Spearman’s correlation coefficient analysis was performed in 507 lung adenocarcinoma samples which further validated that ALDH1B1 is statistically significantly positively correlated with many molecules involved in CSC-related (Wnt, Notch, Hedgehog, JAK/STAT, PIEK-PTEN/Akt) and metabolism-associated (glycolysis) signaling pathways, suggesting its crucial role in the CSC phenotype of lung adenocarcinoma cells. Our findings support the notion that ALDH1B1 contributes to the CSC phenotype and confers resistance to chemotherapy. In this regard, selective inhibition of ALDH1B1 may be considered among the promising strategies for CSC-directed therapeutics in lung adenocarcinoma cancer. Further studies are required to enhance our understanding of the underlying role of ALDH1B1 in the CSC phenotype and its association with lung cancer progression.

## Figures and Tables

**Table 1:** ALDH activity (nmol NADH/min.mg of protein) in A549/mock and A549/ALDH1B1 cell lysates.

Substrate	A549/Mock	A549/ALDH1B1	Statistical Significance
**Acetaldehyde**	71.62 ± 13.88	138.21 ± 18.74	**
**Propionaldehyde**	70.71 ± 10.61	128.95 ± 31.07	**

Results are represented as mean ± S.D. of three independent experiments. ** *p* < 0.01.

**Table 2:** Median fluorescence intensity for forward scatter (FSC) and side scatter (SSC) parameters.

	Parameter	A549/Mock	A549/ALDH1B1	Statistical Significance
**Median Fluorescence Intensity**	**FSC**	304,842.7 ± 12,480.51	374,101.3 ± 23,520.44	**
	**SSC**	213,589.3 ± 8375.28	224,085.3 ± 13,956.89	-

Results are shown as mean ± SD of three independent experiments. ** *p* < 0.01.

**Table 3:** Doubling time (tD) and growth rates (GR) of A549/ALDH1B1 and A549/mock cells.

Sample	(tD) (h)	GR
**A549/mock**	24.33 ± 0.73	0.0285 ± 0.02564
**A549/ALDH1B1**	27.04 ± 0.54	0.02564 ± 0.0005
**Statistical significance**	**	**

Results are expressed as mean ± SD of three independent experiments. ** *p* < 0.01.

**Table 4:** IC_50_ values of etoposide and cisplatin (48 h).

Cells	Etoposide (μΜ)	Cisplatin (μΜ)
**A549/mock**	10.02 ± 4.10	11.89 ± 1.63
**A549/ALDH1B1**	29.74 ± 8.99	22.52 ± 5.37
**Statistical significance**	*	**

Results are expressed as mean ± SD of three independent experiments. * *p* < 0.05, ** *p* < 0.01.

**Table 5:** Primers for real-time PCR comparative quantification.

Gene	Forward Primer	Reverse Primer
*β-* *actin*	GCGCGGCTACAGCTTCA	CTTAATGTCACGCACGATTTCC
*ALDH1B1*	AGCCTCTGTTCAAGTTCAAG	CCTTAAAGCCTCCGAATGG
*CDH1*	TACACTGCCCAGGAGCCAGA	TGGCACCAGTGTCCGGATTA
*SNAI1*	ACTATGCCGCGCTCTTTCCT	GGTGGGGTTGAGGATCTCCG
*SNAI2*	CTACAGCGAACTGGACACAC	TGTGGTATGACAGGCATGGAG
*VIM*	TGAGTACCGGAGACAGGTGCAG	TAGCAGCTTCAACGGCAAAGTTC
*ZEB1*	CGAGTCAGATGCAGAAAATGAGCAA	ACCCAGACTGCGTCACATGTCTT
*ZEB2*	ACTATGGGGCCAGAAGCCAC	CTGCATGACCATCGCGTTCC
*TWIST1*	AGCTACGCCTTCTCGGTCTG	TGGGAATCACTGTCCACGGG
*COL1A1*	TCTGCGACAACGGCAAGGTG	GACGCCGGTGGTTTCTTGGT
*BEND6*	CCGAGGTTGGGGCTTTGAAGA	ATCTGTCTGCACGATCTTCTGCAT
*HTRA3*	CAGCCGTGGTCCACATAGAG	TTGATGGTGGCAATGTCCGA
*FGF5*	CTGCAAGTTCAGGGAGCGTTT	ATACCACTCCCGCCCTGTTT
*HIF1A*	ACCCTAACTAGCCGAGGAAGA	TGGGTGAGGAATGGGTTCAC
*DEPDC1*	ACTTCCCCCACCAAATCGTAG	TGACCTCGTACCCATTGCAT

## Data Availability

Part of the results presented in this study are based on data generated by the TCGA Research Network, which were downloaded from cBioportal.org (assessed on 1 February 2022).

## Supplementary Materials

The following supporting information can be downloaded at https://www.mdpi.com/article/10.3390/ijms251910301/s1.
