# Supplementary material for: Exploring the Role and Pathophysiological Significance of Aldehyde Dehydrogenase 1B1 (ALDH1B1) in Human Lung Adenocarcinoma
Source: Int J Mol Sci. 2024 Sep 25;25(19):10301. doi: 10.3390/ijms251910301 (PMC11477306; doi:10.3390/ijms251910301)
Supplement: Supplementary file 1 [file ijms-25-10301-s001.zip › ijms-3088344-supplementary.pdf]

Table S1: Correlation of mRNA levels between ALDH1B1 and Hippo pathway-related molecules

Gene name	Secondary gene name	Rho	P-value
DLG5	KIAA0583	-0.038	0.389728177
LATS1	WARTS	0.053	0.234355158
STK4	KRS2	0.022	0.615850506
STK3	KRS1	0.071	0.10823499
YAP1	YAP65	-0.076	0.085372568
WWC1	KIAA0869	-0.281	9.98E-11
SAV1	WW45	0.092	0.037340205
WWTR1	TAZ	0.181	4.09348E-05
NF2	SCH	0.057	0.196150444
LATS2	KPM	0.017	0.697627035
MARK3	CTAK1	-0.075	0.090638979
MOB1B	MOB4A	-0.030	0.495698339
NPHP4	KIAA0673	-0.119	0.007052621
TEAD4	RTEF1	0.268	7.51E-10
LIMD1		-0.281	1.04E-10
NEK8	JCK	-0.336	6E-15
MOB1A	C2orf6	0.265	1.2E-09
AJUBA	JUB	0.076	0.08562126
WTIP		-0.087	0.050406947
OGG1	MMH	0.163	0.00021634
TEAD2	TEF4	0.044	0.32331829
TEAD1	TCF13	0.249	1.18E-08
WWC2	BOMB	-0.124	0.005165809
TJP1	ZO1	-0.111	0.011760468
TEAD3	TEAD5	-0.233	0.000000102
BMP6	VGR	-0.119	0.007217264
PJA2	KIAA0438	-0.080	0.072638462
PALS1	MPP5	-0.032	0.464445795
SMAD3	MADH3	-0.103	0.020160058
TIAL1		0.038	0.388887586
SOX11		0.165	0.000188159
SCHIP1		0.143	0.001180965
FAT4	CDHF14	-0.060	0.17712782
GPC3	OCI5	-0.162	0.000247942
MAP2K3	MEK3	-0.259	2.81E-09
HNF4A	HNF4	-0.088	0.046082454
CDH1	CDHE	0.009	0.835724511
AMOTL1		-0.014	0.756503601
DCAF1	KIAA0800	0.020	0.64449894
DDX17		-0.283	7E-11
ESR1	ESR	0.152	0.000570639
FOXO1	FKHR	-0.152	0.000567899
FRMD1		0.056	0.209076656
CIT	CRIK	-0.153	0.000522094
PRR16		0.319	1.73E-13
MOB3B	C9orf35	0.140	0.001534942
MAPK14	CSBP	0.091	0.039251135

NPHP3	KIAA2000	-0.181	3.82423E-05
DCHS1	CDH19	0.166	0.000163233
SMAD2	MADH2	0.030	0.50360339
SCRIB	CRIB1	-0.040	0.363084721
SNAI1	SNAH	0.332	1.35E-14
SHANK2	CORTBP1	-0.299	5.17E-12
IQCJ-SCHIP1		NA	NA
VGLL4	KIAA0121	0.157	0.000382463
PTPN14	PEZ	0.032	0.465420167
TJP2	X104	-0.228	0.000000197
WWOX	FOR	-0.064	0.150270343
WWC3	KIAA1280	-0.191	1.35987E-05
YWHAB		0.133	0.002520327
NEK2	NEK2A	0.337	5.13E-15
VEPH1	KIAA1692	-0.249	1.15E-08
AMOTL2	KIAA0989	0.014	0.749337174
CDH2	CDHN	0.304	2.47E-12
CEP250	CEP2	0.009	0.838869126
YWHAE		0.049	0.271449296
AKR1B10	AKR1B11	0.019	0.665714232
DCAF13	WDSOF1	0.322	9.75E-14
DVL2		-0.011	0.797679756
CASP3	CPP32	0.160	0.000282485
AMOT	KIAA1071	-0.016	0.714564532

Table S2: Correlation of mRNA levels between ALDH1B1 and Hedgehog pathway-related molecules

Gene name	Secondary gene name	Rho	P-value
IHH		-0.283	7.74E-11
DHH		-0.018	0.680240618
SHH		-0.370	5.68E-18
HHAT	MART2	-0.256	4.19E-09
HHIP	HIP	-0.112	0.011338216
HHATL	C3orf3	-0.101	0.022520932
MOSMO	ATTHOG	-0.047	0.284473246
PTCH1	PTCH	-0.281	1.03E-10
SUFU	UNQ650/PRO1280	-0.001	0.975215845
GLI3		0.215	0.000000931
GLI2	THP	-0.030	0.493651169
GLI1	GLI	-0.124	0.00504254
KIF7	UNQ340/PRO539	0.056	0.208539364
SMO	SMOH	0.074	0.093949383
FOXC1	FKHL7	0.133	0.00254588
PTCH2	UNQ560/PRO1121/PRO57079	-0.149	0.000746787
CDON	CDO	-0.090	0.041559986
SPOP		0.108	0.01468955
ULK3		-0.237	6.33E-08
DZIP1	DZIP	0.170	0.000117706

SCUBE2	CEGP1	-0.136	0.002068991
IFT172	KIAA1179	-0.348	5.71E-16
IFT57	DERP8	-0.082	0.064401625
PSMD11		0.271	4.68E-10
PSMB9	LMP2	0.157	0.000370623
PKD2L1	PKD2L	-0.108	0.014311761
PKD1L1	UNQ5785/PRO19563	-0.090	0.042985141
STK36	KIAA1278	-0.249	1.15E-08
MGRN1	KIAA0544	-0.203	3.84182E-06
RAB23	HSPC137	0.344	1.37E-15
RAB34	RAB39	0.159	0.000319131
RABEP2	RABPT5B	-0.271	4.7E-10
RBX1	RNF75	0.036	0.417621393
PSMC4	MIP224	0.115	0.009467971
SEM1	C7orf76	0.149	0.000718446
TMEM17		-0.170	0.000115469
PSMA7	HSPC	0.187	2.16913E-05
PSMA6	PROS27	0.280	1.26E-10
PSMC5	SUG1	0.007	0.880719704
UBA52	UBCEP2	0.093	0.035953009
TMEM107	DC20	-0.238	5.13E-08
SPOPL		0.068	0.127555992
PSMB6	LMPY	0.012	0.794233119
PSMB4	PROS26	0.082	0.063307255
SDCCAG8	CCCAP	-0.107	0.015980729
TTC21B	IFT139	-0.084	0.0572582
TUBB1		-0.271	4.62E-10
LRP2		-0.162	0.000240837
LRRK2	PARK8	-0.274	2.95E-10
PRKAR2B		-0.048	0.282003657
PRKAR1B		0.142	0.00130574
PRKACB		-0.002	0.963106942
EVC2	LBN	0.029	0.507396132
HSPB11	C1orf41	-0.019	0.673898024
CC2D2A	KIAA1345	-0.094	0.03308511
C2CD3		-0.061	0.169712406
PRKAR2A	PKR2	0.104	0.018476675
TMEM67	MKS3	0.037	0.403703443
HDAC1	RPD3L1	-0.091	0.041011538
NPHP1	NPH1	-0.091	0.040299653
RPGRIP1L	FTM	0.124	0.004926773
IFT27	RABL4	-0.204	3.43629E-06
ARL6	BBS3	0.109	0.013702119
BBS1	BBS2L2	-0.261	2.25E-09
B9D1	MKSR1	-0.109	0.013410984
BBS4		-0.257	3.64E-09
DISP1	DISPA	-0.309	8.98E-13
CDHR1	KIAA1775	-0.031	0.482653399
MKS1		-0.106	0.016497394
EMX1		0.012	0.790780029
FKBP8	FKBP38	-0.086	0.053575176

PRKACG		0.020	0.647219913
ADCY7	KIAA0037	0.022	0.626771095
CDK8		0.273	3.45E-10
BBS2		-0.226	0.00000024
GAS8	DRC4	-0.287	3.68E-11
ENPP1	M6S1	0.285	5.08E-11
FOXL1	FKHL11	0.019	0.663007455
GNAS	GNAS1	-0.008	0.849420015
FUZ	FY	-0.104	0.019315844
FBXL17	FBL17	-0.151	0.000641565
DYNC2H1	DHC1B	-0.138	0.001806722
HAND2	BHLHA26	0.067	0.133511668
KCTD6		0.132	0.002753841
LMBR1	C7orf2	0.337	5.08E-15
MED1	ARC205	0.170	0.000112179
MEGF8	C19orf49	-0.062	0.160476738
PSMA5		0.126	0.00452948
PSMD14	POH1	0.216	0.000000842
INTU	KIAA1284	-0.089	0.044597135
KCTD11	C17orf36	0.067	0.128360971
DERL2	DER2	-0.071	0.111571713
CEP131	AZI1	-0.001	0.976166637
PTCHD4	C6orf138	-0.193	1.18365E-05
PSMB8	LMP7	0.074	0.095308026
PSMA3	HC8	0.128	0.003732448
PSMD4	MCB1	0.027	0.536171444
PSMB1	PSC5	0.125	0.004842385
PSMF1		0.033	0.461859631
OFD1	CXorf5	-0.290	2.34E-11
PSMC3	TBP1	0.104	0.01911844
SMURF1	KIAA1625	0.009	0.83621235
SMURF2		0.232	0.000000118
PSMB2		0.150	0.000668692
PSMD8		0.101	0.022208619
PSMB11		-0.032	0.464769842
PSMD9		0.183	3.24187E-05
MED6	ARC33	0.143	0.001224691
OS9		-0.226	0.000000259
PSMB3		0.192	1.21706E-05
TMEM231	UNQ870/PRO1886	-0.039	0.375041158
STIL	SIL	0.309	1.01E-12
PSMB5	LMPX	0.173	8.36453E-05
PSMD7	MOV34L	0.132	0.002816502
PSMB7	Z	0.112	0.011489039
PSMB10	LMP10	-0.056	0.202899721
KIAA0586	TALPID3	0.080	0.069919156
SEL1L	TSA305	0.178	5.07852E-05
TUBB4B	TUBB2C	0.029	0.509142738
PSME1	IFI5111	0.090	0.041101063
SOX9		-0.033	0.450849332
TTC23	HCC8	-0.182	3.40568E-05

TTC8	BBS8	0.019	0.667851317
PSMD1		0.159	0.000306201
TUBA3C	TUBA2	0.040	0.362837562
TUBB3	TUBB4	0.362	3.43E-17
TUBA1B		0.338	4.05E-15
PSMD10		0.176	6.42774E-05
PSME3		0.309	9.47E-13
PSMC1		0.113	0.010477901
PDCL	PHLOP1	0.103	0.019462506
GNAS	GNAS1	-0.008	0.849420015
PSMD12		0.304	2.14E-12
TCTN3	C10orf61	0.047	0.286294505
TCTN2	C12orf38	0.026	0.556186898
PSMA8	PSMA7L	0.073	0.098715423
RILPL1	RLP1	0.073	0.101179754
RPS27A	UBA80	0.077	0.082188291
PVR	PVS	0.179	0.000048255
VCP		0.222	0.000000418
TUBA4A	TUBA1	0.214	1.07222E-06
ZNF431	KIAA1969	-0.014	0.75446473
PSMD3		0.210	1.65074E-06
PSMD2	TRAP2	0.264	1.47E-09
RAB10		0.207	2.49494E-06
PSMA2	HC3	0.269	6.62E-10
TUBB2B		0.244	0.000000025
PSMC2	MSS1	0.267	9.01E-10
PSMA1	HC2	0.114	0.01017893
PSMC6	SUG2	0.157	0.000363285
SYVN1	HRD1	-0.098	0.026542235
PSMD5	KIAA0072	0.140	0.001524555
PSME2		0.162	0.000230342
PSMD6	KIAA0107	0.171	0.000104287
TUBB6		0.287	4.01E-11
VSX2	CHX10	0.024	0.588650852
TUBA1A	TUBA3	0.261	2.34E-09
WDR19	IFT144	-0.190	1.60314E-05
TUBA3D		0.006	0.897830231
TEDC1	C14orf80	0.032	0.472415674
WDR11	BRWD2	-0.118	0.007713558
ZNF263	FPM315	-0.077	0.083198681
TUBB2A	TUBB2	0.264	1.42E-09
UBC		0.063	0.157792556
WDR35	IFT121	-0.018	0.680982644
TUBB4A	TUBB4	0.180	4.13479E-05
TCTN1	TECT1	-0.098	0.02766058
PSME4	KIAA0077	0.174	7.64579E-05
UBB		-0.139	0.001591687
TULP3	TUBL3	0.121	0.006225185
WSB1	SWIP1	-0.151	0.000618111
PSMA4	HC9	0.029	0.507181977
PSMD13		0.135	0.002251388

TXNDC15	C5orf14	0.041	0.35079087
TUBD1	TUBD	0.120	0.006598263
TEDC2	C16orf59	0.222	0.000000426
TUBA1C	TUBA6	0.342	2.08E-15
NUMB	C14orf41	-0.152	0.000550091
P4HB	ERBA2L	0.011	0.801252421
RAB8A	MEL	0.104	0.019244459
ERC1	ELKS	0.072	0.105188655
IFT122	SPG	-0.119	0.007226259
IFT140	KIAA0590	-0.355	1.31E-16
IFT52	C20orf9	0.155	0.000458634
IFT88	TG737	-0.214	1.02728E-06
MXRA8		0.229	0.000000174
IQCE	KIAA1023	-0.197	7.71998E-06
ITCH		0.234	8.98E-08
GPR161		0.263	1.71E-09
MED12	ARC240	-0.144	0.001094272
KCTD21		0.026	0.56213605
LZTFL1		-0.031	0.48605857
PRKACA	PKACA	0.104	0.018498861
KIF3A	KIF3	-0.146	0.000976148
MED23	ARC130	-0.106	0.017039482
CUL3	KIAA0617	-0.020	0.650745099
PRKAR1A	PKR1	-0.099	0.025004309
CDC73	C1orf28	0.141	0.001437007
DYRK2		0.270	6.04E-10
DZIP1L		0.082	0.063266026
BOC	UNQ604/PRO1190	0.162	0.000235242
ACTRT1	ARPT1	-0.052	0.244578002
ADCY2	KIAA1060	-0.009	0.839872532
ADCY8		-0.075	0.090443194
ADCY9	KIAA0520	-0.093	0.036648733
CLUAP1	KIAA0643	-0.187	2.22524E-05
CILK1	ICK	-0.082	0.06377815
GLIS2	NKL	0.168	0.000136765
BMP4	BMP2B	0.022	0.621287438
ADCY6	KIAA0422	-0.333	1.14E-14
ADCY1		0.043	0.330470388
ADCY10	SAC	-0.077	0.083892062
ADCY4		-0.112	0.011497992
ADCY3	KIAA0511	0.190	1.50952E-05
GRK2	ADRBK1	-0.127	0.004132689
CIBAR1	FAM92A	0.149	0.000735029
EVC		0.081	0.06869151
ERLEC1	C2orf30	-0.044	0.325135324
ADCY5		-0.032	0.4727589
BBS5		-0.170	0.000114432
ARMC9	KIAA1868	0.049	0.265300609
EFCAB7	KIAA1799	-0.095	0.031514476
BBS7	BBS2L1	0.221	0.00000044

Table S3: Correlation of mRNA levels between ALDH1B1 and hypoxia pathway-related molecules

Gene name	Secondary gene name	Rho	P-value
HIF1AN	FIH1	-0.119	0.007040119
HILPDA	C7orf68	0.370	5E-18
HIF1A	BHLHE78	0.313	4.45E-13
HIF3A	BHLHE17	-0.272	4.32E-10
EGLN1	C1orf12	-0.071	0.109974343
EGLN3		0.256	4.24E-09
EGLN2	EIT6	-0.201	5.02205E-06
EPAS1	BHLHE73	-0.261	2.06E-09
P4HTM	PH4	-0.217	0.000000741
ARNT	BHLHE2	-0.108	0.014345338
HYOU1	GRP170	0.051	0.254814133
HIGD1A	HIG1	0.025	0.568549787
MGARP	C4orf49	0.134	0.002472385
SESN2	Hi95	-0.069	0.120472707
DIPK2A	C3orf58	0.042	0.344319388
TIGAR	C12orf5	0.228	0.000000205
RORA	NR1F1	-0.145	0.000983146
VHL		0.043	0.335529728
HIPK2		-0.012	0.78113415
PDK1	PDHK1	0.262	1.97E-09
DDIT4	REDD1	0.094	0.033502842
VEGFA	VEGF	0.194	9.9617E-06
CITED2	MRG1	-0.219	0.000000601
EP300	P300	-0.039	0.383230831
PKM	OIP3	0.174	7.48452E-05
FAM162A	C3orf28	0.206	2.80179E-06
MTOR	FRAP	-0.012	0.780842348
SIRT2	SIR2L	-0.105	0.017376913
REST	NRSF	0.040	0.371044643
RWDD3	RSUME	-0.004	0.93289121
PLK3	CNK	-0.013	0.772841575
CHCHD2	C7orf17	0.169	0.000129962
NOL3	ARC	-0.173	0.000089994
LOXL2		0.444	5.06E-26
DPP4	ADCP2	0.096	0.030871412
USP19	KIAA0891	-0.139	0.001620322
FUNDC1		-0.005	0.911243114
ADA	ADA1	0.259	3.13E-09
PDK3	PDHK3	0.301	3.58E-12
TM9SF4	KIAA0255	0.037	0.402620619
SCN2A	NAC2	0.108	0.014376688
AK4	AK3	0.308	1.21E-12
HP1BP3		-0.164	0.000203017
KCNB1		-0.035	0.436304453
CEMP1		-0.203	3.85016E-06
EGR1	KROX24	-0.052	0.240416781
FMN2		-0.023	0.598311334

CA9	G250	0.148	0.00078703
BNIP3	NIP3	0.141	0.00140097
TP53	P53	0.029	0.51362934
SIRT1	SIR2L1	0.008	0.850132373
SORL1	C11orf32	-0.147	0.000858722
USP28	KIAA1515	0.014	0.75422625
ZFP36	G0S24	-0.253	6.72E-09
HK2		0.025	0.568038282
NGB		NA	NA
NDRG1	CAP43	0.153	0.000519612
MANF	ARMET	0.088	0.047805926
KLHL20	KLEIP	-0.056	0.207075525
CREBBP	CBP	-0.084	0.057155558
DDIT4L	REDD2	-0.011	0.79778047
ALKBH5	ABH5	-0.089	0.043355659
TRIB3	C20orf97	0.210	1.74392E-06
TNKS2	PARP5B	-0.046	0.298446077
RNF4	SNURF	0.143	0.001198172
TRPA1	ANKTM1	0.387	1.2E-19
EIF3E	EIF3S6	0.102	0.021184481
LRP5	LR3	-0.214	1.07819E-06
KCNK2	TREK	0.203	3.64486E-06
NOTCH1	TAN1	-0.063	0.157203737
ENG	END	-0.056	0.208596283
PFKFB4		0.328	2.86E-14
ING4	My036	-0.147	0.000895382
CARD16	COP	-0.081	0.069183754
ENO1	ENO1L1	0.251	9.16E-09
ANGPTL4	ARP4	0.154	0.000473662
ATR	FRP1	0.119	0.007263411
PFKFB3		0.124	0.005070148
PINK1		-0.206	2.6137E-06
CREB1		0.078	0.078665396
ERO1A	ERO1L	0.288	3.5E-11
EPO		0.007	0.868998451
CUL2		0.285	5.28E-11
FOXO4	AFX	-0.232	0.000000124
CBFA2T3	MTG16	-0.148	0.000816445
CAD		0.236	6.62E-08
RBM3	RNPL	0.060	0.17868543
MT3		0.041	0.354895112
NOTCH3		0.134	0.002454911
TAL1	BHLHA17	-0.205	2.98174E-06
NOTCH2		0.139	0.001702075
SLC38A1	ATA1	0.167	0.000148386
PLEKHN1	CLPABP	0.009	0.835885261
RBPJ	IGKJRB	0.119	0.007192781
VRK2		0.309	1E-12
TGFB3		0.323	7.42E-14
WDR83	MORG1	-0.043	0.337397376
UBE2T	HSPC150	0.301	4.13E-12

ADGRD1	GPR133	-0.241	3.35E-08
ATG9B	APG9L2	0.119	0.007236289
NFKBIA	IKBA	0.028	0.529792326
NDNF	C4orf31	-0.221	0.00000048
NTRK1	MTC	0.044	0.324792786
AIMP1	EMAP2	0.074	0.093376549
CIRBP	A18HNRNP	-0.314	4.05E-13
CIAO3	NARFL	-0.131	0.002955949
PRKAA1	AMPK1	-0.109	0.01363993
APBA3	MINT3	0.112	0.011376643
CHRDL1	NRLN1	-0.187	2.19008E-05
NCBP2AS2	HIAR	0.173	0.000085932
PHLDA2	BWR1C	0.196	8.1177E-06
PDLIM1	CLIM1	-0.061	0.170888673
PGK1	PGKA	0.341	2.33E-15
PHB2	BAP	0.034	0.445467738
PENK		-0.232	0.000000115
PDCL3	PhLP2A	0.210	1.78936E-06
PDGFA	PDGF1	-0.094	0.034313359
PIK3CB	PIK3C1	0.054	0.224298806
VCAM1		0.379	6.84E-19
PLAU		0.424	1.03E-23
ENDOG		-0.033	0.456150296
NOS1		-0.158	0.000336615
PCK1	PEPCK1	-0.068	0.124204756
MAPK12	ERK6	0.119	0.007311667
MTHFR		-0.369	6.84E-18
RHOA	ARH12	0.125	0.004731571
TRAK1	KIAA1042	-0.284	6.36E-11
MPL	TPOR	-0.219	0.000000617
ZFP36L1	BERG36	0.087	0.048692316
NFE2L2	NRF2	-0.079	0.076228274
PLOD1	LLH	0.241	3.54E-08
TSPAN12	NET2	-0.097	0.02916562
PPARG	NR1C3	0.012	0.787219425
CXCL12	SDF1	0.185	2.75761E-05
PLAT		0.224	0.000000331
STC2		0.285	5.08E-11
ROCK2	KIAA0619	-0.030	0.499377995
SLC29A1	ENT1	-0.066	0.136352456
SCFD1	C14orf163	0.246	1.86E-08
HMOX2	HO2	0.043	0.332882045
CXXC5	HSPC195	-0.111	0.012062973
ARNT2	BHLHE1	-0.084	0.059499044
LMNA	LMN1	-0.055	0.211514025
ITPR1	INSP3R1	-0.163	0.000226308
MAPK8IP1	IB1	-0.072	0.106278239
MAP2K6	MEK6	0.026	0.553838087
CHRNA4	NACRA4	-0.157	0.000383249
HTR2B		0.077	0.082774901
ACVRL1	ACVRLK1	0.024	0.583097412

KCNA5		-0.089	0.045295882
DNMT3A		0.098	0.026993782
ABAT	GABAT	-0.157	0.000369381
ITPR2		-0.042	0.338164122
CDKN1B	KIP1	0.001	0.976566467
CYBA		-0.067	0.131617134
DDAH1	DDAH	-0.192	1.32449E-05
HMOX1	HO	0.143	0.001244679
SLC2A8	GLUT8	-0.034	0.446380598
ALAS2	ALASE	-0.016	0.719358224
ANGPT4	ANG3	-0.015	0.740339061
ASCL2	BHLHA45	0.002	0.955595465
PRKCE	PKCE	-0.188	1.96772E-05
CPEB1	CPEB	0.112	0.011515065
DDIT3	CHOP	0.072	0.105039366
MACIR	C5orf30	0.030	0.505488461
BCL2		0.037	0.405959029
ARNTL	BHLHE5	-0.025	0.576160918
CASR	GPRC2A	-0.211	1.50304E-06
DIO3	ITDI3	0.028	0.527271825
KCNK9	TASK3	0.156	0.00039499
LEP	OB	0.064	0.147838933
OPRD1	OPRD	0.128	0.003855786
BSG	UNQ6505/PRO21383	0.005	0.905155083
CD38		-0.023	0.601495231
CRYAB	CRYA2	0.066	0.138864494
HSP90B1	GRP94	0.095	0.032213128
FLCN	BHD	-0.075	0.092792198
CYGB	STAP	-0.004	0.920694645
MIEF1	MID51	0.180	4.40364E-05
CSNK1D	HCKID	-0.058	0.188481656
ADAM15	MDC15	0.012	0.780899462
BNIP3L	BNIP3A	-0.024	0.590083742
CTNNB1	CTNNB	0.241	3.51E-08
CXCR4		0.043	0.336706699
CYB5A	CYB5	-0.209	2.00798E-06
FN1	FN	0.432	1.52E-24
E2F1	RBBP3	0.196	8.00245E-06
EPHA4	HEK8	-0.142	0.001311224
AQP3		-0.232	0.00000012
ANGPT2		0.288	3.59E-11
CFLAR	CASH	0.106	0.017146032
ANG	RNASE5	-0.284	6.92E-11
PTK2B	FAK2	-0.273	3.71E-10
JUN		-0.088	0.047843549
FZD4		-0.102	0.021116539
GATA6		-0.196	8.52594E-06
BRIP1	BACH1	0.369	7.5E-18
SLC2A4	GLUT4	-0.063	0.154480871
F7		0.022	0.622768423
SLC2A1	GLUT1	0.259	2.82E-09

PKLR	PK1	-0.025	0.57047491
NOX4	RENOX	0.429	3.25E-24
SLC11A2	DCT1	-0.127	0.004177038
PPARA	NR1C1	-0.096	0.030415873
RHOT1	ARHT1	0.013	0.765583361
NFKB1		-0.016	0.718227668
MMP14		0.380	6.04E-19
PML	MYL	0.087	0.050258791
NECAB3	APBA2BP	-0.255	5.48E-09
ITGA2	CD49B	0.170	0.00011478
MMP2	CLG4A	0.310	7.72E-13
KCNK3	TASK	-0.309	9.7E-13
MECP2		-0.154	0.000479743
BACH1		0.172	9.72692E-05
NKX3-1	NKX3.1	0.016	0.725232293
NCOA2	BHLHE75	-0.017	0.707597498
PLOD2		0.405	1.33E-21
ETS1	EWSR2	0.077	0.083214707
PTEN	MMAC1	0.046	0.296894074
NPPC	CNP2	-0.005	0.918971199
ANK1	ANK	-0.060	0.17876729
NPEPPS	PSA	0.228	0.000000195
NR4A2	NOT	-0.200	5.4265E-06
NOS2	NOS2A	0.067	0.133090611
NF1		0.085	0.056495675
NOP53	GLT	-0.226	0.000000251
SIAH2		0.131	0.003131363
SLC9A1	APNH1	-0.173	0.000086682
NPAS1	BHLHE11	-0.318	1.84E-13
TMEM204	C16orf30	0.039	0.377293212
HIGD1B		-0.249	1.18E-08
HIGD2A		-0.133	0.00266515
APOLD1	VERGE	0.128	0.003727968
FBXL14	FBL14	-0.046	0.294595507
HIGD2B	HIGD2BP	-0.039	0.375972293
SIRT4	SIR2L4	-0.159	0.000299185
RTN4	KIAA0886	0.196	8.58867E-06
SFRP1	FRP	-0.021	0.629702033
P2RX2	P2X2	-0.211	1.54549E-06
P2RX3		0.041	0.350411896
MDM4	MDMX	-0.274	3.05E-10
NPAS3	BHLHE12	0.021	0.63053637
SUV39H1	KMT1A	0.215	0.000000924
RYR2		-0.134	0.00250955
SMAD4	DPC4	0.007	0.870955466
PPARD	NR1C2	0.098	0.026599014
SRC	SRC1	-0.034	0.446969041
SLC35C2	C20orf5	-0.107	0.015684091
SLC6A4	HTT	-0.250	1.11E-08
RYR1	RYDR	-0.025	0.573643547
STOX1	C10orf24	-0.004	0.927366837

SUV39H2	KMT1B	0.324	6.78E-14
TH	TYH	0.077	0.084092012
SCAP	KIAA0199	-0.090	0.042253664
SUMO1	SMT3C	0.107	0.015249856
UBQLN1	DA41	0.146	0.000941389
SEMA4B	KIAA1745	0.109	0.013805755
STC1	STC	0.373	2.61E-18
SOD3		-0.080	0.070289883
SOD2		0.115	0.009593203
TREM2		0.064	0.149970088
MDM2		-0.163	0.000212638
TNF	TNFA	0.087	0.050786821
VASN	SLITL2	0.069	0.118762051
USF1	BHLHB11	-0.097	0.028405801
SRF		0.175	7.28643E-05
UCP2	SLC25A8	0.022	0.621003122
NCOA1	BHLHE74	-0.157	0.000362755
PPP1CA	PPP1A	0.041	0.361364775
ELOC	TCEB1	0.247	1.59E-08
FABP1	FABPL	-0.045	0.306408643
UBE2D3	UBC5C	0.063	0.152262565
LONP1	PRSS15	0.109	0.014064011
TGFB2		0.046	0.294984993
TXN2	TRX2	-0.087	0.049546028
RPL8		0.061	0.16833757
PTGIS	CYP8	0.162	0.00023864
ADSL	AMPS	0.310	8.6E-13
KDR	FLK1	0.121	0.006273961
UBE2D2	PUBC1	0.142	0.001289671
XRCC1		-0.203	3.80747E-06
TEK	TIE2	-0.115	0.009425422
TWIST1	BHLHA38	0.458	8.48E-28
VEGFB	VRF	0.066	0.133912142
ACAA2		0.039	0.376642002
PIN1		-0.025	0.575814489
TGFBR3		-0.262	2.01E-09
WDR26	CDW2	-0.127	0.004163319
THBS1	TSP	0.309	9.1E-13
UBE2I	UBC9	0.151	0.000647866
PGF	PGFL	0.282	8.42E-11
LTA	TNFB	0.034	0.438076622
NDUFS2		-0.021	0.638056323
TERT	EST2	0.125	0.004565257
ZNF408	PFM14	-0.007	0.88191047
PLAUR	MO3	0.273	3.44E-10
VEGFC		0.212	1.3791E-06
TBL2	WBSCR13	0.233	0.000000101
UCN3	SPC	-0.091	0.040134356
TGFBR2		-0.111	0.011957338
PTPRB	PTPB	-0.210	1.63498E-06
UCP3	SLC25A9	-0.245	2.13E-08

VEGFD	FIGF	-0.385	1.72E-19
TLR2	TIL4	-0.061	0.165983689
OMA1	MPRP1	-0.241	3.53E-08
TFRC		0.177	5.67372E-05
UBE2D1	SFT	0.286	4.66E-11
CYP1A1		0.043	0.332241585
RHOT2	ARHT2	-0.256	4.79E-09
MT-ND4	MTND4	NA	NA
PFKL		-0.078	0.079509583
PTGS2	COX2	0.102	0.020931615
AGER	RAGE	-0.331	1.79E-14
RGCC	C13orf15	-0.242	3.28E-08
CHRNB2		-0.085	0.056170173
BMP7	OP1	-0.077	0.081236383
ACE	DCP	-0.102	0.021533581
APAF1	KIAA0413	NA	NA
AGTRAP	ATRAP	-0.130	0.003155752
CCNA2	CCN1	0.363	2.72E-17
ATP2A2	ATP2B	0.102	0.021825679
CHRNA7	NACHRA7	0.013	0.767613189
BMP2	BMP2A	-0.174	8.01915E-05
BHLHE40	BHLHB2	0.097	0.029280364
NT5C1A		-0.088	0.047610751
BBC3	PUMA	-0.055	0.214504815
ACTN4		-0.051	0.253161784
ADIPOQ	ACDC	0.066	0.138621792
ATF2	CREB2	0.164	0.000206196
FOXO3	FKHRL1	-0.104	0.018408517
IL1A	IL1F1	0.169	0.000120947
IKBKB	IKKB	-0.128	0.003769382
CPEB2		-0.124	0.004884118
ELOB	TCEB2	-0.070	0.114542769
ALAD		-0.199	6.06506E-06
HSF1	HSTF1	0.088	0.045809447
SLC8A1	CNC	0.085	0.0542434
SLC8A3	NCX3	-0.228	0.000000203
MYC	BHLHE39	0.288	3.6E-11
CYB5R3	DIA1	-0.137	0.001856949
MB		0.172	9.15283E-05
NAA10	ARD1	0.174	7.60799E-05
MYOCD	MYCD	-0.180	4.49035E-05
PPP1R12A	MBS	0.218	0.000000694
MT-ND5	MTND5	NA	NA
KCND2	KIAA1044	0.425	8.29E-24
NDP	EVR2	0.057	0.199770341
KCNMA1	KCNMA	0.027	0.53917327
KCNMB1		0.219	0.00000059
HIGD1C	UBIE	-0.064	0.147479028
BAD	BBC6	-0.075	0.089502473
GNGT1		0.020	0.647302502
IRAK1	IRAK	0.235	0.000000079

MIEF1		0.180	4.40364E-05
ADM	AM	0.166	0.000170697
CEP192	KIAA1569	-0.053	0.233233879
CITED4	MRG2	-0.117	0.008280521
COX4I2	COX4L2	-0.203	3.88552E-06
ADORA1		0.166	0.00016344
PAM		0.029	0.508693365
STUB1	CHIP	-0.094	0.033962286
CD24	CD24A	-0.007	0.877672845
COL4A1		0.222	0.000000402
CLDN3	C7orf1	0.056	0.209157936
CLCA1	CACC1	-0.026	0.555125696
ATM		-0.104	0.019296569
BIRC2	API1	0.163	0.000220206
TMBIM6	BI1	-0.192	1.32225E-05
CA2		-0.101	0.022410058
BECN1	GT197	-0.023	0.609955263
ARNTL2	BHLHE6	0.236	6.73E-08
DEPP1	C10orf10	0.060	0.178933076
CYBB	NOX2	0.145	0.001016533
DAPK1	DAPK	-0.352	2.59E-16
CAT		-0.236	7.32E-08
CAV3		-0.291	2.18E-11
HSD11B2	HSD11K	-0.209	1.88456E-06
EDNRA	ETA	0.270	5.81E-10
ATG16L1	APG16L	0.007	0.87025583
ADAM17	CSVP	0.267	8.47E-10
CD34		-0.077	0.080395448
ATP1B1	ATP1B	-0.297	7.39E-12
ATP7A	MC1	-0.043	0.330189059
CAPN2	CANPL2	-0.111	0.0119205
AQP1	CHIP28	-0.140	0.001578437
PMAIP1	NOXA	0.406	1.23E-21
EDN1		0.013	0.771294634
ERCC3	XPB	-0.037	0.405315109
DRD2		0.035	0.425476689
ADAM8	MS2	0.031	0.480537491
CAV1	CAV	-0.094	0.033016052

Table S4: Correlation of mRNA levels between ALDH1B1 and JAK-STAT pathway-related molecules

Gene name	Secondary gene name	Rho	P-value
GH1		0.033	0.4629882
JAK1	JAK1A	-0.062	0.164332489
JAK2		0.057	0.196254053
SOCS1	SSI1	0.052	0.24448574
IFNAR1	IFNAR	0.025	0.575148341
IFNL1	IL29	0.040	0.36267688
SOCS3	CIS3	0.079	0.073718445
TYK2		-0.102	0.02091476
IFNG		0.119	0.007177195
IGF1R		-0.149	0.000737402
GBP7	GBP4L	0.017	0.693996396
SOCS2	CIS2	-0.085	0.05629467
STAT3	APRF	-0.103	0.019610997
HES1	BHLHB39	0.028	0.533963471
IL10RB	CRFB4	0.118	0.007428104
IL12RB1	IL12R	0.077	0.081601103
CCR2	CMKBR2	0.080	0.070347061
EPOR		-0.236	6.95E-08
JAK3		0.043	0.337075301
IFNL3	IL28B	0.098	0.026937104
IL10		0.130	0.003383059
STAT4		-0.008	0.856963379
STAT5B		-0.065	0.144213896
ADIPOR1	PAQR1	-0.190	1.54078E-05
STAT6		-0.369	6.87E-18
PDCD4	H731	-0.251	9.52E-09
SOCS5	CIS6	0.124	0.004897405
LEPROT	LEPR	0.129	0.003500979
PTPN1	PTP1B	0.003	0.954411966
SNRPA1		0.105	0.017864352
GH2		0.048	0.282989867
SH2B3	LNK	0.103	0.020062406
PTPRD		0.092	0.038005637
PIAS1	DDXBP1	-0.182	3.43353E-05
PRL		0.073	0.099406376
RPLP0		0.152	0.000583994
CSF2	GMCSF	0.120	0.006806238
GSTA2	GST2	-0.155	0.000454194
IL10RA	IL10R	0.063	0.152280135
IL22RA1	IL22R	-0.054	0.222892895
KHDRBS1	SAM68	0.203	3.71368E-06
AIP	XAP2	-0.074	0.097005675
HGS	HRS	-0.028	0.523061711
CFL1	CFL	0.209	1.96507E-06
EPHB2	DRT	0.232	0.000000115
IL12RB2		0.226	0.000000245
HNRNPF	HNRPF	0.161	0.000251317

LMNB1	LMN2	0.289	2.82E-11
IL12A	NKSF1	-0.046	0.301755808
IFNLR1	IL28RA	-0.207	2.55396E-06
DAB2IP	AF9Q34	-0.146	0.000957805
ERBB4	HER4	-0.282	9.08E-11
CRLF3	CREME9	0.151	0.000631793
LYN	JTK8	0.273	3.9E-10
HAMP	HEPC	0.086	0.051355697
MSN		0.101	0.021960894
IFNL2	IL28A	-0.009	0.836810626
SERPINB2	PAI2	0.011	0.797236849
CCL5	D17S136E	0.038	0.392455817
IFI27		0.063	0.153650534
BOLA2	BOLA2A	0.126	0.004404877
IFNGR1		-0.066	0.135159456
CAPZA1		0.220	0.000000505
CBL	CBL2	0.132	0.002815599
DOT1L	KIAA1814	-0.070	0.112647888
MST1	D3F15S2	-0.216	0.000000801
ELP2	STATIP1	0.204	3.46591E-06
BCL3	BCL4	0.078	0.077615662
CSH2		0.109	0.01341103
IFNGR2	IFNGT1	0.124	0.005187402
MTAP	MSAP	0.287	3.67E-11
CYP1B1		0.289	2.87E-11
MST1L	D1F15S1A	-0.236	7.22E-08
CSF2RA	CSF2R	0.085	0.054770908
HNRNPDL	HNRPDL	-0.120	0.006875225
IFNAR2	IFNABR	0.192	1.31726E-05
HSPA9	GRP75	0.041	0.358466462
LEPR	DB	-0.098	0.027438245
CA1		-0.193	1.12142E-05
EGF		0.031	0.478533174
PIGU	CDC91L1	-0.015	0.74164541
PAK2		0.298	6.49E-12
OCIAD1	ASRIJ	-0.082	0.063781222
PIBF1	C13orf24	-0.069	0.118972699
PKD1		-0.150	0.000667404
MIF	GLIF	0.154	0.000474148
TALDO1	TAL	-0.048	0.281815578
RAP1B	OK/SW-cl.11	0.181	3.92991E-05
SOCS6	CIS4	-0.042	0.34148243
STAT1		0.186	2.47791E-05
PIM1		0.223	0.000000374
HNRNPA2B1	HNRPA2B1	0.015	0.734313951
NMI		0.138	0.001770833
F2R	CF2R	0.131	0.00313499
PKD2	TRPP2	0.115	0.009130608
SOD1		-0.026	0.554439699
SH2B2	APS	0.148	0.000794553
STAT5A	STAT5	0.042	0.344374572

PRLR		0.125	0.00453339
STAT2		-0.104	0.018739822
TCP1	CCT1	0.220	0.000000547
TRIM10	RFB30	0.075	0.091773893
PPIA	CYPA	0.211	1.60418E-06
PTPRC	CD45	0.077	0.081588369
F2		-0.040	0.367954862
PITPNA	PITPN	-0.127	0.004024292
LCP1	PLS2	0.148	0.000820929
STAMBP	AMSH	0.120	0.006486048
OCIAD2		0.103	0.020205148
KIDINS220	ARMS	-0.046	0.296273469
RALA	RAL	0.281	1.11E-10
CDC42		0.046	0.304852217
ANXA2	ANX2	0.030	0.496597358
IL23A	SGRF	0.169	0.000122419
IL23R		-0.044	0.326398703
IL26	AK155	-0.028	0.529321225
IL31RA	CRL3	0.118	0.007760847
CSF2RB	IL3RB	0.058	0.187615287
IL5		0.026	0.560841833
IL6ST		-0.036	0.41253732
IL6	IFNB2	0.204	0.000003445
IL9		0.025	0.580224893
IFI27L1	FAM14B	-0.089	0.043834961
LCN2	HNL	-0.094	0.033424431
NEUROD1	BHLHA3	0.061	0.166110356
CSHL1	CSHP1	0.054	0.225693883
GSTO1	GSTTLP28	0.133	0.002524111
KIT	SCFR	-0.148	0.000778153
HES5	BHLHB38	-0.098	0.026737456
IL12B	NKSF2	-0.215	1.00952E-06
IFI27L2	FAM14A	0.016	0.717513105
IFNB1	IFB	0.159	0.00031879
CLCF1	BSF3	0.105	0.017373284
CENPJ	CPAP	0.087	0.049291958
CCL2	MCP1	0.196	8.27405E-06
CNN2		0.071	0.110663381
CNOT4	NOT4	0.114	0.010136717
CISH	G18	-0.103	0.019628443
GHR		-0.044	0.324085766
CTR9	KIAA0155	-0.050	0.264178854
DAB1		0.123	0.005575069
ARF1		-0.078	0.076625026
CSH1		0.019	0.675924516

Table S5: Correlation of mRNA levels between ALDH1B1 and PI3K-PTEN/Akt pathway-related molecules

Gene name	Secondary gene name	Rho	P-value
YES1	YES	0.216	0.000000831
XBP1	TREB5	-0.103	0.019661216
WDR91	HSPC049	-0.052	0.243229099
WDR81		-0.129	0.003579292
VAV1	VAV	0.070	0.116729597
UVRAG		-0.076	0.086587069
TRRAP	PAF400	0.111	0.012200274
TRIM27	RFP	-0.079	0.073994858
TRAT1	TCRIM	0.014	0.756840703
TRAF6	RNF85	-0.031	0.486656871
TNS2	KIAA1075	-0.362	2.78E-17
TNIP1	KIAA0113	-0.012	0.781345386
TNFAIP8L3	TIPE3	0.022	0.627582805
TMEM74		0.232	0.000000115
TLR9	UNQ5798/PRO19605	0.086	0.052459346
TIMP1	CLGI	0.191	1.44232E-05
THEM4	CTMP	-0.094	0.03406794
TGFA		0.150	0.000692166
STYK1	NOK	0.013	0.767843793
STX17		-0.098	0.027058297
STRN		0.175	6.86535E-05
STK11	LKB1	0.032	0.465688235
STEEP1	CXorf56	0.156	0.000414401
SPATA13		-0.133	0.002704005
SMG1	ATX	-0.110	0.012660705
SMARCA2	BAF190B	-0.104	0.018299744
SLAMF7	CS1	0.181	3.82111E-05
SLAMF1	SLAM	0.051	0.251570081
SHTN1	KIAA1598	-0.159	0.000316915
SH3GLB1	KIAA0491	0.167	0.000153027
SH3BP1		0.129	0.003527781
SH2D1B	EAT2	-0.070	0.116368874
S100A9	CAGB	0.095	0.032268264
RUFY3	KIAA0871	-0.296	8.95E-12
RUBCNL	C13orf18	0.139	0.001613009
RUBCN	KIAA0226	0.090	0.042919345
RPS6KA3	ISPK1	NA	NA
RPS6KA1	MAPKAPK1A	-0.240	3.86E-08
RNF157	KIAA1917	0.080	0.072299545
RICTOR	KIAA1999	-0.065	0.142903427
RHOG	ARHG	0.126	0.004453802
REG3A	HIP	-0.039	0.376516994
RB1		0.081	0.06924835
RAPGEF3	CGEF1	-0.371	4.07E-18
RACK1	GNB2L1	-0.022	0.624867534
RAC2		0.097	0.028390232
RAC1	TC25	0.150	0.000669524

RAB39A	RAB39	0.122	0.005681886
RAB31	RAB22B	0.305	2.01E-12
PTPN13	PNP1	-0.144	0.001088845
PTPN11	PTP2C	0.170	0.000118121
PTN	HBNF1	-0.113	0.010777222
PTK2	FAK	-0.018	0.6817361
PTGDR2	CRTH2	-0.297	7.24E-12
PRR5	PROTOR1	0.017	0.697159561
PRR14		-0.068	0.124052112
PRKDC	HYRC	0.268	8.01E-10
PRKCZ	PKC2	-0.329	2.29E-14
PRKCQ	PRKCT	-0.166	0.000171141
PRKCI	DXS1179E	-0.005	0.909701619
PRKCH	PKCL	-0.094	0.032897466
PRKCB	PKCB	-0.052	0.240000682
PREX2	DEPDC2	-0.163	0.00021284
PPP2R5E		0.306	1.74E-12
PPP2R5D		0.165	0.000180622
PPP2R5C	KIAA0044	-0.023	0.607237229
PPP2R5B		0.070	0.113080969
PPP2R5A		-0.232	0.000000111
PPP2R1B		0.045	0.314397105
PPP2R1A		-0.068	0.122842122
PPP2CB		-0.090	0.041742859
PPP2CA		0.148	0.000807226
PPP1R16B	ANKRD4	-0.046	0.295880983
PODXL	PCLP	0.073	0.099427849
PLEKHO1	CKIP1	0.270	5.55E-10
PLEKHH2	KIAA2028	-0.254	6.02E-09
PIP5K1C	KIAA0589	0.004	0.930165455
PIP5K1B	STM7	-0.297	7.81E-12
PIP5K1A		0.090	0.042681134
PIP4K2C	PIP5K2C	0.054	0.224162924
PIP4K2B	PIP5K2B	0.007	0.873938862
PIP4K2A	PI5P4KA	0.251	9.44E-09
PIM2		0.147	0.000857968
PIK3R6	C17orf38	-0.030	0.50503622
PIK3R5		-0.018	0.687478827
PIK3R4	VPS15	0.089	0.044475817
PIK3R3		-0.105	0.018120071
PIK3R2		0.128	0.003814121
PIK3R1	GRB1	-0.065	0.14458974
PIK3IP1	HGFL	-0.224	0.000000335
PIK3CG		0.119	0.006981022
PIK3CD		0.011	0.797290303
PIK3CA		0.163	0.00021134
PIK3C3	VPS34	0.096	0.02990489
PIK3C2G		-0.167	0.00015088
PIK3C2B		-0.282	8.36E-11
PIK3C2A		-0.057	0.197348233
PIK3AP1	BCAP	0.181	3.99805E-05

PI4KB	PIK4CB	-0.058	0.192160396
PI4KAP2		-0.163	0.000220341
PI4KAP1		-0.200	5.5036E-06
PI4KA	PIK4	-0.134	0.002481997
PI4K2B		-0.003	0.937676327
PI4K2A		0.141	0.00145054
PHLPP2	KIAA0931	0.082	0.062848788
PHLPP1	KIAA0606	0.026	0.552816582
PELP1	HMX3	-0.046	0.304249884
PEBP4	CORK1	-0.364	1.93E-17
PDPK1	PDK1	-0.188	1.96494E-05
PDGFRB	PDGFR	0.186	2.42404E-05
PDGFRA	PDGFR2	0.270	5.63E-10
PDGFB	PDGF2	0.075	0.090090548
PDE3B		-0.164	0.00020083
PDCD6	ALG2	0.083	0.060173006
PACS1	KIAA1175	0.031	0.478118699
OPRM1	MOR1	-0.051	0.252177243
OCRL	OCRL1	0.044	0.322361843
OBSCN	KIAA1556	-0.215	0.000000951
NYAP2	KIAA1486	-0.010	0.822771011
NYAP1	C7orf51	-0.055	0.210962562
NTRK3	TRKC	-0.095	0.031540413
NTRK2	TRKB	-0.085	0.054641277
NTF4	NTF5	-0.227	0.000000227
NTF3		0.119	0.00736671
NRG4		-0.224	0.000000316
NRG3		-0.113	0.010968391
NRG2	NTAK	-0.197	7.69076E-06
NRG1	GGF	-0.010	0.814276108
NRBF2	COPR	0.220	0.000000541
NLRC3	NOD3	-0.106	0.016552827
NGF	NGFB	0.134	0.002451199
MYO1F		0.010	0.829892852
MYO16	KIAA0865	-0.156	0.000409686
MYDGF	C19orf10	0.101	0.023110789
MYD88		0.133	0.002613935
MST1R	PTK8	-0.147	0.000845768
MLST8	GBL	-0.017	0.694950418
MET		0.107	0.015432585
MECOM	EVI1	-0.294	1.27E-11
MAPKAP1	MIP1	0.175	7.36819E-05
MAPK3	ERK1	-0.191	1.46599E-05
MAPK1	ERK2	0.013	0.767906843
MAP3K8	COT	-0.092	0.037845139
MAP3K14	NIK	-0.068	0.126252828
LTK	TYK1	-0.015	0.734265596
LCP2		0.147	0.000860418
LCK		0.025	0.580905248
LAMP3	DCLAMP	-0.293	1.37E-11
KLB		-0.166	0.000171634

KL		-0.241	3.51E-08
KITLG	MGF	-0.012	0.791799374
KCNQ1	KCNA8	-0.357	9.16E-17
KANK1	ANKRD15	-0.068	0.126989025
IRS2		-0.161	0.000256821
IRS1		0.177	5.60381E-05
IRAK4		-0.002	0.969828728
IPCEF1	KIAA0403	0.027	0.541475705
INSR		-0.055	0.21807649
INS		-0.035	0.436547219
INPPL1	SHIP2	-0.182	3.52334E-05
INPP5E		-0.160	0.000279189
INPP5D	SHIP	-0.020	0.644292032
INPP4B		0.124	0.005030716
INPP4A		-0.001	0.976218647
ILK	ILK1	0.076	0.087637193
IL33	C9orf26	-0.268	7.22E-10
IL1RL1	DER4	-0.078	0.077443276
IL1RAP	C3orf13	0.204	3.43666E-06
IGF1	IBP1	0.120	0.006880256
IER3	DIF2	0.086	0.053115084
ICOS	AILIM	0.111	0.012362274
HRAS	HRAS1	-0.022	0.621215444
HGF	HPTA	0.035	0.434361425
HDAC3		0.039	0.378411565
HBEGF	DTR	0.043	0.330746821
GRB2	ASH	0.155	0.000442811
GPER1	CEPR	-0.058	0.193769823
GNB1		0.154	0.000479324
GAB2	KIAA0571	-0.130	0.003216331
GAB1		-0.129	0.003533234
FYN		0.106	0.016564713
FSHR	LGR1	-0.046	0.29943742
FRS2		0.025	0.572187772
FLT3LG		-0.022	0.617117517
FLT3	CD135	-0.061	0.170449484
FLT1	FLT	0.207	2.33814E-06
FGFR4	JTK2	-0.272	4.08E-10
FGFR3	JTK4	-0.347	7.49E-16
FGFR2	BEK	-0.215	0.000000941
FGFR1	BFGFR	0.249	1.24E-08
FGF9		-0.040	0.371257933
FGF8	AIGF	0.045	0.314561183
FGF7	KGF	0.186	2.47402E-05
FGF6	HST2	0.003	0.947068098
FGF5		0.371	4.7E-18
FGF4	HST	-0.068	0.126976851
FGF3	INT2	0.170	0.000112994
FGF23	HYPF	0.177	5.65416E-05
FGF22	UNQ2500/PRO5800	-0.117	0.008137457
FGF20		-0.075	0.090196432

FGF2	FGFB	-0.060	0.17485437
FGF19	UNQ334/PRO533	0.016	0.711082898
FGF18	UNQ420/PRO856	-0.058	0.19223609
FGF17	UNQ161/PRO187	-0.184	2.83659E-05
FGF16		0.143	0.001221777
FGF10		0.027	0.5460257
FGF1	FGFA	0.287	4.13E-11
FBXO9	FBX9	-0.149	0.000738558
FBXL2	FBL2	0.047	0.286943547
FAM83B	C6orf143	0.037	0.404665418
FAM83A	TSGP	0.155	0.000436943
FAM168A	KIAA0280	0.217	0.000000716
ESR2	ESTRB	-0.075	0.089202366
EREG		0.162	0.000237972
ERBB3	HER3	-0.317	2.46E-13
ERBB2	HER2	-0.158	0.000337843
EPGN	UNQ3072/PRO9904	-0.001	0.977127345
ENTPD5	CD39L4	0.019	0.670158123
EIF4EBP2		-0.152	0.00057864
EIF4B		-0.082	0.063814221
EGFR	ERBB	0.033	0.455313652
EEA1	ZFYVE2	0.041	0.360969909
E2F4		0.105	0.017868215
DHRS7C	SDR32C2	0.010	0.815836016
DAPP1	BAM32	0.063	0.156372754
CRNN	C1orf10	0.002	0.971219789
CMKLR1	CHEMR23	0.084	0.057164664
CEP350	CAP350	-0.015	0.733310862
CEBPA	CEBP	-0.225	0.000000293
CDK4		0.345	1.1E-15
CDK2	CDKN2	0.265	1.27E-09
CD86	CD28LG2	0.200	5.52158E-06
CD80	CD28LG	0.203	3.98687E-06
CD33	SIGLEC3	0.063	0.157331208
CD28		0.082	0.063883007
CD244	2B4	0.013	0.76257748
CD19		-0.019	0.670133543
CCDC88C	DAPLE	-0.144	0.001135764
CCDC88A	APE	0.211	1.4574E-06
CC2D1A	AKI1	-0.104	0.019377384
CBLB	RNF56	0.052	0.237042146
C1QTNF12	C1QDC2	-0.070	0.114778351
C1QBP	GC1QBP	0.131	0.003108665
BTC		-0.044	0.324105674
BDNF		-0.141	0.001368117
BCAR3	NSP2	0.055	0.214716096
BCAR1	CAS	-0.063	0.158035062
BACH2		0.159	0.000313154
ATG14	ATG14L	-0.079	0.075692749
ARHGEF5	TIM	-0.074	0.096902325
AREG	AREGB	0.068	0.124527863

ARAP3	CENTD3	-0.209	1.93321E-06
APPL2	DIP13B	0.018	0.691216135
APPL1	APPL	0.115	0.009395818
AKT3	PKBG	0.250	1.07E-08
AKT2		0.035	0.427409709
AKT1	PKB	-0.114	0.010206626

Table S6: Correlation of mRNA levels between ALDH1B1 and retinoic acid pathway-related molecules

Gene name	Secondary gene name	Rho	P-value
CYP26A1	CYP26	0.322	9.44E-14
CRABP2		0.214	1.09159E-06
CYP26B1	CYP26A2	0.232	0.000000118
DDX58		0.218	0.000000628
GPRC5A	GPCR5A	-0.262	1.85E-09
NCOR2	CTG26	-0.002	0.972311684
RARA	NR1B1	-0.054	0.226748632
RXRA	NR2B1	-0.224	0.000000337
RARB	HAP	0.074	0.096737169
RARG	NR1B3	-0.058	0.191178226
RXRG	NR2B3	-0.267	8.4E-10
RXRB	NR2B2	-0.201	4.57208E-06
STRA6	PP14296	0.192	0.000012657
GPRC5B	RAIG2	0.140	0.001547955
ATRAID		-0.071	0.110129378
BRINP1	DBC1	0.124	0.004940055
LY6E	9804	-0.017	0.707978032
SKAP2	PRAP	0.246	1.72E-08
RAET1E	LETAL	0.041	0.354622765
RAI14	KIAA1334	0.169	0.000122318
NAV2	HELAD1	0.098	0.027238397
APBB1IP	PREL1	-0.053	0.234955049
GPRC5C	RAIG3	-0.337	5.42E-15
PLAAT4	RARRES3	-0.013	0.768474419
LAPTM5	KIAA0085	0.157	0.000379477
RARRES2	TIG2	0.267	9.18E-10
KDM1A	AOF2	0.109	0.013687779
ACAN	AGC1	0.266	1.06E-09
SERPINF1	PEDF	0.290	2.25E-11
PROX1		0.101	0.022657565
PTK7	CCK4	0.115	0.009493344
PAX2		-0.057	0.202678415
PDHA2	PDHAL	0.062	0.162804204
OGT		-0.115	0.009399197
SLC10A3	DXS253E	0.105	0.017591017
RAB24		-0.154	0.000476672
PDK4	PDHK4	-0.226	0.000000255
RDH16	RODH4	-0.089	0.044706933
RDH5	HSD17B9	-0.159	0.00031388

CRABP1	RBP5	0.098	0.026716527
RBP4	PRO2222	-0.049	0.26654073
SYNJ1	KIAA0910	-0.073	0.098719626
TACC1	KIAA1103	-0.134	0.002414199
MED25	ACID1	-0.218	0.000000656
SMARCA4	BAF190A	0.102	0.021349543
SNW1	SKIIP	0.186	0.000023246
SLURP1	ARS	0.074	0.097001027
TSPYL2	CDA1	-0.256	4.32E-09
PRAM1		-0.043	0.338090033
RORB	NR1F2	-0.123	0.005301731
RARRES1	PEIG1	0.116	0.008769663
RMDN3	FAM82A2	-0.215	0.00000091
TESC	CHP3	-0.202	4.06104E-06
CENPX	FAAP10	0.044	0.317411659
CYP2C8		-0.200	5.05317E-06
CYP3A4	CYP3A3	-0.160	0.000282919
GREB1L	C18orf6	0.202	4.17766E-06
LRAT		-0.150	0.00069044
LIN28A	CSDD1	0.018	0.691262199
MAVS	IPS1	-0.190	1.59924E-05
ASCL1	ASH1	-0.026	0.562393918
ADH1C	ADH3	-0.219	0.000000601
IGF2R	MPRI	0.112	0.011362043
HMGA1	HMGIY	0.278	1.59E-10
EPHA3	ETK	-0.004	0.926348347
FZD10		-0.010	0.821999985
KDM4D	JHDM3D	0.060	0.178311474
FOLR2		0.024	0.584958445
GDAP1		-0.059	0.182245974
HDAC2		0.265	1.22E-09
CYP26C1		0.172	9.58602E-05
KMT2E	MLL5	-0.090	0.04184529
TFAP2A	AP2TF	0.077	0.082719786
NOP2	NOL1	0.138	0.001760625
NPM3		0.188	0.000019765
DCD	AIDD	-0.056	0.205307728
EZH2	KMT6	0.244	2.24E-08
HTRA2	OMI	0.248	1.44E-08
MEIOC	C17orf104	0.185	2.71014E-05
OXT	OT	0.069	0.11774552
CBX3		0.252	8.27E-09
GSK3B		0.069	0.119291993
IRF1		0.060	0.177065867
JAML	AMICA1	-0.066	0.133690251
IFIT3	CIG-49	0.120	0.006478936
NCOR1	KIAA1047	-0.125	0.004700605
NR2C2	TAK1	-0.169	0.00013156
PDHX	PDX1	0.071	0.11124625
FABP4		-0.038	0.392639807
NR1I3	CAR	0.109	0.013749729

GJB2		0.311	6.35E-13
GJB3	CX31	0.074	0.097095464
CBX5	HP1A	-0.009	0.835800907
IFIH1	MDA5	0.143	0.001187761
CTBP2		0.079	0.075539218
DLD	GCSL	0.190	1.55652E-05
DHRS9	RDH15	-0.026	0.560816561
NCF1	NOXO2	0.079	0.074001819
PRMT3	HRMT1L3	0.242	2.92E-08
CCDC26	RAM	NA	NA
CALHM1	FAM26C	0.043	0.329231682
CYP2C18		-0.230	0.000000151
ESRRG	ERR3	-0.075	0.088557743
MICB	PERB11.2	0.191	1.34132E-05
KLF4	EZF	-0.199	6.07414E-06
AKR1C3	DDH1	-0.151	0.00062377
CDK5	CDKN5	0.133	0.002599177
HSD17B2	EDH17B2	-0.213	1.25443E-06
DPYSL3	CRMP4	0.333	1.13E-14
GDAP2		0.014	0.755691568
NR2F2	ARP1	-0.073	0.098829759
CYP1A2		-0.111	0.012431069
CRKL		0.144	0.001114095
FZD7		0.178	0.000050476
BRD8	SMAP	-0.269	6.53E-10
ELF3	ERT	-0.142	0.001338264
CTSH	CPSB	-0.265	1.13E-09
CALR	CRTC	0.130	0.003324419
CYP3A5		-0.250	1.05E-08
NR1H3	LXRA	-0.100	0.024286323
MDK	MK1	0.112	0.011584701
NDUFAF4	C6orf66	0.146	0.00097753
PPP1CC		0.321	1.04E-13
PDHB	PHE1B	0.033	0.460434458
BRINP3	DBCCR1L	-0.018	0.69050415
MCL1	BCL2L3	-0.015	0.731592009
NREP	C5orf13	0.177	5.98939E-05
DLAT	DLTA	0.155	0.000437162
NR4A1	GFRP1	-0.221	0.000000439
FABP5		-0.051	0.254105596
MUC5B	MUC5	-0.212	1.33219E-06
OLFM4	GW112	0.031	0.491122253
SERPINA5	PCI	0.096	0.030994286
KLF15	KKLF	-0.369	6.72E-18
TRIM71	LIN41	-0.281	1.02E-10
P2RY11		-0.013	0.77043885
DMRT1	DMT1	0.214	1.11349E-06
CYP2W1		-0.096	0.030889715
BTBD11		-0.011	0.797980334
NDUFA13	GRIM19	0.025	0.568766898
NR1H2	LXRB	-0.139	0.001705657

PIAS2	PIASX	0.195	9.29406E-06
NR2E1	TLX	0.068	0.124567345
CYP4F2		0.005	0.913007449
LCN12		-0.313	4.91E-13
OSR1	ODD	-0.259	2.92E-09
RNF135	L13	-0.060	0.177498957
ARIH2	ARI2	-0.013	0.762370057
CYP2D6	CYP2DL1	-0.085	0.056127411
NRIP1		0.356	1.04E-16
MEIOSIN	BHMG1	NA	NA
TMEM161A	UNQ582/PRO1152	0.021	0.637936935
NR1H4	BAR	-0.182	0.000037197
PDHA1	PHE1A	-0.029	0.512808786
SCPEP1	RISC	-0.030	0.502151926
PARG		0.072	0.105301171
PDK2	PDHK2	-0.289	2.96E-11
PTK6	BRK	-0.244	2.54E-08
PCGF1	NSPC1	-0.002	0.957478879
SETX	ALS4	-0.063	0.154959658
SHOX	PHOG	-0.027	0.54176748
RETSAT	PPSIG	-0.214	1.07005E-06
SREBF1	BHLHD1	-0.150	0.000701982
RORC	NR1F3	-0.207	2.32865E-06
POLR3GL		-0.171	0.000109086
OVCA2		0.041	0.355422344
TGIF1	TGIF	0.056	0.202928706
SP100		-0.050	0.260519477
SEMA6B	SEMAN	0.165	0.000189485
SP110		-0.039	0.379892787
STRA8		0.032	0.473788504
UGT1A8	GNT1	-0.050	0.255291034
SLA	SLAP	0.090	0.042987151
UGT1A1	GNT1	0.024	0.584589564
PPAN	BXDC3	0.127	0.004013088
TAF4	TAF2C	0.030	0.493028971
TGM2		0.061	0.165708356
UGT1A9	GNT1	0.020	0.650202959
SMYD5	RAI15	0.194	1.07333E-05
TRIM24	RNF82	0.007	0.869983063
IFIT5	ISG58	0.076	0.08814805
NSD1	ARA267	-0.089	0.045541058
TBX1		-0.009	0.838986196
TRIM16	EBBP	0.019	0.673011028
TRAF3	CAP1	0.087	0.049376289
PRAME	MAPE	0.116	0.009003123
ULBP2	N2DL2	0.154	0.000466582
WNT3A		-0.191	1.38336E-05
WNT7B		0.018	0.676919631
PADI4	PAD4	-0.021	0.635604881
WNT8A	WNT8D	-0.052	0.245043991
ZGLP1	GLP1	-0.102	0.021509888

UGT1A3	GNT1	0.039	0.382842511
TXNRD1	GRIM12	0.175	6.78037E-05
WNT3	INT4	0.018	0.692561846
LRIF1	C1orf103	0.222	0.000000422
ZNF304		0.007	0.868329944
TWF2	PTK9L	0.152	0.000570056
RAET1G	ULBP5	-0.063	0.152597713
UBE2L6	UBCH8	0.174	8.00483E-05
UGT1A7	GNT1	-0.019	0.670753694
UGT2B7	UGTB2B9	-0.070	0.112998819
TRIM14	KIAA0129	0.057	0.202697622
NR2C1	TR2	-0.121	0.006214318
RAET1L	ULBP6	0.097	0.028066528
ULBP1	N2DL1	0.191	1.35416E-05
WNT5A		0.053	0.229796932
ZNF609	KIAA0295	-0.112	0.011742578
ZFP42	REX1	-0.042	0.347426365
WNT5B		0.180	4.28073E-05
WNT11		-0.235	8.22E-08
WNT2	INT1L1	0.282	9.11E-11
WNT9B	WNT14B	0.006	0.887088608
ZMIZ1	KIAA1224	-0.120	0.006548434
RBP1	CRBP1	0.179	5.03783E-05
RBP7		0.071	0.108020815
TBX2		-0.203	3.98038E-06
TIE1	TIE	-0.052	0.238827033
TMSB10	PTMB10	0.110	0.013170728
ULK4		-0.080	0.070621724
RET	CDHF12	-0.005	0.918678841
WNT9A	WNT14	0.121	0.006367808
WNT8B		-0.030	0.501403908
ZNF536	KIAA0390	-0.175	7.42336E-05
ZBTB16	PLZF	-0.259	2.76E-09
ZNF35		0.118	0.007737989
STK38	NDR1	-0.036	0.421456713
WNT6		0.122	0.005847539
WNT10B	WNT12	-0.031	0.477872611
ULBP3	N2DL3	0.110	0.01281062
ZNF24	KOX17	-0.092	0.038506641
PTF1A	BHLHA29	0.036	0.415588676
PTGDS	PDS	-0.175	6.78626E-05
TBK1	NAK	0.100	0.02342874
PPP1CB		0.172	9.32753E-05
RDH10	SDR16C4	-0.014	0.75214287
RAF1	RAF	-0.038	0.387782912
RBBP5	RBQ3	0.107	0.016072232
RAI1	KIAA1820	-0.121	0.006357213
RAI2		-0.108	0.015132155
ABL2	ABLL	0.202	4.36138E-06
AKAP8	AKAP95	-0.224	0.000000325
ADH4		-0.099	0.025502811

TFAP2C		-0.019	0.675332197
HSPD1	HSP60	0.264	1.43E-09
DHRS3	RDH17	-0.241	3.35E-08
NR2F1	EAR3	-0.077	0.084120924
CYP2S1	UNQ891/PRO1906	-0.080	0.071826222
ASMT		-0.074	0.094568083
ASXL1	KIAA0978	-0.029	0.515585915
ARRDC3	KIAA1376	-0.015	0.728898707
ACER2	ASAH3L	-0.050	0.2574323
ADH1A	ADH1	-0.283	8.03E-11
ALDH1A2	RALDH2	0.078	0.079634645
ADNP2	KIAA0863	0.255	5.05E-09
APOM	G3A	-0.316	2.76E-13
CBX1	CBX	0.271	4.87E-10
IGFBP2	BP2	-0.041	0.349826696
IGFBP7	MAC25	0.085	0.055758133
HOXC10	HOX3I	0.030	0.496452917
HOXC5	HOX3D	0.022	0.627147204
HOXC8	HOX3A	0.133	0.002634856
IFI16	IFNGIP1	0.276	2.2E-10
HOXA1	HOX1F	0.311	6.4E-13
HOXA2	HOX1K	0.094	0.03328122
CPT1A	CPT1	-0.158	0.000351665
MZF1	MZF	-0.280	1.16E-10
MYADM	UNQ553/PRO1110	0.149	0.00073364
MYBL2	BMYB	0.363	2.3E-17
KLF2	LKLF	-0.211	1.51605E-06
LGALS7	PIG1;	-0.013	0.777025079
NPM1	NPM	0.142	0.001345758
NGRN	FI58G	-0.125	0.004852237
KRT7	SCL	-0.084	0.059090735
HCFC1	HCF1	0.014	0.759977901
DNAAF2	C14orf104	0.153	0.000526266
CDKN2D		0.169	0.000121827
CREB3	LZIP	0.224	0.000000328
CAMK1D	CAMKID	0.039	0.376812726
BPIFA1	LUNX	0.023	0.603023611
DTL	CDT2	0.315	3.51E-13
COL1A1		0.454	2.4E-27
CYP3A7		-0.223	0.000000364
ABCA1	ABC1	0.104	0.018411573
BCO2	BCDO2	-0.165	0.000176728
B3GNT5		0.250	9.84E-09
ALDH1A1	ALDC	-0.198	6.91788E-06
ADH7		-0.064	0.146626632
ITGB3BP	CENPR	0.114	0.010202809
CNTN4		-0.076	0.087703478
CNOT1	CDC39	-0.004	0.934505978
GFRA1	GDNFRA	-0.220	0.000000519
GCNT3		-0.135	0.002235788
BRINP2	DBCCR1L2	0.046	0.300325595

ALDH1A3	ALDH6	0.242	2.95E-08
AGO3	EIF2C3	0.148	0.000773664
ADH6		-0.097	0.028758287
ARID5B	DESRT	0.065	0.14284626
CACYBP	S100A6BP	0.215	0.000000906
FHIP2B	FAM160B2	-0.202	4.40677E-06
DEAF1	SPN	-0.023	0.597876636
CYP27C1		0.073	0.097645484
DKK1	UNQ492/PRO1008	-0.051	0.251472779
DPY30		0.043	0.334411603
AHR	BHLHE76	0.009	0.83096323
BABAM2	BRCC45	-0.084	0.057913534
ADH1B	ADH2	-0.320	1.43E-13
ALDH8A1	ALDH12	0.017	0.708869951
ASB2		0.014	0.75627732
ACTB		0.317	2.36E-13
PRMT2	HMT1	-0.171	9.98976E-05
CPT1B	KIAA1670	-0.210	1.72989E-06

Table S7: Correlation of mRNA levels between ALDH1B1 and Wnt pathway-related molecules

Gene name	Secondary gene name	Rho	P-value
WNT9B	WNT14B	0.006	0.887088608
WNT10B	WNT12	-0.031	0.477872611
WNT2B	WNT13	-0.098	0.027338195
WNT8A	WNT8D	-0.052	0.245043991
WNT9A	WNT14	0.121	0.006367808
WNT3A		-0.191	1.38336E-05
WNT5A		0.053	0.229796932
WNT10A		0.084	0.057725626
WNT1	INT1	-0.163	0.000220798
WNT7A		-0.038	0.397944632
WNT3	INT4	0.018	0.692561846
WNT4	UNQ426/PRO864	-0.310	8.11E-13
WNT7B		0.018	0.676919631
WNT16		0.046	0.304948128
WNT6		0.122	0.005847539
WNT2	INT1L1	0.282	9.11E-11
WNT11		-0.235	8.22E-08
WNT5B		0.180	4.28073E-05
CCN4	WISP1	0.453	3.48E-27
WNT8B		-0.030	0.501403908
TPBG	5T4	0.189	1.68441E-05
WIF1	UNQ191/PRO217	-0.339	3.24E-15
RHOU	ARHU	-0.181	3.92615E-05
CCN6	WISP3	0.004	0.936331088
CCN5	CT58	0.103	0.019816023
LINS1	LINS	-0.187	2.13766E-05

CDH3	CDHP	0.233	9.77E-08
LEF1		0.270	5.31E-10
KRTAP5-8	KAP5.8	-0.138	0.00173811
PHACTR3	C20orf101	-0.090	0.041633002
PSENEN	PEN2	-0.022	0.614821087
CBFB		0.093	0.036203632
PTK7	CCK4	0.115	0.009493344
SPINDOC	C11orf84	0.243	2.82E-08
STMN1	C1orf215	0.222	0.00000039
ATP6V1C2		-0.085	0.054661402
ATP6V0D1	ATP6D	-0.137	0.001896235
PBXIP1	HPIP	-0.264	1.36E-09
PRICKLE2		-0.017	0.69693642
RBMS3		-0.002	0.958423922
RAB5A	RAB5	-0.011	0.797822168
RASSF10		-0.125	0.004669059
RBX1	RNF75	0.036	0.417621393
RAC1	TC25	0.150	0.000669524
RAC3		0.171	0.000108373
RNF146		-0.077	0.08323948
RHOA	ARH12	0.125	0.004731571
SHH		-0.370	5.68E-18
SMARCA4	BAF190A	0.102	0.021349543
SOX30		0.126	0.004328175
SENP2	KIAA1331	0.135	0.002302664
SMAD3	MADH3	-0.103	0.020160058
FRZB	FIZ	-0.100	0.023603663
TMED5	CGI-100	0.090	0.042496764
TMEM9	DERP4	-0.082	0.065647112
TIAM1		0.062	0.161187498
TRABD2A	C2orf89	0.033	0.463481262
TRABD2B	HKAT	NA	NA
RSPO1		-0.100	0.024633076
RSPO2	UNQ9384/PRO34209	-0.324	6.42E-14
RSPO3	PWTSR	0.225	0.000000267
RSPO4	C20orf182	-0.159	0.000302612
RUVBL1	INO80H	0.260	2.57E-09
RUVBL2	INO80J	0.167	0.000152825
RTF1	KIAA0252	-0.071	0.107176576
RUNX1	AML1	0.144	0.001123565
NONO	NRB54	0.163	0.000218758
SCEL		-0.157	0.000357725
TPBGL		NA	NA
SKP1	EMC19	-0.006	0.899141967
TSPAN12	NET2	-0.097	0.02916562
UBA52	UBCEP2	0.093	0.035953009
ROR2	NTRKR2	0.353	2E-16
SHISA2	C13orf13	0.030	0.502050598
SHISA6		-0.078	0.077754014
TPI1	TPI	0.284	6.02E-11
TNIK	KIAA0551	-0.033	0.453908439

TMEM198		-0.078	0.080168292
TNKS	PARP5A	-0.116	0.009023578
TNKS2	PARP5B	-0.046	0.298446077
SFRP2	FRP2	0.444	4.16E-26
SPEF1	C20orf28	-0.222	0.000000435
PIAS1	DDXBP1	-0.182	3.43353E-05
PLEKHA4	PEPP1	0.062	0.159755594
SUDS3	SAP45	-0.011	0.806894582
TTC21B	IFT139	-0.084	0.0572582
SRRT	ARS2	-0.023	0.605203345
RPS12		-0.021	0.643656886
SALL1	SAL1	0.182	3.44404E-05
CITED1	MSG1	-0.042	0.346758668
CSNK2A2	CK2A2	0.079	0.073130733
HIC1	ZBTB29	0.171	0.000100236
CCDC88A	APE	0.211	1.4574E-06
LRP4	KIAA0816	0.002	0.963292926
LRP5	LR3	-0.214	1.07819E-06
LRP6		-0.117	0.00803382
LGR4	GPR48	-0.052	0.237733289
LRRFIP2		-0.033	0.456591776
LRRK1	KIAA1790	0.025	0.572623349
LGR5	GPR49	0.106	0.016178247
LRRK2	PARK8	-0.274	2.95E-10
LGR6	UNQ6427/PRO21331	-0.138	0.001786986
MDFIC		0.217	0.000000771
MDFI		0.141	0.001402025
CXXC5	HSPC195	-0.111	0.012062973
HMGB2	HMG2	0.285	5.17E-11
LRP1	A2MR	0.074	0.096232842
MARK1	KIAA1477	-0.034	0.437125373
MARK2	EMK1	-0.042	0.348594958
MARK3	CTAK1	-0.075	0.090638979
MAP4		0.220	0.000000551
MAP3K1	MAPKKK1	-0.088	0.047676272
AP2A2	ADTAB	-0.207	2.51952E-06
DACT3	RRR1	0.160	0.000295656
FERMT2	KIND2	0.278	1.6E-10
GNAI1		-0.149	0.000741411
HMGXB4	HMG2L1	0.292	1.63E-11
JRK	JH8	-0.086	0.052878929
APCDD1	DRAPC1	0.017	0.701461599
APC2	APCL	0.072	0.106111106
APCDD1L		0.476	2.96E-30
JADE1	KIAA1807	-0.208	2.07405E-06
KANK1	ANKRD15	-0.068	0.126989025
PRKCB	PKCB	-0.052	0.240000682
CSNK2B	CK2N	0.058	0.187448029
LARP1	KIAA0731	0.131	0.003062547
DVL1		-0.148	0.000804817
GSK3A		0.035	0.426789305

FZD10		-0.010	0.821999985
FRAT2		-0.192	1.28893E-05
RBCK1	C20orf18	-0.165	0.000189213
KRTAP5-7	KAP5-7	-0.166	0.000172681
BLZF1	JEM1	0.136	0.002045112
FRMD8P1	FKSG43	NA	NA
HMGB3	HMG2A	0.052	0.238360006
IRF2BPL	C14orf4	0.106	0.016359658
APH1A	PSF	0.093	0.035856611
CXXC4	IDAX	-0.128	0.00380277
EDNRB	ETRB	-0.240	4.06E-08
CYBA		-0.067	0.131617134
CCDC88C	DAPLE	-0.144	0.001135764
DAB2IP	AF9Q34	-0.146	0.000957805
GNAI3		0.327	3.87E-14
GPC3	OCI5	-0.162	0.000247942
KRTAP1-5	KAP1.5	0.120	0.006775265
HHEX	HEX	-0.016	0.710854792
APC	DP2.5	-0.110	0.012906949
APOE		0.021	0.633290441
CELA1	ELA1	-0.042	0.344074901
KPNA1	RCH2	0.184	2.96701E-05
LEO1	RDL	-0.007	0.881378177
MUC1	PUM	-0.395	1.75E-20
DDIT3	CHOP	0.072	0.105039366
LBX2	LP3727	-0.003	0.943798513
AP2M1	CLAPM1	0.231	0.000000134
ARNTL	BHLHE5	-0.025	0.576160918
GSK3B		0.069	0.119291993
GNAQ	GAQ	-0.160	0.000293837
GPRC5B	RAIG2	0.140	0.001547955
MACF1	ABP620	-0.129	0.003503507
HDAC1	RPD3L1	-0.091	0.041011538
ILK	ILK1	0.076	0.087637193
FERMT1	C20orf42	0.109	0.013356913
GRB10	GRBIR	0.210	1.71865E-06
NOXA1	P51NOX	-0.320	1.29E-13
NOTCH1	TAN1	-0.063	0.157203737
EIF3D	EIF3S7	0.106	0.016150747
FRAT1		-0.378	9.56E-19
FOXK1	MNF	0.089	0.043611904
MAPK8	JNK1	0.212	1.35884E-06
ARL6	BBS3	0.109	0.013702119
AP2A1	ADTAA	0.164	0.000191408
ARMCX3	ALEX3	0.010	0.822584319
ATP9A	ATPIIA	-0.040	0.366117717
BCL9L	DLNB11	0.039	0.378181336
CTNND1	KIAA0384	-0.151	0.00060462
LATS2	KPM	0.017	0.697627035
KRTAP10-9	KAP10.9	-0.007	0.876419857
DEPDC1B	XTP8	0.337	4.9E-15

LIG1		0.055	0.216347968
DNAJC28	C21orf55	-0.231	0.000000128
DISC1	KIAA0457	-0.090	0.041493781
CTDNEP1	DULLARD	-0.014	0.752238796
DIXDC1	CCD1	0.043	0.33302165
EGR1	KROX24	-0.052	0.240416781
FAM53B	KIAA0140	-0.199	5.78737E-06
FLCN	BHD	-0.075	0.092792198
ISL1		0.045	0.311341728
BRI3		0.026	0.560680573
CTBP1	CTBP	-0.140	0.001565045
CTBP2		0.079	0.075539218
DCDC2	KIAA1154	-0.221	0.000000489
KRTAP5-9	KAP5.9	-0.238	5.21E-08
RPS6KA1	MAPKAPK1A	-0.240	3.86E-08
DACT1	DPR1	0.336	6.5E-15
FAF2	ETEA	-0.033	0.463012466
HESX1	HANF	-0.059	0.181161343
FGF9		-0.040	0.371257933
KRTAP9-3	KAP9.3	NA	NA
TMPO	LAP2	0.223	0.000000354
MKS1		-0.106	0.016497394
ITGA3	MSK18	0.070	0.113271796
CSNK1A1L		0.115	0.009545358
CSNK1A1		0.021	0.629031712
CSNK1D	HCKID	-0.058	0.188481656
CSNK1E		-0.034	0.439306595
CSNK1G1		-0.013	0.767809541
CSNK1G3		0.074	0.095739365
KLF4	EZF	-0.199	6.07414E-06
KLHL12	C3IP1	0.012	0.784932577
AMOTL1		-0.014	0.756503601
CDK5	CDKN5	0.133	0.002599177
CTNNB1	CTNNB	0.241	3.51E-08
MCC		-0.053	0.234915613
CAPRIN2	C1QDC1	-0.149	0.000713984
CBY1	ARB1	0.013	0.769986986
FBXW11	BTRCP2	-0.038	0.38928697
FGFR2	BEK	-0.215	0.000000941
FZD1		0.113	0.010890837
GLUL	GLNS	-0.161	0.000258267
ESR1	ESR	0.152	0.000570639
FGF10		0.027	0.5460257
CDK14	KIAA0834	0.201	4.99331E-06
DCXR	SDR20C1	-0.193	1.11723E-05
EXT1		0.186	0.000024833
EPM2A		-0.114	0.010110628
FOXE1	FKHL15	0.094	0.034517685
FOXO1	FKHR	-0.152	0.000567899
ANKRD6	KIAA0957	-0.076	0.084637739
GPC4	UNQ474/PRO937	-0.069	0.121634449

G3BP1	G3BP	0.295	1.03E-11
FOXL1	FKHL11	0.019	0.663007455
GRK6	GPRK6	0.023	0.608002881
GSKIP	C14orf129	0.210	1.6287E-06
KNOP1	C16orf88	0.270	5.5E-10
JUN		-0.088	0.047843549
FZD2		0.215	0.000000919
FUCA2	PSEC0151	0.129	0.003525097
FZD3		-0.030	0.500663743
FZD4		-0.102	0.021116539
FUZ	FY	-0.104	0.019315844
FZD5	C2orf31	-0.212	1.39859E-06
FZD6		0.132	0.002716257
FZD7		0.178	0.000050476
FZD8		-0.013	0.776478565
FZD9	FZD3	0.089	0.044016588
GPR137	C11orf4	-0.174	7.70157E-05
GATA3		0.033	0.455299479
FRMD8	FKSG44	0.069	0.11710344
CASC3	MLN51	0.067	0.128405128
CASP6	MCH2	0.110	0.012572017
DHX15	DBP1	0.104	0.018673341
ESRRB	ERRB2	-0.058	0.187201181
GREM1	CKTSF1B1	0.545	8.79E-41
ZBTB33	KAISO	0.152	0.000552776
CPE		0.087	0.04978284
CALCOCO1	KIAA1536	-0.367	9.48E-18
EGFR	ERBB	0.033	0.455313652
MDK	MK1	0.112	0.011584701
PRICKLE1	RILP	0.071	0.109067914
PRICKLE3	LMO6	0.137	0.001934801
C1QBP	GC1QBP	0.131	0.003108665
INVS	INV	0.101	0.022910307
NFKB1		-0.016	0.718227668
KRTAP1-1	B2A	0.144	0.001066414
HNRNPC	HNRPC	0.219	0.000000579
MMP14		0.380	6.04E-19
PAF1	PD2	-0.234	8.52E-08
PSMD14	POH1	0.216	0.000000842
PIAS4	PIASG	0.084	0.056694487
KLF15	KKLF	-0.369	6.72E-18
PPM1B	PP2CB	-0.084	0.059396662
KRTAP10-7	KAP10.7	-0.078	0.079416595
KRTAP12-3	KAP12.3	0.024	0.583654695
NOX1	MOX1	-0.051	0.252692131
NOXO1	P41NOX	-0.149	0.000718156
OPRM1	MOR1	-0.051	0.252177243
PLA2G10		-0.396	1.31E-20
GPC6	UNQ369/PRO705	0.469	2.7E-29
MFRP		0.177	5.57314E-05
GSC		0.182	3.61964E-05

EP300	P300	-0.039	0.383230831
PRKCG	PKCG	0.059	0.183646167
DAPK3	ZIPK	0.178	5.45486E-05
DDB1	XAP1	0.089	0.043999772
EGF		0.031	0.478533174
ETV2	ER71	-0.098	0.026782491
CUL1		0.133	0.002715852
CUSTOS	C12orf43	NA	NA
AXIN2		-0.174	7.64692E-05
CCAR2	DBC1	-0.155	0.00042601
FGFR3	JTK4	-0.347	7.49E-16
GPC5		-0.201	4.82056E-06
NLK	LAK1	0.048	0.277323789
CYLD	CYLD1	-0.035	0.433447158
DAAM2	KIAA0381	-0.057	0.195015029
FOXK2	ILF	0.146	0.000970992
GNAI2	GNAI2B	0.148	0.000804095
NFATC4	NFAT3	0.107	0.015628085
NKD2		0.021	0.643634963
PIAS2	PIASX	0.195	9.29406E-06
PKD1		-0.150	0.000667404
RRM2	RR2	0.032	0.464811403
FBXW4	FBW4	-0.403	2.55E-21
KREMEN1	KREMEN	-0.044	0.324792246
MITF	BHLHE32	0.055	0.21802229
MAPK9	JNK2	-0.099	0.025252545
PTEN	MMAC1	0.046	0.296894074
ATP6AP2	ATP6IP2	0.145	0.001054537
RNF138	NARF	0.175	6.79605E-05
CPZ		0.210	1.65738E-06
CHD8	HELSNF1	0.036	0.415176334
CYP2D6	CYP2DL1	-0.085	0.056127411
MDC1	KIAA0170	0.051	0.2518636
PPM1A	PPPM1A	0.022	0.612798865
NR4A2	NOT	-0.200	5.4265E-06
OFD1	CXorf5	-0.290	2.34E-11
LDB1	CLIM2	-0.225	0.000000292
RNF220	C1orf164	-0.079	0.074361184
NPHP4	KIAA0673	-0.119	0.007052621
PRDM15	C21orf83	-0.074	0.096259452
PFDN5	MM1	-0.068	0.126125639
BRD7	BP75	-0.038	0.391149987
IFT20		0.055	0.215164808
MIF	GLIF	0.154	0.000474148
MAPK14	CSBP	0.091	0.039251135
TMEM170B		-0.102	0.021210051
NCSTN	KIAA0253	-0.053	0.234631649
NKD1	NKD	-0.060	0.173331363
NFATC1	NFAT2	-0.059	0.181987581
NOTUM	OK/SW-CL.30	-0.054	0.227512045
PYGO1		0.119	0.007037746

PYGO2	PP7910	-0.043	0.330656998
SFRP4	FRPHE	0.335	7.71E-15
SIAH2		0.131	0.003131363
SMURF1	KIAA1625	0.009	0.83621235
SMURF2		0.232	0.000000118
SIX2		0.032	0.469161407
SIX3		0.010	0.81647242
RUNX3	AML2	-0.008	0.864404402
SOST	UNQ2976/PRO7455/PRO7476	0.198	6.29918E-06
SOX10		-0.092	0.037269479
SOX4		0.119	0.007193713
PIP5K1B	STM7	-0.297	7.81E-12
OTUD5		-0.172	9.25307E-05
PLA2G2A	PLA2B	-0.049	0.269095754
PROP1		-0.020	0.645311885
SFRP1	FRP	-0.021	0.629702033
SFRP5	FRP1B	-0.078	0.079839572
SKI		-0.028	0.52305774
MKKS	BBS6	0.024	0.587386527
PPP2R3A	PPP2R3	0.156	0.000391785
REST	NRSF	0.040	0.371044643
SNX3		0.143	0.001244752
SMAP	C11orf58	NA	NA
MESD	KIAA0081	-0.005	0.915086163
NKX2-5	CSX	0.040	0.364213602
NPHP3	KIAA2000	-0.181	3.82423E-05
RHOV	ARHV	0.007	0.871339025
TMEM237	ALS2CR4	0.150	0.000668676
PTPN23	KIAA1471	-0.061	0.16696542
SPIN1	OCR	0.141	0.001380171
SCYL2	CVAK104	0.230	0.000000153
TMEM131L	KIAA0922	0.202	4.14602E-06
SRC	SRC1	-0.034	0.446969041
RNF31	ZIBRA	-0.002	0.9678221
SHISA3		0.008	0.863698512
RNF213	ALO17	0.055	0.21807191
SCD5	ACOD4	0.036	0.418877595
UBXN7	KIAA0794	0.161	0.000266463
TCIM	C8orf4	-0.156	0.000414196
OTULIN	FAM105B	0.201	4.78498E-06
JUP	CTNNG	-0.118	0.007405578
UBQLN1	DA41	0.146	0.000941389
SHARPIN	SIPL1	-0.087	0.050896628
SOX2		-0.007	0.872509947
SOX9		-0.033	0.450849332
SOX13		-0.345	1.1E-15
STRA6	PP14296	0.192	0.000012657
RNF43		0.074	0.095421816
STK4	KRS2	0.022	0.615850506
TREM2		0.064	0.149970088
TLE6		-0.109	0.013357682

PKD2	TRPP2	0.115	0.009130608
PLCG2		-0.052	0.24493712
PTPN11	PTP2C	0.170	0.000118121
ZBED3		-0.138	0.001782152
ZBTB12	C6orf46	0.007	0.871692919
ZBTB14	ZFP161	-0.045	0.306513722
SMARCA5	SNF2H	0.052	0.241008718
SOSTDC1	USAG1	-0.326	4.29E-14
SETDB1	ESET	-0.087	0.049274206
SOX7		-0.159	0.000299818
ATP6V0C	ATP6C	-0.082	0.064243666
SDC1	SDC	-0.020	0.649251283
USP9X	DFFRX	0.069	0.119738865
MESP1	BHLHC5	-0.089	0.044372546
PARP1	ADPRT	0.181	4.13028E-05
USP47		-0.174	7.96026E-05
RYK	JTK5A	0.172	9.67018E-05
SDHAF2	C11orf79	0.046	0.296050765
SOX17		-0.089	0.045280836
SNAI1	SNAH	0.332	1.35E-14
SEMA5A	SEMAF	0.035	0.423873687
STK11	LKB1	0.032	0.465688235
NOG		0.009	0.834441326
PPP1CA	PPP1A	0.041	0.361364775
PORCN	MG61	0.091	0.039407836
WWTR1	TAZ	0.181	4.09348E-05
DAB2	DOC2	0.097	0.029272145
DLX5		0.257	3.69E-09
FGF2	FGFB	-0.060	0.17485437
PRKN	PARK2	-0.122	0.00564173
TGFB1	TGFB	0.124	0.004995974
ROR1	NTRKR1	0.133	0.002547834
WLS	C1orf139	0.030	0.503480462
WNK2	KIAA1760	0.059	0.182648791
KRTAP10-8	KAP10.8	-0.024	0.594387186
PLPP3	LPP3	-0.057	0.196103799
TLE2		-0.134	0.002419139
PTPRU	FMI	-0.297	7.77E-12
TBX18		0.090	0.041824368
NXN	NRX	0.161	0.000255132
SNAI2	SLUG	0.335	8.17E-15
STRN		0.175	6.86535E-05
RPS27A	UBA80	0.077	0.082188291
VANGL1	STB2	0.191	0.000013374
SARDH	DMGDHL1	0.026	0.557019055
RBPJ	IGKJRB	0.119	0.007192781
STK3	KRS1	0.071	0.10823499
VCP		0.222	0.000000418
PPP2CA		0.148	0.000807226
TXNDC11	EFP1	-0.233	9.62E-08
TFCP2L1	CRTR1	-0.235	8.01E-08

TSKU	E2IG4	0.157	0.000360611
XIAP	API3	-0.138	0.001801143
USP34	KIAA0570	-0.115	0.009233941
POU5F1		-0.288	3.39E-11
TGFB1I1	ARA55	0.205	2.97879E-06
VGLL4	KIAA0121	0.157	0.000382463
PTPRO	GLEPP1	0.147	0.000856461
YAP1	YAP65	-0.076	0.085372568
USP8	KIAA0055	-0.124	0.005183237
SULF1	KIAA1077	0.519	1.91E-36
TCF7L1	TCF3	-0.230	0.000000148
PIN1		-0.025	0.575814489
USP10	KIAA0190	0.141	0.001429593
VPS29	DC15	0.145	0.001039036
MON2	KIAA1040	-0.154	0.000490452
ZNRF3	KIAA1133	-0.098	0.026374103
VSX2	CHX10	0.024	0.588650852
TBL1XR1	IRA1	0.242	2.98E-08
TAX1BP3	TIP1	-0.075	0.091412451
WDR26	CDW2	-0.127	0.004163319
SLC30A9	C4orf1	0.051	0.246036516
VAX2		0.016	0.71742072
TBL1X	TBL1	-0.020	0.644608616
TCF7L2	TCF4	-0.219	0.000000583
UBE3A	E6AP	-0.002	0.971744544
TLE3	KIAA1547	-0.012	0.787409163
USP7	HAUSP	-0.021	0.638292245
VPS35	MEM3	0.104	0.018656898
VPS26A	VPS26	0.175	6.97828E-05
TNFAIP3	OTUD7C	0.107	0.015452147
UBE2B	RAD6B	-0.002	0.970556686
WNK1	HSN2	0.170	0.000110546
WWOX	FOR	-0.064	0.150270343
UBC		0.063	0.157792556
NFATC2	NFAT1	0.076	0.087181276
UBAC2	PHGDHL1	0.097	0.027992813
SULF2	KIAA1247	0.316	2.83E-13
TLE5	AES	-0.157	0.000379897
NLE1	HUSSY-07	0.244	2.26E-08
PCDH11Y	PCDH11	-0.054	0.221789733
RECK	ST15	0.232	0.000000112
UBB		-0.139	0.001591687
PSEN1	AD3	0.038	0.390927466
TERT	EST2	0.125	0.004565257
WBP2		-0.227	0.000000221
ZEB2	KIAA0569	0.110	0.013134208
TNN	TNW	0.070	0.113878111
ZNF703	ZEPPO1	0.020	0.656304127
ZCRB1		0.062	0.16046942
TMEM64		-0.017	0.699795493
TLE1		0.075	0.089615318

WDR61		-0.102	0.021610116
TCF7	TCF1	-0.110	0.012848506
WDFY3	KIAA0993	-0.134	0.002445788
ZRANB1	TRABID	-0.085	0.055529231
TFE3	BHLHE33	-0.004	0.926894813
TLE4	GRG4	-0.154	0.00050005
UBR5	EDD	0.115	0.009571083
TRPM4	LTRPC4	-0.237	5.95E-08
VANGL2	KIAA1215	-0.108	0.015108491
TLR2	TIL4	-0.061	0.165983689
TMEM88	TMEM88A	-0.276	2.34E-10
TSC2	TSC4	-0.207	2.2957E-06
BCL7B		0.060	0.177483195
CCNYL1		0.089	0.04374901
KREMEN2	KRM2	0.151	0.000625149
LIMD1		-0.281	1.04E-10
PPM1N		0.100	0.023751868
RARG	NR1B3	-0.058	0.191178226
RACK1	GNB2L1	-0.022	0.624867534
BIRC8	ILP2	0.007	0.871097025
AFM	ALB2	-0.029	0.506403367
CELSR1	CDHF9	-0.169	0.000125577
CELSR2	CDHF10	-0.107	0.015546884
ANKS1B		-0.031	0.491247444
NPPA	ANP	-0.200	5.43301E-06
CDC42		0.046	0.304852217
CCNE1	CCNE	0.304	2.11E-12
CD2BP2	KIAA1178	-0.016	0.726100883
DLG1		0.002	0.971130294
APPL2	DIP13B	0.018	0.691216135
ASPM	MCPH5	0.288	3.35E-11
ARRB2	ARB2	-0.071	0.108744152
BMP2	BMP2A	-0.174	8.01915E-05
AMER3	FAM123C	-0.020	0.647196079
AMER2	FAM123A	-0.007	0.876531238
AMFR	RNF45	0.113	0.010952135
CCNDBP1	DIP1	-0.246	1.85E-08
AP2B1	ADTB2	0.135	0.002224149
FOXO3	FKHRL1	-0.104	0.018408517
IGFBP1	IBP1	0.046	0.300976161
IGFBP2	BP2	-0.041	0.349826696
IGFBP4	IBP4	0.039	0.377639472
IGFBP6	IBP6	0.117	0.008025302
IFT80	KIAA1374	-0.170	0.000113241
EIF4G3		0.008	0.861655215
LAMP2		0.210	1.66704E-06
HBP1		-0.016	0.718625431
HNRNPUL1	E1BAP5	0.045	0.309893547
KRTAP17-1	KAP17.1	-0.005	0.906568669
LMBR1L	KIAA1174	-0.116	0.008998513
MYC	BHLHE39	0.288	3.6E-11

NAGA		0.132	0.002885279
MYOC	GLC1A	-0.201	4.69612E-06
DAAM1	KIAA0666	0.073	0.0985174
ITCH		0.234	8.98E-08
CSNK1G2	CK1G2	0.108	0.014454758
NUCKS1	NUCKS	0.029	0.519921884
MED12	ARC240	-0.144	0.001094272
NRARP		0.089	0.0434807
VEPH1	KIAA1692	-0.249	1.15E-08
PRKCA	PKCA	0.042	0.344389149
NDP	EVR2	0.057	0.199770341
NDRG2	KIAA1248	-0.209	1.85815E-06
SLC9A3R1	NHERF	-0.036	0.417098316
GRK5	GPRK5	-0.094	0.033417442
HECW1	KIAA0322	0.275	2.82E-10
HNF1B	TCF2	-0.236	7.03E-08
MBD2		0.081	0.067027389
LYPD6	UNQ3023/PRO9821	-0.027	0.535576866
MTA1		0.004	0.934100842
FLNA	FLN	0.218	0.000000698
BTRC	BTRCP	-0.097	0.029221654
HIPK2		-0.012	0.78113415
MAD2L2	MAD2B	0.234	9.54E-08
DDX3X	DBX	-0.026	0.555747796
FOXP3	IPEX	0.164	0.000193088
LZTS2	KIAA1813	-0.089	0.044124527
MED13L	KIAA1025	-0.086	0.052673668
LSR	ILDR3	-0.082	0.064339874
CSNK2A1	CK2A1	0.129	0.003450447
CUL3	KIAA0617	-0.020	0.650745099
DKK4		-0.036	0.410809235
APPL1	APPL	0.115	0.009395818
LATS1	WARTS	0.053	0.234355158
CDC73	C1orf28	0.141	0.001437007
DYRK1B	MIRK	-0.202	4.21803E-06
DOP1B	C21orf5	-0.074	0.09341511
CLTB		-0.131	0.002931242
COL1A1		0.454	2.4E-27
BARX1		-0.006	0.896991751
BICC1		0.242	3.09E-08
PPP2R1A		-0.068	0.122842122
AMOTL2	KIAA0989	0.014	0.749337174
APP	A4	0.100	0.024238545
AXIN1	AXIN	0.020	0.651831127
CDH2	CDHN	0.304	2.47E-12
ARHGEF19		-0.151	0.000607737
CELSR3	CDHF11	0.147	0.000899074
CCNY	C10orf9	0.191	1.47491E-05
CD24	CD24A	-0.007	0.877672845
COL1A2		0.448	1.72E-26
CTNNBIP1	ICAT	-0.216	0.000000847

CMAHP	CMAH	-0.169	0.00012951
CLTC	CLH17	0.194	1.04009E-05
CLTA		0.309	1E-12
EBF2	COE2	0.059	0.184384954
DVL3	KIAA0208	0.143	0.001157289
GSN		-0.142	0.001277502
GLI1	GLI	-0.124	0.00504254
GLI3		0.215	0.000000931
GLIS2	NKL	0.168	0.000136765
GID8	C20orf11	0.019	0.667551691
BAMBI	NMA	0.012	0.780265905
ABL1	ABL	0.006	0.893132195
PRKAA1	AMPK1	-0.109	0.01363993
PRKAA2	AMPK	0.050	0.260028454
AMER1	FAM123B	0.105	0.017804403
AFDN	AF6	-0.044	0.319136644
BCL9		0.032	0.475342589
DKK3	REIC	0.140	0.001557767
DKK2	UNQ682/PRO1316	-0.021	0.640568139
FOLR1	FOLR	-0.335	7.56E-15
MLLT3	AF9	-0.004	0.933964722
ADGRA2	GPR124	0.178	5.39114E-05
CTR9	KIAA0155	-0.050	0.264178854
CTNND2	NPRAP	-0.084	0.057709535
CTHRC1	UNQ762/PRO1550	0.555	1.71E-42
DKK1	UNQ492/PRO1008	-0.051	0.251472779
DKKL1	SGY1	-0.028	0.531850155
DVL2		-0.011	0.797679756
DRAXIN	C1orf187	0.195	8.95818E-06
EMD	EDMD	-0.003	0.940751989
EDNRA	ETA	0.270	5.81E-10
AP2S1	AP17	0.175	0.000067964
EEF1D	EF1D	-0.037	0.402088733
EDARADD		0.179	0.00004954
EDAR	DL	-0.198	6.76941E-06
EDA	ED1	-0.106	0.01698737
EEF1B2	EEF1B	0.100	0.023687113
EDN1		0.013	0.771294634
DRD2		0.035	0.425476689
DVL1P1	DVL	NA	NA
ALPK2	HAK	0.422	1.85E-23
AIDA	C1orf80	0.029	0.507430764
CAV1	CAV	-0.094	0.033016052
AHI1		-0.217	0.000000754
CEMIP	KIAA1199	0.195	9.64484E-06
CFAP298	C21orf48	-0.177	5.60348E-05
CCND1	BCL1	0.055	0.217694316
CYSRT1	C9orf169	-0.159	0.000314908

Table S8: Correlation of mRNA levels between ALDH1B1 and TGF-β pathway-related molecules

Gene name	Secondary gene name	Rho	P-value
TGFBR1	ALK5	0.172	9.08067E-05
TGFBR2		-0.111	0.011957338
TGFA		0.150	0.000692166
TGFBR3		-0.262	2.01E-09
TAB1	MAP3K7IP1	-0.175	0.000069863
TAB2	KIAA0733	0.102	0.021210973
TAB3	MAP3K7IP3	-0.015	0.736380653
TGFB1	TGFB	0.124	0.004995974
TGFB3		0.323	7.42E-14
TGFB2		0.046	0.294984993
LTBP1		0.153	0.000506516
GDF15	MIC1	-0.149	0.000735561
MAP3K7	TAK1	0.220	0.000000524
LEFTY2	EBAF	-0.231	0.000000133
TGFBRAP1		0.122	0.005969984
TSPYL2	CDA1	-0.256	4.32E-09
ACVRL1	ACVRLK1	0.024	0.583097412
EIF3I	EIF3S2	0.108	0.0144361
PPP1R16B	ANKRD4	-0.046	0.295880983
STK16	MPSK1	-0.149	0.00071517
SDCBP	MDA9	0.063	0.155466864
CD109	CPAMD7	0.263	1.59E-09
TGIF2		0.125	0.004824245
ACVR1	ACVRLK2	0.182	3.52251E-05
CSRNP1	AXUD1	-0.173	8.92773E-05
CSRNP2	C12orf22	0.102	0.021155715
PDPK1	PDK1	-0.188	1.96494E-05
PEG10	EDR	0.147	0.00088895
MTMR4	KIAA0647	0.006	0.892117057
PALS1	MPP5	-0.032	0.464445795
MUC12	MUC11	-0.084	0.057942383
RHOA	ARH12	0.125	0.004731571
SCUBE3	CEGF3	0.128	0.003660855
SMAD3	MADH3	-0.103	0.020160058
TIAF1		-0.190	1.54666E-05
ZFP36L1	BERG36	0.087	0.048692316
ZFP36L2	ERF2	-0.077	0.081661714
RSPO1		-0.100	0.024633076
RSPO3	PWTSR	0.225	0.000000267
PMEPA1	STAG1	0.167	0.000147754
SNW1	SKIIP	0.186	0.000023246
SINHCAF	C12orf14	0.239	0.000000045

UBA52	UBCEP2	0.093	0.035953009
SMAD1	BSP1	0.206	2.64585E-06
SMAD5	MADH5	0.038	0.387509444
SMAD9	MADH6	-0.111	0.012195836
RNF111		-0.061	0.171945252
CITED1	MSG1	-0.042	0.346758668
HIC1	ZBTB29	0.171	0.000100236
LEFTY1	LEFTB	-0.113	0.010605436
FAM89B	Lrap25	0.116	0.009022056
LRRC32	D11S833E	0.120	0.00689233
NRROS	LRRC33	0.037	0.40828511
MAP3K7CL	C21orf7	0.056	0.207416961
DACH1	DACH	-0.179	4.80214E-05
GPER1	CEPR	-0.058	0.193769823
HSPA1A	HSP72	0.032	0.469113559
LTBP2	C14orf141	-0.004	0.926291678
BMP3	BMP3A	-0.120	0.006877357
LDLRAD4	C18orf1	-0.025	0.57336446
HSP90AA1	HSP90A	0.174	7.78338E-05
FOS	G0S7	-0.284	6.85E-11
FOXC1	FKHL7	0.133	0.00254588
DACT2	C6orf116	-0.223	0.000000344
FLOT1		-0.069	0.121535738
HTRA3	PRSP	0.438	2.78E-25
DAXX	BING2	-0.011	0.811640938
MAPK11	PRKM11	-0.042	0.345968995
GDNF		0.271	4.64E-10
HSPA1B	HSP72	0.075	0.088881357
HDAC1	RPD3L1	-0.091	0.041011538
ILK	ILK1	0.076	0.087637193
KMT5A	PRSET7	0.033	0.463688377
HDAC6	KIAA0901	-0.253	6.94E-09
FERMT1	C20orf42	0.109	0.013356913
INHBA		0.434	7.55E-25
FSTL3	FLRG	0.027	0.550414177
NSA2	TINP1	0.029	0.511660902
OGN	OIF	-0.032	0.477476781
AMH	MIF	-0.006	0.90079347
CSRNP3	FAM130A2	0.075	0.091479103
CITED2	MRG1	-0.219	0.000000601
CNIH1	CNIH	0.217	0.000000767
DACT1	DPR1	0.336	6.5E-15
ENG	END	-0.056	0.208596283
MED15	ARC105	-0.109	0.013805594
KLF10	TIEG	0.176	6.20675E-05
KLF11	FKLF	0.144	0.001131916
ARHGEF18	KIAA0521	-0.110	0.013321816
CBL	CBL2	0.132	0.002815599
DIP2A	C21orf106	-0.208	2.23695E-06
FBN1	FBN	0.344	1.26E-15
HSP90AB1	HSP90B	0.155	0.000450954

FNDC1	FNDC2	0.392	3.42E-20
ERBB2	HER2	-0.158	0.000337843
COPS5	CSN5	0.069	0.119206653
F9		0.028	0.521275314
ESR1	ESR	0.152	0.000570639
CHRD	UNQ217/PRO243	-0.040	0.373236422
FBXO11	FBX11	0.015	0.727489388
FURIN	FUR	-0.155	0.000455876
FOXH1	FAST1	-0.045	0.305765954
FOXE1	FKHL15	0.094	0.034517685
EYA2	EAB1	0.110	0.013015143
FBN2		0.224	0.000000325
KRT17		-0.019	0.671621896
JUN		-0.088	0.047843549
ELF3	ERT	-0.142	0.001338264
INHA		-0.183	3.28539E-05
INHBE		0.290	2.63E-11
CREB3L1	OASIS	-0.186	2.32189E-05
EGFR	ERBB	0.033	0.455313652
ADAMTSL2	KIAA0605	-0.075	0.089245512
MGAT5	GGNT5	0.120	0.006731925
PPP1CC		0.321	1.04E-13
ACVR2B		0.019	0.660602582
PML	MYL	0.087	0.050258791
EID2	CRI2	-0.081	0.067834907
PALLD	KIAA0992	0.400	5.12E-21
ITGB8		0.131	0.003000465
ITGB6		-0.050	0.261307483
DCN	SLRR1B	0.202	4.1482E-06
HPGD	PGDH1	-0.171	0.000103574
HTRA1	HTRA	0.306	1.59E-12
EP300	P300	-0.039	0.383230831
AXIN2		-0.174	7.64692E-05
NLK	LAK1	0.048	0.277323789
OLFM2	NOE2	0.115	0.009347389
PARD6A	PAR6A	-0.082	0.062877754
NKD2		0.021	0.643634963
PPP5C	PPP5	-0.097	0.028114165
PSG9	PSG11	0.133	0.002543379
KDM8	JMJD5	-0.173	8.93804E-05
PRKCZ	PKC2	-0.329	2.29E-14
PRDM16	KIAA1675	-0.236	6.73E-08
PSPN		-0.063	0.158551359
PPM1A	PPPM1A	0.022	0.612798865
NRTN		0.117	0.008232611
LEMD3	MAN1	-0.004	0.922772451
LTBP3		-0.136	0.002115727
PPP1R15A	GADD34	-0.083	0.060999647
MAPK14	CSBP	0.091	0.039251135
SGK1	SGK	-0.117	0.008238679
SMURF1	KIAA1625	0.009	0.83621235

SMURF2		0.232	0.000000118
SKIL	SNO	0.161	0.000267121
RUNX3	AML2	-0.008	0.864404402
SAP30L	NS4ATP2	-0.156	0.000413251
SKI		-0.028	0.52305774
SKOR2	CORL2	NA	NA
SEPTIN4	C17orf47	-0.032	0.465467609
INPP5D	SHIP	-0.020	0.644292032
SEPTIN9	KIAA0991	0.014	0.744632402
SMAD4	DPC4	0.007	0.870955466
TM4SF20	UNQ518/PRO994	-0.072	0.102806335
SLC39A5	ZIP5	-0.117	0.008057106
TSC22D1	KIAA1994	-0.103	0.020395618
RORC	NR1F3	-0.207	2.32865E-06
SMAD6	MADH6	-0.357	8.4E-17
SNIP1		0.078	0.078728413
STRAP	MAWD	0.247	0.000000016
SNTA1	SNT1	0.151	0.000649745
TGFBR3L		NA	NA
NODAL		-0.022	0.616035469
VAV3		0.038	0.386503915
SOSTDC1	USAG1	-0.326	4.29E-14
SMAD2	MADH2	0.030	0.50360339
VASN	SLITL2	0.069	0.118762051
RHBDF1	C16orf8	-0.217	0.000000715
USP9X	DFFRX	0.069	0.119738865
TXNIP	VDUP1	-0.142	0.001292201
UCHL5	UCH37	0.222	0.000000405
ZBED2		0.082	0.063781961
SNAI1	SNAH	0.332	1.35E-14
SMAD7	MADH7	0.097	0.028898591
IFIT5	ISG58	0.076	0.08814805
MXRA5		0.377	1.03E-18
PPP1CA	PPP1A	0.041	0.361364775
WWTR1	TAZ	0.181	4.09348E-05
DAB2	DOC2	0.097	0.029272145
LTBP4		-0.192	1.33158E-05
NEDD4L	KIAA0439	-0.295	1.12E-11
XPO1	CRM1	0.155	0.000440847
TDP2	EAP2	-0.038	0.39439766
SNAI2	SLUG	0.335	8.17E-15
RPS27A	UBA80	0.077	0.082188291
WWP1		-0.090	0.041756045
STK3	KRS1	0.071	0.10823499
ZNF8		0.092	0.036854901
TGIF2LY	TGIFLY	0.165	0.000176434
PTPN14	PEZ	0.032	0.465420167
YAP1	YAP65	-0.076	0.085372568
ZNF580		-0.185	2.61675E-05
TGIF2LX	TGIFLX	0.231	0.000000125
TTC3	DCRR1	-0.072	0.102453433

TRIM33	KIAA1113	-0.043	0.332673216
TRAF6	RNF85	-0.031	0.486656871
USP9Y	DFFRY	-0.066	0.13730207
UBC		0.063	0.157792556
ZMIZ1	KIAA1224	-0.120	0.006548434
LAPTM4B	PSEC0001	0.152	0.000552695
TOE1		-0.149	0.000707302
NEDD8		0.150	0.000679289
PARD3	PAR3	0.134	0.002496702
UBB		-0.139	0.001591687
USP15	KIAA0529	0.104	0.018591653
ZBTB7A	FBI1	-0.146	0.000915514
ZNF451	COASTER	-0.013	0.771257219
VPS39	KIAA0770	-0.376	1.42E-18
WDR7	KIAA0541	NA	NA
ZFHX3	ATBF1	-0.018	0.685894004
ZFYVE9	MADHIP	-0.185	2.74499E-05
UBE2M	UBC12	0.099	0.025007891
PPP1CB		0.172	9.32753E-05
RCCD1		-0.064	0.148830583
RBBP9	BOG	-0.044	0.321373399
RB1CC1	KIAA0203	0.052	0.236991126
RANBP3		-0.122	0.005673276
BMP6	VGR	-0.119	0.007217264
BMP7	OP1	-0.077	0.081236383
ASPN	PLAP1	0.342	1.9E-15
ARRB2	ARB2	-0.071	0.108744152
BMP2	BMP2A	-0.174	8.01915E-05
BMP5		-0.039	0.385103182
BMP8B	BMP8	0.232	0.000000114
BMPR1A	ACVRLK3	0.055	0.216059702
FSTL1	FRP	0.296	9.77E-12
IL31RA	CRL3	0.118	0.007760847
CHUK	IKKA	0.154	0.000490011
IKBKB	IKKB	-0.128	0.003769382
CSNK1G2	CK1G2	0.108	0.014454758
INHBB		0.030	0.502485836
VEPH1	KIAA1692	-0.249	1.15E-08
JTB	HSPC222	0.020	0.657404956
IKBKG	FIP3	-0.053	0.229618707
CDKN2B	MTS2	0.051	0.247604185
INHBC		0.053	0.22893324
GSDMA	GSDM	0.071	0.109082584
FOXP3	IPEX	0.164	0.000193088
MECOM	EVI1	-0.294	1.27E-11
APPL1	APPL	0.115	0.009395818
F11R	JAM1	-0.234	8.92E-08
ITGAV	MSK8	0.358	7.63E-17
ACVR1B	ACVRLK4	-0.129	0.003532639
DTL	CDT2	0.315	3.51E-13
BMP15	GDF9B	-0.030	0.500869435

BMP1	PCOLC	0.181	3.99097E-05
BMPR2	PPH1	0.035	0.428580679
BMPR1B		0.053	0.232447718
AIMP1	EMAP2	0.074	0.093376549
AXIN1	AXIN	0.020	0.651831127
ACVR1C	ALK7	0.113	0.010587522
CD44	LHR	0.071	0.109884055
CMKLR1	CHEMR23	0.084	0.057164664
STUB1	CHIP	-0.094	0.033962286
CGN	KIAA1319	-0.228	0.000000186
CILP	UNQ602/PRO1188	0.426	7.38E-24
CLEC6A	CLECSF10	0.225	0.000000273
GDF10	BMP3B	-0.274	3.03E-10
GDF11	BMP11	0.260	2.67E-09
GDF1		0.068	0.125159541
GDF2	BMP9	-0.144	0.00108504
GDF3	UNQ222/PRO248	0.023	0.604206704
GDF5	BMP14	0.100	0.023379147
GDF6	BMP13	0.217	0.000000723
GDF7		-0.160	0.000285125
MSTN	GDF8	-0.145	0.0010501
GFRA1	GDNFRA	-0.220	0.000000519
GFRA2	GDNFRB	-0.086	0.050956584
GDF9		-0.037	0.401034168
BAMBI	NMA	0.012	0.780265905
BMP10		0.006	0.889152529
BMP4	BMP2B	0.022	0.621287438
BMP8A		0.349	4.61E-16
AGRN	AGRIN	-0.124	0.004969939
TGFBI	BIGH3	0.342	1.93E-15
FKBP1A	FKBP1	0.145	0.000998538
CREBBP	CBP	-0.084	0.057155558
ARTN	EVN	0.061	0.171651293
CX3CR1	CMKBRL1	-0.106	0.016766426
CAV1	CAV	-0.094	0.033016052
CERS1	LAG1	0.032	0.475790752
EWSR1	EWS	-0.167	0.000158376

Table S9: Correlation of mRNA levels between ALDH1B1 and Notch pathway-related molecules

Gene name	Secondary gene name	Rho	P-value
NOTCH1	TAN1	-0.063	0.157203737
NOTCH2		0.139	0.001702075
NOTCH3		0.134	0.002454911
NOTCH4	INT3	-0.071	0.11051667
NOTCH2NLA	N2N	0.004	0.934807409
NOTCH2NLC		NA	NA
MINAR1	KIAA1024	0.194	9.82926E-06

DNER	BET	-0.046	0.296515097
CHAC1	BOTCH	0.046	0.303090294
EGFL7	MEGF7	-0.197	7.62056E-06
SBNO2	KIAA0963	0.017	0.700367819
NRARP		0.089	0.0434807
SBNO1	MOP3	0.217	0.000000717
PSEN1	AD3	0.038	0.390927466
DLL1	UNQ146/PRO172	0.022	0.625434876
JAG1	JAGL1	0.047	0.292598938
MAML1	KIAA0200	-0.115	0.009058811
RBPJ	IGKJRB	0.119	0.007192781
MAML2	KIAA1819	-0.016	0.715869323
DLL4	UNQ1895/PRO4341	0.028	0.532733294
FBXW7	FBW7	-0.024	0.590660738
DTX1		-0.025	0.576982853
SNW1	SKIIP	0.186	0.000023246
JAG2		0.102	0.021367978
APH1A	PSF	0.093	0.035856611
NCSTN	KIAA0253	-0.053	0.234631649
KDM1A	AOF2	0.109	0.013687779
LCE5A	LEP18	-0.034	0.448623349
KRT83	KRTHB3	-0.054	0.223454428
MFSD13A	C10orf77	-0.147	0.000839187
POGLUT1	C3orf9	0.160	0.000273863
PDPK1	PDK1	-0.188	1.96494E-05
PTGER3		0.158	0.000355316
PGAM2	PGAMM	-0.043	0.334250159
PSENEN	PEN2	-0.022	0.614821087
PGAP6	TMEM6	-0.070	0.114733403
PCED1A	C20orf81	-0.247	1.53E-08
PCED1B	FAM113B	0.128	0.003791082
PID1	NYGGF4	-0.111	0.01189219
PDCD10	CCM3	0.216	0.000000881
PCSK5	PC5	0.014	0.753063608
PDE9A		-0.124	0.005162514
PLXND1	KIAA0620	-0.027	0.546620089
PTK7	CCK4	0.115	0.009493344
PSMD11		0.271	4.68E-10
PSMB9	LMP2	0.157	0.000370623
SMARCE1	BAF57	0.121	0.006143273
TSPAN5	TM4SF9	0.200	5.20934E-06
UXT	HSPC024	0.043	0.337739734
POFUT1	FUT12	0.272	4.22E-10
SPEN	KIAA0929	-0.129	0.003530653
PBX1	PRL	-0.161	0.000273352
RASD1	AGS1	-0.111	0.012507945
RAB6A	RAB6	0.187	2.22136E-05
LMO2	RBTN2	-0.028	0.524684422
RBX1	RNF75	0.036	0.417621393
PSMC4	MIP224	0.115	0.009467971
RBPJL	RBPL	0.007	0.86961776

PERP	KCP1	0.047	0.286170929
SMARCA4	BAF190A	0.102	0.021349543
SMAD3	MADH3	-0.103	0.020160058
SEM1	C7orf76	0.149	0.000718446
TRB		NA	NA
SEMA4C	KIAA1739	0.073	0.101386533
SKP2	FBXL1	0.267	8.81E-10
TRAF7	RFWD1	0.027	0.548621518
TINAGL1	GIS5	-0.110	0.013267909
TMED2	RNP24	0.232	0.00000012
ZFP36L1	BERG36	0.087	0.048692316
ZFP36L2	ERF2	-0.077	0.081661714
PSMA7	HSPC	0.187	2.16913E-05
RITA1	C12orf52	0.207	2.36398E-06
RUNX1	AML1	0.144	0.001123565
PSMA6	PROS27	0.280	1.26E-10
PSMC5	SUG1	0.007	0.880719704
PRPF31	PRP31	-0.023	0.600090846
ST3GAL4	CGS23	0.074	0.095616321
SNED1		-0.180	4.44906E-05
SMOC1		-0.070	0.116661168
SKP1	EMC19	-0.006	0.899141967
TNS2	KIAA1075	-0.362	2.78E-17
SPPL2C	IMP5	0.022	0.615982331
AGXT	AGT1	-0.016	0.713569841
TSPAN15	NET7	-0.010	0.82366044
UBA52	UBCEP2	0.093	0.035953009
ROBO2	KIAA1568	-0.255	5.16E-09
SELENOM	SELM	0.157	0.000369108
TNIP3	ABIN3	0.066	0.133713285
PTP4A3	PRL3	0.143	0.001162285
TMEM41A	UNQ168/PRO194	0.154	0.000499849
SHFL	C19orf66	-0.099	0.024757656
SLC22A5	OCTN2	-0.341	2.23E-15
TRIM27	RFP	-0.079	0.073994858
SORBS2	ARGBP2	-0.286	4.95E-11
PSMB6	LMPY	0.012	0.794233119
PSMB4	PROS26	0.082	0.063307255
PSEN2	AD4	-0.135	0.002248366
STAT3	APRF	-0.103	0.019610997
CRH		-0.014	0.745303382
HIF1A	BHLHE78	0.313	4.45E-13
HDAC9	HDAC7	0.101	0.021967731
HIF1AN	FIH1	-0.119	0.007040119
GABPB1	E4TF1B	0.260	2.49E-09
GABPA	E4TF1A	NA	NA
HSPBP1	HSPBP	0.036	0.418253323
BMP2K	BIKE	0.134	0.002421061
BRME1	C19orf57	0.126	0.00423316
CRTC1	KIAA0616	-0.228	0.000000192
INSM1	IA1	0.015	0.741036772

H2BC17	H2BFH	0.104	0.019354263
KLK8	NRPN	NA	NA
H4C1	H4/A	0.056	0.208898512
KRTAP3-1	KAP3.1	0.142	0.00128557
LMNTD2	C11orf35	-0.302	3.37E-12
SMCP	MCS	0.071	0.109363236
LYVE1	CRSBP1	-0.007	0.868495954
ASCL1	ASH1	-0.026	0.562393918
ASPSCR1	ASPL	-0.140	0.001513308
DLX4	BP1	-0.151	0.000618332
ELANE	ELA2	-0.124	0.005063414
GNPTAB	GNPTA	-0.011	0.81111621
KCTD15		0.035	0.432013886
MOV10	KIAA1631	-0.044	0.317280285
ADCK5		-0.118	0.007432854
SLC25A6	AAC3	-0.061	0.166922764
ITGB2	CD18	0.087	0.048598664
ITGB4		-0.097	0.028198215
ITGB5		0.279	1.49E-10
MMP21		-0.095	0.032762023
GXYLT2	GLT8D4	0.349	4.34E-16
H2AZ1	H2AFZ	0.292	1.83E-11
H2BC13	H2BFC	0.131	0.002985424
LONRF3	RNF127	-0.127	0.004194346
IRF6		-0.063	0.153351931
KCNA5		-0.089	0.045295882
LCE2B	LEP10	-0.042	0.342410663
CRB2		-0.118	0.00759065
FZD10		-0.010	0.821999985
FOXC1	FKHL7	0.133	0.00254588
KRTAP26-1	KAP26.1	-0.063	0.154806214
KRTAP13-2	KAP13.2	-0.061	0.165745384
LIMS2	PINCH2	-0.006	0.891318767
ADAMTSL5	THSD6	0.017	0.698406914
CDKN1B	KIP1	0.001	0.976566467
DTX2	KIAA1528	-0.005	0.911895195
ENHO	C9orf165	-0.077	0.082276385
FRS3		-0.241	3.39E-08
H2BC14	H2BFE	0.065	0.141359465
H2BC21	H2BFQ	-0.040	0.367372717
HBA1;	HBA2	NA	NA
HDAC11		-0.210	1.64625E-06
HAPLN2	BRAL1	-0.138	0.001839972
INO80B	HMGA1L4	-0.081	0.068536465
KRTAP4-11	KAP4.14	-0.010	0.822839073
KRTAP9-8	KAP9.8	0.045	0.315023965
KRTAP4-5	KAP4.5	0.062	0.159713006
LNX1	LNX	-0.177	5.88184E-05
HES1	BHLHB39	0.028	0.533963471
HES3	BHLHB43	0.036	0.423613251
HDAC2		0.265	1.22E-09

LLGL2		-0.307	1.39E-12
NPPB		0.046	0.301390603
EPHB6		-0.162	0.000239667
H2AC6	H2AFL	-0.054	0.224801535
HHEX	HEX	-0.016	0.710854792
LFNG		-0.009	0.843487984
ANGPT4	ANG3	-0.015	0.740339061
ARID3A	DRIL1	0.093	0.035436818
ITGB1BP1	ICAP1	0.276	2.24E-10
LCE1F	LEP6	-0.015	0.741422987
LCE2D	LEP12	-0.005	0.904515814
SLC43A2	LAT4	-0.140	0.001569642
KRTAP5-4	KAP5.4	-0.017	0.708624176
MACO1	TMEM57	-0.112	0.011389133
MINAR2	KIAA1024L	NA	NA
EFNA3	EFL2	0.110	0.013049226
FBXW5	FBW5	-0.186	2.27833E-05
LILRB2	ILT4	0.216	0.000000895
NECTIN3	PRR3	-0.035	0.433163405
DLK1	DLK	-0.114	0.009693855
FAM124B		0.011	0.805990334
GLP1R		-0.145	0.000997026
H2AZ2	H2AFV	0.120	0.006861221
H3C1	H3FA	0.075	0.091545976
H3C15	HIST2H3A;	NA	NA
H2BC1	HIST1H2BA	0.084	0.058361829
H2BU1	HIST3H2BB	-0.039	0.377845399
EIF3F	EIF3S5	0.003	0.946500307
KRTAP2-4	KAP2.4	-0.002	0.964836371
HES4	BHLHB42	0.091	0.039025286
KRTAP4-2	KAP4.2	-0.067	0.129994838
KRTAP4-4	KAP4.13	0.077	0.081497041
HDAC1	RPD3L1	-0.091	0.041011538
HSPA12B	C20orf60	-0.022	0.61999153
EIF4E2	EIF4EL3	0.027	0.539971189
SPINK2		0.055	0.214831437
KRT20		0.032	0.473189166
PRKCI	DXS1179E	-0.005	0.909701619
NUMBL		0.215	1.01214E-06
HDAC4	KIAA0288	-0.090	0.041820747
HDAC5	KIAA0600	-0.084	0.058420829
HDAC6	KIAA0901	-0.253	6.94E-09
HDAC7	HDAC7A	-0.183	3.12589E-05
HDAC8	HDACL1	-0.130	0.003153842
H2BC9	H2BFJ	0.118	0.007810273
NEU2		0.119	0.007086628
NCOR1	KIAA1047	-0.125	0.004700605
NTM	IGLON2	0.308	1.08E-12
NUBP2		-0.016	0.720967825
NTN4		-0.205	3.03797E-06
NMU		0.083	0.060986371

CYHR1	KIAA0496	-0.124	0.004945676
LCE1B	LEP2	-0.024	0.594638721
KRTAP10-9	KAP10.9	-0.007	0.876419857
CEBPA	CEBP	-0.225	0.000000293
DGKQ	DAGK4	-0.208	2.13125E-06
CDK3	CDKN3	-0.294	1.25E-11
CDK6	CDKN6	0.143	0.001203601
FAM221A	C7orf46	-0.147	0.000874791
BATF		0.007	0.882791067
CHDH		-0.038	0.390595204
DHRS1	SDR19C1	-0.286	5E-11
CNTN6		-0.267	9.26E-10
KCNS2	KIAA1144	-0.040	0.361929338
KRTAP9-4	KAP9.4	-0.004	0.92895822
KRTAP5-9	KAP5.9	-0.238	5.21E-08
KRTAP9-2	KAP9.2	0.016	0.713416063
LCE3D	LEP16	0.085	0.056129837
CTSZ		0.025	0.572014417
CCDC26	RAM	NA	NA
GEM	KIR	-0.059	0.186396798
LCE3E	LEP17	-0.024	0.591069204
KRTAP9-3	KAP9.3	NA	NA
KRTAP12-1	KAP12.1	0.016	0.720364593
NCOR2	CTG26	-0.002	0.972311684
CD164		-0.058	0.189144392
H2AC14	H2AFE	0.079	0.076453937
H2AC20	H2AFQ	0.128	0.00374598
H2BC5	H2BFB	0.055	0.215751261
GSAP	PION	-0.251	9.15E-09
JAK2		0.057	0.196254053
AGO1	EIF2C1	-0.036	0.412622258
CDK8		0.273	3.45E-10
CST9L	CTES7B	0.069	0.117089688
FBN1	FBN	0.344	1.26E-15
HSBP1	HSF1BP	-0.069	0.117261989
CATSPER1		0.201	4.87966E-06
FAM90A1		0.076	0.084963567
FAAH	FAAH1	-0.321	1.02E-13
DLX1		0.197	7.40464E-06
FGF10		0.027	0.5460257
CCER1	C12orf12	0.047	0.289798246
CHRD	UNQ217/PRO243	-0.040	0.373236422
E2F1	RBBP3	0.196	8.00245E-06
CNTN1		0.070	0.114532034
E2F3	KIAA0075	0.245	2.15E-08
HES7	BHLHB37	0.041	0.359422614
FURIN	FUR	-0.155	0.000455876
GIP		0.130	0.003155819
EPN2	KIAA1065	-0.076	0.087889659
H2AX	H2AFX	0.249	1.14E-08
GXYLT1	GLT8D3	-0.096	0.029747221

H2BC12	H2BFT	-0.037	0.404834044
H2AC4	H2AFM	0.057	0.200145888
H2AC7	H2AFG	0.068	0.125604536
JOSD1	JSPH1	0.197	7.41485E-06
H2AB1	H2AFB1	-0.019	0.669785176
JUN		-0.088	0.047843549
HBZ	HBZ2	-0.137	0.001872365
SLC35C1	FUCT1	-0.080	0.070126819
GATA2		-0.068	0.126657088
GATA5		-0.177	5.93017E-05
GAS2		-0.169	0.000129915
ATP5F1D	ATP5D	0.018	0.682302156
ELF3	ERT	-0.142	0.001338264
GSTP1	FAEES3	-0.191	1.38649E-05
GRIP2	KIAA1719	0.221	0.000000483
H2BC15	H2BFD	-0.017	0.699938175
DALRD3		-0.136	0.00201789
LINC00526	C18orf18	0.066	0.139382748
CSF1		0.170	0.000117419
EGFR	ERBB	0.033	0.455313652
FAM71C		-0.070	0.112542306
H2AC18	H2AFO	0.021	0.633795677
EOGT	AER61	0.054	0.219811047
KRTAP4-12	KAP4.12	0.061	0.165755608
LCE1E	LEP5	0.010	0.822737004
LCE3C	LEP15	0.052	0.241700725
LCE3A	LEP13	0.052	0.239759353
CHCHD3	MIC19	0.353	1.96E-16
MDK	MK1	0.112	0.011584701
MRGBP	C20orf20	0.213	1.28171E-06
POMGNT2	AGO61	0.067	0.129754215
PSMA5		0.126	0.00452948
LCE4A	LEP8	-0.009	0.84141534
KRTAP13-3	KAP13.3	0.009	0.846723496
NFKB1		-0.016	0.718227668
DLGAP5	DLG7	0.376	1.47E-18
HPCAL1	BDR1	-0.160	0.000286266
MMP14		0.380	6.04E-19
PSMD14	POH1	0.216	0.000000842
DLX2		0.127	0.004021183
HDAC3		0.039	0.378411565
HEY2	BHLHB32	0.129	0.003634073
CREB1		0.078	0.078665396
MOS		0.094	0.033330499
KAT2B	PCAF	-0.166	0.00016701
KRT19		-0.010	0.825949395
LRFN4	SALM3	0.229	0.000000173
PAPPA		-0.017	0.696328947
PLN	PLB	0.289	2.87E-11
KRTAP10-7	KAP10.7	-0.078	0.079416595
KRTAP12-3	KAP12.3	0.024	0.583654695

NKAPL	C6orf194	0.055	0.212649972
P2RX4		0.064	0.150221959
ATG9A	APG9L1	-0.033	0.462004869
FOXB1	FKH5	0.072	0.104959857
GIT1		0.191	1.47152E-05
MESP2	BHLHC6	0.087	0.049421405
GRN		-0.084	0.057740208
H2AJ	H2AFJ	-0.005	0.913289257
EP300	P300	-0.039	0.383230831
F10		-0.131	0.003097972
FAM74A4	FAM74A2;	-0.043	0.333001859
H2BC4	H2BFL	-0.017	0.709971301
H2BC11	H2BFR	0.101	0.022289222
GZMB	CGL1	0.220	0.0000005
LCE1D	LEP4	-0.031	0.478366919
LLGL1	DLG4	0.102	0.021263925
EGF		0.031	0.478533174
ETV2	ER71	-0.098	0.026782491
KRTAP4-7	KAP4.7	0.058	0.189904052
CUL1		0.133	0.002715852
IL10		0.130	0.003383059
MXI1	BHLHC11	0.063	0.152820016
NEURL1	NEURL	-0.051	0.254738258
NEURL1B	NEURL3	0.219	0.00000056
NLK	LAK1	0.048	0.277323789
OLFM2	NOE2	0.115	0.009347389
FOXN4		-0.114	0.010007969
GLYAT	ACGNAT	-0.039	0.383436072
GNAI2	GNAI2B	0.148	0.000804095
KRTAP10-10	KAP10.10	0.037	0.404836598
NR0B2	SHP	-0.186	2.26681E-05
P2RY6	PP2891	0.317	2.27E-13
NIBAN2	C9orf88	-0.126	0.004487722
NECTIN2	HVEB	-0.033	0.451624969
NOD2	CARD15	0.047	0.291128916
NAXD	CARKD	-0.290	2.46E-11
NPDC1		-0.180	4.31216E-05
TP53	P53	0.029	0.51362934
PRF1	PFP	0.019	0.670545864
PSMB8	LMP7	0.074	0.095308026
DAGLB		-0.084	0.059382029
KPRP	C1orf45	0.018	0.681873709
OTX1		0.039	0.380044481
PLSCR4	GIG43	-0.006	0.885446572
PSMA3	HC8	0.128	0.003732448
PSMD4	MCB1	0.027	0.536171444
PSMB1	PSC5	0.125	0.004842385
CCNC		0.115	0.009180239
PSMF1		0.033	0.461859631
MSX1	HOX7	0.132	0.002833423
NECTIN1	HVEC	0.081	0.066651822

METTL3	MTA70	-0.066	0.138358076
NOS3		0.191	1.37896E-05
KRTAP5-6	KAP5.6	0.046	0.302573538
KRTAP10-11	KAP10.11	-0.039	0.373937237
POSTN	OSF2	0.500	1.42E-33
PRR35	C16orf11	0.044	0.323872393
PSMC3	TBP1	0.104	0.01911844
MEGF10	KIAA1780	0.273	3.62E-10
TACC3	ERIC1	0.259	2.74E-09
NR1H4	BAR	-0.182	0.000037197
NPBWR2	GPR8	-0.029	0.506265502
SIRT1	SIR2L1	0.008	0.850132373
ST3GAL3	SIAT6	-0.111	0.012370823
SGK1	SGK	-0.117	0.008238679
SIRT6	SIR2L6	-0.006	0.895111417
SIX1		0.131	0.003124184
RUNX3	AML2	-0.008	0.864404402
MRPL40	NLVCF	-0.005	0.915188836
SLC5A5	NIS	-0.058	0.187464372
NEPRO	C3orf17	0.214	1.07446E-06
PKHD1	FCYT	-0.207	2.47411E-06
NKAP		0.040	0.369923951
PBX2	G17	-0.234	8.79E-08
PSMB2		0.150	0.000668692
SYCP3	SCP3	-0.064	0.15126887
PSMD8		0.101	0.022208619
SPG7	CAR	-0.258	3.24E-09
OVOL2	ZNF339	-0.250	1.09E-08
P2RX7		0.124	0.005164473
PSMD9		0.183	3.24187E-05
SCNM1		0.155	0.000436206
PSMB3		0.192	1.21706E-05
RIPPLY2	C6orf159	-0.008	0.849287877
INPP5D	SHIP	-0.020	0.644292032
TMEM231	UNQ870/PRO1886	-0.039	0.375041158
PTPN23	KIAA1471	-0.061	0.16696542
STAT1		0.186	2.47791E-05
SPRY1		-0.121	0.006348781
SRC	SRC1	-0.034	0.446969041
SPATA8		0.222	0.000000416
SLC35C2	C20orf5	-0.107	0.015684091
SLC23A1	SVCT1	-0.191	1.41197E-05
PSMB5	LMPX	0.173	8.36453E-05
POGLUT3	KDELC2	0.227	0.000000227
PSMD7	MOV34L	0.132	0.002816502
PSMB7	Z	0.112	0.011489039
PSMB10	LMP10	-0.056	0.202899721
SEL1L	TSA305	0.178	5.07852E-05
TCIM	C8orf4	-0.156	0.000414196
TAPBPL		-0.069	0.117645571
MSX2	HOX8	0.177	5.79127E-05

CBFA2T2	EHT	-0.123	0.005555449
ALPP	PLAP	-0.226	0.000000252
SUMO1	SMT3C	0.107	0.015249856
PSME1	IFI5111	0.090	0.041101063
SLC22A23	C6orf85	-0.295	1.09E-11
MELTF	MAP97	0.230	0.00000015
SOX9		-0.033	0.450849332
SREBF2	BHLHD2	-0.155	0.000435054
TCF3	BHLHB21	0.270	5.99E-10
SLC6A20	SIT1	0.021	0.635827545
RGL2	RAB2L	-0.311	6.99E-13
STK16	MPSK1	-0.149	0.00071517
MDM2		-0.163	0.000212638
PLPP2	LPP2	-0.018	0.686456849
TOX	KIAA0808	-0.146	0.000931448
PSMD1		0.159	0.000306201
ST3GAL6	SIAT10	-0.119	0.007100851
FASLG	APT1LG1	0.032	0.475827691
TSPAN33	PEN	0.087	0.050186412
VASN	SLITL2	0.069	0.118762051
PSMD10		0.176	6.42774E-05
PSME3		0.309	9.47E-13
SRCAP	KIAA0309	-0.194	9.8021E-06
GTF3C5	CDABP0017	0.008	0.859664866
UTP23	C8orf53	0.244	2.51E-08
MESP1	BHLHC5	-0.089	0.044372546
RFNG		-0.011	0.799508074
TSPEAR	C21orf29	-0.223	0.000000353
PSMC1		0.113	0.010477901
ALPI		-0.050	0.259068577
ROBO1	DUTT1	0.132	0.002851871
FARS2	FARS1	-0.018	0.680022589
S1PR3	C9orf108	0.225	0.000000298
SDCBP	MDA9	0.063	0.155466864
SNAI1	SNAH	0.332	1.35E-14
SEMA3B	SEMA5	-0.216	0.00000082
ELOC	TCEB1	0.247	1.59E-08
FABP7	BLBP	0.062	0.16431077
KRTAP12-4	KAP12.4	0.039	0.384796777
KRTAP13-4	KAP13.4	-0.030	0.496918042
LRCH4	LRN	-0.302	3.51E-12
PSMD12		0.304	2.14E-12
TBX6		-0.207	2.33864E-06
KRTAP10-8	KAP10.8	-0.024	0.594387186
PLEKHN1	CLPABP	0.009	0.835885261
TLE2		-0.134	0.002419139
PRR20D		NA	NA
STAC	STAC1	0.089	0.045732128
TSPAN4	NAG2	-0.141	0.001429726
MXD3	BHLHC13	-0.007	0.874954614
TGFB2		0.046	0.294984993

TM2D3	BLP2	-0.168	0.000137378
SNAI2	SLUG	0.335	8.17E-15
RPS28		-0.031	0.483482763
RPS27A	UBA80	0.077	0.082188291
PVR	PVS	0.179	0.000048255
SPATA24		-0.047	0.286276045
ZDHHC1	C16orf1	-0.275	2.7E-10
ZNF446	ZKSCAN20	-0.280	1.15E-10
PSMD3		0.210	1.65074E-06
PSMD2	TRAP2	0.264	1.47E-09
SUSD5	KIAA0527	0.210	1.66982E-06
UBR7	C14orf130	0.157	0.000367197
XXYLT1	C3orf21	0.297	7.52E-12
PSMA2	HC3	0.269	6.62E-10
TFCP2L1	CRTR1	-0.235	8.01E-08
TROAP		0.244	2.38E-08
XIAP	API3	-0.138	0.001801143
ZNF32	KOX30	-0.028	0.52812557
PSMC2	MSS1	0.267	9.01E-10
TBC1D10C		-0.061	0.16889891
PSMA1	HC2	0.114	0.01017893
PSMC6	SUG2	0.157	0.000363285
ZFP64	ZNF338	0.170	0.000119801
ZNF837		-0.145	0.001003462
ZNF417		-0.069	0.117518945
WDR12		0.353	2.17E-16
TFDP1	DP1	0.239	4.79E-08
ZNF581	HSPC189	0.108	0.01489737
THAP7		-0.091	0.039932971
YAP1	YAP65	-0.076	0.085372568
PSMD5	KIAA0072	0.140	0.001524555
PSME2		0.162	0.000230342
POGLUT2	EP58	0.437	3.01E-25
XCL2	SCYC2	0.031	0.485285209
PSMG2	HCCA3	0.074	0.093610344
PIN1		-0.025	0.575814489
TMEM150A	TMEM150	-0.151	0.000640511
YBX1	NSEP1	0.232	0.000000122
ZNF648		0.024	0.581133204
ZNF580		-0.185	2.61675E-05
PSMD6	KIAA0107	0.171	0.000104287
TIMP4		0.126	0.004507189
PAPPA2	PLAC3	-0.083	0.062125196
WT1-AS	WIT1	0.396	1.48E-20
TTPA	TPP1	-0.137	0.001888109
YIPF3	C6orf109	-0.112	0.011438788
THEMIS2	C1orf38	0.147	0.000847056
TMEM100		-0.182	3.57207E-05
THBS4	TSP4	0.105	0.017841739
TSPAN14	TM4SF14	-0.137	0.001867354
TBL1XR1	IRA1	0.242	2.98E-08

ZNF330	NOA36	-0.041	0.357786568
TNRC6C	KIAA1582	-0.206	2.77836E-06
TNRC6B	KIAA1093	-0.124	0.004928684
ZNF587		-0.108	0.014258292
PRPS2		0.229	0.000000163
TBL1X	TBL1	-0.020	0.644608616
TRIM42		0.048	0.283707184
TLE3	KIAA1547	-0.012	0.787409163
ZNF672		-0.136	0.002145651
ZNF440		-0.150	0.000678055
MFNG		-0.025	0.57931527
WDR25	C14orf67	-0.080	0.071609455
ZNF439		-0.029	0.515854634
ZNF786		0.032	0.469340234
WFS1		-0.197	7.50961E-06
ZNF124		0.114	0.010195902
TFDP2	DP2	0.224	0.000000303
UBC		0.063	0.157792556
WWP2		-0.321	1.08E-13
ZNF423	KIAA0760	0.074	0.096666702
ZMIZ1	KIAA1224	-0.120	0.006548434
KRTAP12-2	KAP12.2	0.071	0.109811533
RELA	NFKB3	0.161	0.000259911
TP63	KET	-0.024	0.596347415
PIGS	UNQ1873/PRO4316	0.097	0.028121145
SYNJ2BP	OMP25	-0.159	0.000314327
TBX2		-0.203	3.98038E-06
TNRC6A	CAGH26	-0.147	0.000854295
RET	CDHF12	-0.005	0.918678841
TLE5	AES	-0.157	0.000379897
NLE1	HUSSY-07	0.244	2.26E-08
PKMYT1	MYT1	0.315	3.14E-13
UBB		-0.139	0.001591687
YJEFN3	AIBP2	-0.121	0.006196246
		NA	NA
ZNF408	PFM14	-0.007	0.88191047
ZBTB7A	FBI1	-0.146	0.000915514
ZNF319	KIAA1388	-0.075	0.088820884
PSMA4	HC9	0.029	0.507181977
PSMD13		0.135	0.002251388
YTHDF2	HGRG8	-0.021	0.630950836
TMEFF2	HPP1	-0.149	0.000708565
TLE1		0.075	0.089615318
ZNF488		0.130	0.003220342
ZNF497		-0.220	0.000000555
RCHY1	ARNIP	0.001	0.981462395
TGFBR2		-0.111	0.011957338
TCAF1	FAM115A	0.055	0.218838442
TLE4	GRG4	-0.154	0.00050005
PTPMT1	MOSP	-0.004	0.92591281
TRAPPC14	C7orf43	-0.033	0.452222208

WNT1	INT1	-0.163	0.000220798
PTCRA		-0.031	0.483567704
PRAG1	SGK223	-0.116	0.008759818
FLT4	VEGFR3	-0.052	0.242590542
MSI1		0.047	0.285098576
MAPKBP1	JNKBP1	-0.102	0.021858665
PTGDS	PDS	-0.175	6.78626E-05
TEDC2	C16orf59	0.222	0.000000426
ZFYVE21		-0.146	0.000938951
ZNF414		-0.141	0.001363306
BLCAP	BC10	-0.244	2.44E-08
NUMB	C14orf41	-0.152	0.000550091
RBM15	OTT	-0.015	0.733929399
AGER	RAGE	-0.331	1.79E-14
RAMP3		-0.190	1.57998E-05
RAB3IL1		0.335	7.12E-15
R3HDM2	KIAA1002	-0.175	6.84573E-05
RTN4RL1	NGRH2	-0.248	1.47E-08
ATXN1	ATX1	0.136	0.002080556
BMP7	OP1	-0.077	0.081236383
KCTD10	ULR061	0.236	7.27E-08
AAK1	KIAA1048	-0.010	0.81353985
AKT1	PKB	-0.114	0.010206626
ALDH3B1	ALDH7	-0.346	8.62E-16
MLLT11	AF1Q	0.318	2E-13
CELF5	BRUNOL5	0.015	0.734834814
HSPD1	HSP60	0.264	1.43E-09
ATXN1L	BOAT	-0.116	0.008847629
CCN3	IGFBP9	0.120	0.006854079
C5orf60		-0.019	0.664567359
CFD	DF	-0.231	0.000000127
SLC25A10	DIC	0.081	0.067481275
ATP2A1		-0.047	0.292601245
ATP2A2	ATP2B	0.102	0.021825679
ATP2A3		-0.160	0.000296614
RPS19BP1	AROS	-0.043	0.328386082
ARRB1	ARR1	-0.223	0.000000355
ARRB2	ARB2	-0.071	0.108744152
ARRDC1		-0.304	2.2E-12
ARRDC3	KIAA1376	-0.015	0.728898707
CHRNG	ACHRG	0.057	0.199297639
ATOH1	ATH1	-0.084	0.05784042
TNK2	ACK1	-0.220	0.000000494
BMP2	BMP2A	-0.174	8.01915E-05
BLOC1S2	BLOS2	0.024	0.583378701
CDH6		0.002	0.961634574
ADAMTSL3	KIAA1233	-0.210	1.80277E-06
EPN1		-0.039	0.384197711
IL17A	CTLA8	-0.005	0.911520587
HOXB9	HOX2E	0.114	0.010085989
HOXC8	HOX3A	0.133	0.002634856

IFT172	KIAA1179	-0.348	5.71E-16
HOXD3	HOX1D	0.170	0.000116226
IL2RA		0.276	2.45E-10
IL2RG		-0.018	0.68062472
ICAM4	LW	-0.086	0.051773362
IL6ST		-0.036	0.41253732
IFT74	CCDC2	0.102	0.020889727
IL6	IFNB2	0.204	0.000003445
NFKBIA	IKBA	0.028	0.529792326
HOXA1	HOX1F	0.311	6.4E-13
CST2		0.033	0.451897549
ELOB	TCEB2	-0.070	0.114542769
HCK		0.142	0.001253335
NAB2	MADER	-0.009	0.836114458
MYC	BHLHE39	0.288	3.6E-11
NBEA	BCL8B	-0.107	0.015249253
MYPOP	P42POP	-0.010	0.818447883
MYT1L	KIAA1106	0.052	0.239307719
ITCH		0.234	8.98E-08
H2BC12L	H2BFS	NA	NA
H2BC3	H2BFF	0.045	0.308508532
HDAC10		-0.253	6.53E-09
ONECUT1	HNF6	0.124	0.004984148
IKZF1	IK1	0.022	0.623993137
KRTAP10-5	KAP10.5	0.044	0.324232233
LIN7A	MALS1	0.102	0.021727226
CD46	MCP	-0.085	0.054273151
MIB2	SKD	-0.281	9.87E-11
H3-3A	H3.3A	-0.082	0.062802165
INS		-0.035	0.436547219
KAT2A	GCN5	NA	NA
KRTAP5-11	KAP5.11	-0.058	0.187462947
KRIT1	CCM1	-0.027	0.537378691
NEUROD4	ATH3	0.046	0.302852222
MAGEA1	MAGE1	0.077	0.084022533
GLRX3	PICOT	0.217	0.000000722
GSX2	GSH2	0.020	0.649369323
WWC1	KIAA0869	-0.281	9.98E-11
KIT	SCFR	-0.148	0.000778153
HEYL	BHLHB33	0.007	0.880291222
HEY1	BHLHB31	-0.059	0.186409737
HNF1B	TCF2	-0.236	7.03E-08
MAMLD1	CG1	0.030	0.499706404
MATN3		0.111	0.012299435
MIB1	DIP1	0.158	0.00033236
MTA1		0.004	0.934100842
CHIC2	BTL	0.143	0.001160612
FCER2	CD23A	-0.113	0.010734278
GABRD		0.142	0.001324747
GALNT11		-0.081	0.067614978
GMDS		-0.075	0.091227964

KRTAP5-3	KAP5-9	0.035	0.427083279
HES5	BHLHB38	-0.098	0.026737456
MAML3	KIAA1816	-0.281	1.12E-10
LCE2C	LEP11	-0.046	0.29531995
LCE1A	LEP1	0.019	0.671159185
CPLANE1	C5orf42	-0.019	0.670892083
CUL3	KIAA0617	-0.020	0.650745099
FADS2		0.186	2.33189E-05
KLHL38	C8orfK36	0.058	0.193451994
ACY3	ASPA2	-0.074	0.093393119
APH1B	PSFL	-0.211	1.51413E-06
BCAM	LU	-0.221	0.00000044
CERK	KIAA1646	-0.074	0.095076313
DOCK2	KIAA0209	0.055	0.211272933
DBH		-0.139	0.001604336
DTX3L	BBAP	0.146	0.000975011
DTX3	RNF154	-0.035	0.428862692
DTX4	KIAA0937	-0.148	0.000823349
YWHAZ		0.259	3.02E-09
HSD3B7		0.162	0.000249251
APP	A4	0.100	0.024238545
AQP5		-0.265	1.16E-09
CNNM3	ACDP3	-0.321	1.12E-13
CIR1	CIR	-0.111	0.012061497
CTNNBIP1	ICAT	-0.216	0.000000847
CARHSP1		0.126	0.004233339
CHSY1	CHSY	0.277	2.05E-10
CLDN2	PSEC0059	-0.195	9.46205E-06
CLEC18A	MRLP2	0.034	0.448385322
COL8A1	C3orf7	0.241	3.67E-08
C11orf87		0.240	3.98E-08
DLK2	EGFL9	0.102	0.021572692
GFOD1	C6orf114	0.085	0.055355818
GNE	GLCNE	0.026	0.564309582
IFI30	GILT	0.116	0.008899891
ALDH16A1		-0.249	1.28E-08
ATM		-0.104	0.019296569
BEND6	C6orf65	0.465	9.65E-29
BMP4	BMP2B	0.022	0.621287438
PRKAA2	AMPK	0.050	0.260028454
GOT1		0.011	0.798903496
ALX4	KIAA1788	0.012	0.791950466
ADAM12	MLTN	0.535	4.2E-39
AGO3	EIF2C3	0.148	0.000773664
DMRT3	DMRTA3	0.132	0.002852223
FOXC2	FKHL14	0.112	0.011200421
ADAM10	KUZ	0.148	0.000815353
AGO2	EIF2C2	0.163	0.000216667
CUL5	VACM1	0.004	0.925674811
NEDD9	CASL	-0.171	0.000100561
CTSG		-0.146	0.000975416

CREBBP	CBP	-0.084	0.057155558
DLL3		0.203	3.87293E-06
DNAL4		-0.122	0.005794262
FOXA1	HNF3A	0.042	0.348160189
BCL6	BCL5	-0.164	0.000191931
ACTA2	ACTSA	0.290	2.43E-11
B4GALT1	GGTB2	0.284	6.58E-11
ADAM17	CSVP	0.267	8.47E-10
ASB1	KIAA1146	-0.049	0.273702366
ASB2		0.014	0.75627732
ADAMTSL4	TSRC1	0.009	0.833280683
CRCT1	C1orf42	-0.072	0.104541005
AQP1	CHIP28	-0.140	0.001578437
ANXA4	ANX4	-0.220	0.000000502
EMC7	C11orf3	-0.035	0.428108491
CRACR2A	EFCAB4B	0.029	0.512401718
FTHL17		0.061	0.166309176
DRD5	DRD1B	0.074	0.096133619
AGO4	EIF2C4	-0.282	9.34E-11
CRY2	KIAA0658	-0.386	1.47E-19
CFAP58	C10orf80	-0.207	2.31393E-06
CCND1	BCL1	0.055	0.217694316
CYSRT1	C9orf169	-0.159	0.000314908
CREB5	CREBPA	0.138	0.00178916

Table S10: Correlation of mRNA levels between ALDH1B1 and NF-κB pathway-related molecules

Gene name	Secondary gene name	Rho	P-value
TNFRSF11A	RANK	0.090	0.041165105
UBA52	UBCEP2	0.093	0.035953009
RPS27A	UBA80	0.077	0.082188291
UBC		0.063	0.157792556
RELA	NFKB3	0.161	0.000259911
UBB		-0.139	0.001591687
CHUK	IKKA	0.154	0.000490011
PSMD11		0.271	4.68E-10
PSMB9	LMP2	0.157	0.000370623
PSMC4	MIP224	0.115	0.009467971
SEM1	C7orf76	0.149	0.000718446
PSMA7	HSPC	0.187	2.16913E-05
PSMA6	PROS27	0.280	1.26E-10
PSMC5	SUG1	0.007	0.880719704
PSMB6	LMPY	0.012	0.794233119
PSMB4	PROS26	0.082	0.063307255
MAP3K14	NIK	-0.068	0.126252828
PSMA5		0.126	0.00452948
NFKB1		-0.016	0.718227668
PSMD14	POH1	0.216	0.000000842

PSMB8	LMP7	0.074	0.095308026
PSMA3	HC8	0.128	0.003732448
PSMD4	MCB1	0.027	0.536171444
PSMB1	PSC5	0.125	0.004842385
PSMF1		0.033	0.461859631
PSMC3	TBP1	0.104	0.01911844
PDPK1	PDK1	-0.188	1.96494E-05
PDCD2L		0.217	0.000000716
TNFSF12	APO3L	-0.099	0.0248079
TAB1	MAP3K7IP1	-0.175	0.000069863
TAB2	KIAA0733	0.102	0.021210973
TAB3	MAP3K7IP3	-0.015	0.736380653
TRB		NA	NA
TRIM4	RNF87	-0.060	0.172745224
TNFSF14	HVEML	-0.155	0.000441338
SKP1	EMC19	-0.006	0.899141967
TRIM25	EFP	-0.053	0.233912758
S100A12		0.019	0.66134393
TRBC1		NA	NA
TRBC2	TCRBC2	NA	NA
TNFRSF13C	BAFFR	-0.012	0.795295903
IGLV1-40		NA	NA
IGLV1-44		NA	NA
IGLV1-47		NA	NA
IGLV1-51		NA	NA
IGLV2-8		NA	NA
IGLV2-11		NA	NA
MARCHF2		0.063	0.152410723
IGLV2-14		NA	NA
IGLV2-23		NA	NA
IGLV3-1		NA	NA
IGLV3-19		NA	NA
IGLV3-21		NA	NA
IGLV3-25		NA	NA
IGLV3-27		NA	NA
IGLV6-57		NA	NA
IGLV7-43		NA	NA
MAP3K1	MAPKKK1	-0.088	0.047676272
MAVS	IPS1	-0.190	1.59924E-05
MALT1	MLT	0.102	0.021499269
MAP3K7	TAK1	0.220	0.000000524
BCL10	CIPER	0.200	5.03956E-06
HMGB1	HMG1	0.031	0.487428183
IGKV1-16		NA	NA
IGLC1		NA	NA
IGLC7		NA	NA
IGKV2-29		NA	NA
IGKV2-30		NA	NA
IGKV2D-28		NA	NA
LYN	JTK8	0.273	3.9E-10
IGKV1-12		NA	NA

NKRF	ITBA4	0.262	1.93E-09
FCER1A	FCE1A	-0.121	0.006204856
IGKC		NA	NA
IFIH1	MDA5	0.143	0.001187761
IGKV1D-12		NA	NA
IGKV5-2		NA	NA
IGKV1-5		NA	NA
CASP10	MCH4	0.008	0.850154736
IGKV3D-20		NA	NA
NKIRAS1	KBRAS1	-0.063	0.156556789
CARD11	CARMA1	NA	NA
C1QTNF4	CTRP4	0.098	0.026886618
CD40LG	CD40L	-0.157	0.000364829
FBXW11	BTRCP2	-0.038	0.38928697
DEPDC1	DEPDC1A	0.352	2.47E-16
DDX58		0.218	0.000000628
IGKV4-1		NA	NA
BST2		0.106	0.016810413
IGKV3-20		NA	NA
IGLC2		NA	NA
HEMGN	EDAG	-0.056	0.203424245
IGKV1-39		NA	NA
IGKV3-15		NA	NA
PRKCQ	PRKCT	-0.166	0.000171141
IGKV2-28		NA	NA
IGLC3		NA	NA
RASGRP1	RASGRP	0.078	0.078272898
RASGRP4		-0.012	0.794941313
IGKV1-17		NA	NA
FADD	MORT1	0.189	1.81279E-05
CUL1		0.133	0.002715852
MS4A2	APY	-0.116	0.00884707
IGKV3-11		NA	NA
NFKB2	LYT10	-0.072	0.102354504
RELB		0.130	0.00327909
RNF135	L13	-0.060	0.177498957
CASP8	MCH5	0.036	0.419873795
IGKV1D-33		NA	NA
NISCH	IRAS	-0.256	4.74E-09
RIPK1	RIP	-0.064	0.151820809
SGO2	SGOL2	0.384	2.56E-19
PSMB2		0.150	0.000668692
PSMD8		0.101	0.022208619
PSMB11		-0.032	0.464769842
PSMD9		0.183	3.24187E-05
SETD6		-0.169	0.000125438
PSMB3		0.192	1.21706E-05
TNFSF11	OPGL	0.108	0.015092102
TNFAIP8		0.011	0.79619776
PSMB5	LMPX	0.173	8.36453E-05
TRAV6	TCRAV5S1	NA	NA

PSMD7	MOV34L	0.132	0.002816502
PSMB7	Z	0.112	0.011489039
PSMB10	LMP10	-0.056	0.202899721
PSME1	IFI5111	0.090	0.041101063
LTB	TNFC	-0.088	0.046514798
CD40	TNFRSF5	0.052	0.241851279
NGFR	TNFRSF16	-0.152	0.000549277
PSMD1		0.159	0.000306201
TNF	TNFA	0.087	0.050786821
SQSTM1	ORCA	-0.075	0.091060607
S100B		0.042	0.348699388
PSMD10		0.176	6.42774E-05
PSME3		0.309	9.47E-13
PSMC1		0.113	0.010477901
NLRP3	C1orf7	0.078	0.079649748
PSMD12		0.304	2.14E-12
TRAV29DV5		NA	NA
IGKV1D-39		NA	NA
TNFRSF1B	TNFBR	-0.078	0.077035101
TNFRSF12A	FN14	0.124	0.005002374
SAA1		0.150	0.000670792
IGKV1D-16		NA	NA
TANK	ITRAF	-0.026	0.557990213
TRAF3	CAP1	0.087	0.049376289
PSMA8	PSMA7L	0.073	0.098715423
TRBV7-9		NA	NA
PSMD3		0.210	1.65074E-06
PSMD2	TRAP2	0.264	1.47E-09
PSMA2	HC3	0.269	6.62E-10
TRAV8-4		NA	NA
UBE2D2	PUBC1	0.142	0.001289671
PSMC2	MSS1	0.267	9.01E-10
PSMA1	HC2	0.114	0.01017893
PSMC6	SUG2	0.157	0.000363285
UBE2V1	CROC1	0.105	0.017679622
LTBR	D12S370	-0.001	0.978842033
TRAV19		NA	NA
UBA3	UBE1C	0.159	0.000301476
TRAV39		NA	NA
TRAV23DV6		NA	NA
PSMD5	KIAA0072	0.140	0.001524555
PSME2		0.162	0.000230342
PSMD6	KIAA0107	0.171	0.000104287
TRAV9-2		NA	NA
TNFSF13B	BAFF	0.158	0.000344312
TRAF6	RNF85	-0.031	0.486656871
TRAV22		NA	NA
TRAV12-2		NA	NA
UBE2N	BLU	0.235	7.74E-08
PSME4	KIAA0077	0.174	7.64579E-05
PYDC1	ASC2	0.044	0.317224911

LTA	TNFB	0.034	0.438076622
TRAC	TCRA	NA	NA
PSMA4	HC9	0.029	0.507181977
PSMD13		0.135	0.002251388
ZNF224	BMZF2	-0.254	6.23E-09
TRAF2	TRAP3	-0.157	0.00035847
TRBV12-3	TCRBV12S3	NA	NA
TRAV21		NA	NA
TRBV6-5		NA	NA
CDC34	UBCH3	0.225	0.000000295
UBE2M	UBC12	0.099	0.025007891
TBK1	NAK	0.100	0.02342874
UBE2D1	SFT	0.286	4.66E-11
RAF1	RAF	-0.038	0.387782912
AGER	RAGE	-0.331	1.79E-14
BIRC3	API2	NA	NA
PYCARD	ASC	-0.062	0.163971913
IGHV1-2		NA	NA
IGHV1-46		NA	NA
IGHV1-69		NA	NA
IGHV2-5		NA	NA
IGHV2-70		NA	NA
IGHV3-7		NA	NA
IGHV3-9		NA	NA
IGHV3-11		NA	NA
IGHV3-13		NA	NA
IGHV3-23		NA	NA
IGHV3-30		NA	NA
IGHV3-33		NA	NA
IGHV3-48		NA	NA
IGHV3-53		NA	NA
IGHV4-34		NA	NA
IGHV4-39		NA	NA
IGHV4-59		NA	NA
NFKBIA	IKBA	0.028	0.529792326
NFKBIB	IKBB	-0.168	0.000134219
IKBKB	IKKB	-0.128	0.003769382
IGKV2D-30		NA	NA
IGKV2D-40		NA	NA
IKBKG	FIP3	-0.053	0.229618707
NGF	NGFB	0.134	0.002451199
RASGRP2	CDC25L	-0.150	0.000675506
BTRC	BTRCP	-0.097	0.029221654
IGHE		NA	NA
IRAK1	IRAK	0.235	0.000000079
FCER1G		0.215	0.000000911
IGKV1-33		NA	NA
IGLC6		NA	NA
NKIRAS2	KBRAS2	0.183	3.20079E-05
EIF2AK2	PKR	0.232	0.00000012
APP	A4	0.100	0.024238545

BIRC2	API1	0.163	0.000220206
EHMT1	EUHMTASE1	-0.081	0.068717989

Table S11: Correlation of mRNA levels between ALDH1B1 and gluconeogenesis pathway-related molecules

Gene name	Secondary gene name	Rho	P-value
FBP1	FBP	-0.111	0.011907811
CRY1	PHLL1	0.107	0.015927111
SLC37A4	G6PT	-0.084	0.058814884
G6PC1	G6PC	-0.028	0.522281264
TPI1	TPI	0.284	6.02E-11
G6PC2	IGRP	-0.192	1.27725E-05
G6PC3	UGRP	-0.089	0.045549366
PCK2	PEPCK2	0.042	0.338818604
GPI		0.229	0.000000174
FBP2		-0.149	0.000723459
PC		-0.115	0.009372194
DDB1	XAP1	0.089	0.043999772
SLC39A14	KIAA0062	0.311	7.03E-13
SIK1	SIK	-0.207	2.38561E-06
SIRT7	SIR2L7	-0.138	0.001802395
SDS	SDH	0.089	0.045209892
PPARGC1A	LEM6	-0.237	6.23E-08
USP7	HAUSP	-0.021	0.638292245
NR1D1	EAR1	0.148	0.000801127
PGAM2	PGAMM	-0.043	0.334250159
SLC25A11	SLC20A4	-0.101	0.022951739
KLF10	TIEG	0.176	6.20675E-05
SLC25A13	ARALAR2	0.256	4.72E-09
CCAR2	DBC1	-0.155	0.00042601
NNMT		0.041	0.360182058
SLC25A1	SLC20A3	0.003	0.952149956
PTPN2	PTPT	0.089	0.04355703
ZNF692	AREBP	-0.238	5.37E-08
SLC25A10	DIC	0.081	0.067481275
CRTC2	TORC2	-0.044	0.318042847
ENO2		0.162	0.00023872
GCG		0.153	0.000538218
CLK2		-0.130	0.003271834
GPT	AAT1	-0.267	9.39E-10
NR3C1	GRL	-0.130	0.003176108
ALDOA	ALDA	0.160	0.000287929
SLC25A12	ARALAR1	0.112	0.011401161
C1QTNF3	CTRP3	0.198	6.46946E-06
PER2	KIAA0347	-0.372	3.23E-18
PGM1		0.131	0.003101534
PGAM1	PGAMA	0.382	3.6E-19
SOGA1	C20orf117	-0.035	0.424078224
SELENOS	SELS	-0.051	0.254808149

FFAR3	GPR41	0.089	0.044255919
MDH1	MDHA	0.080	0.069856885
MDH2		0.212	1.28252E-06
PFKFB1	F6PK	-0.110	0.013140124
GCGR		-0.009	0.835278467
ENO3		-0.157	0.000363757
FOXK1	MNF	0.089	0.043611904
GCK		0.105	0.017408431
ATF4	CREB2	-0.015	0.740952388
DGKQ	DAGK4	-0.208	2.13125E-06
AQP7	AQP7L	-0.240	4.24E-08
GAPDHS	GAPD2	-0.053	0.235970965
GAPDH	GAPD	0.297	7.78E-12
DGAT2	HMFN1045	-0.019	0.676731747
KAT2B	PCAF	-0.166	0.00016701
JAZF1	TIP27	-0.015	0.732460862
FOXK2	ILF	0.146	0.000970992
PPP4R3B	KIAA1387	0.090	0.042121872
SERPINA12		0.056	0.204244082
RMND5A		-0.110	0.012738069
SLC35B4	YEA4	0.258	3.53E-09
PGP		0.063	0.156677593
WDR5	BIG3	0.137	0.00194195
UBR5	EDD	0.115	0.009571083
RANBP2	NUP358	-0.017	0.700902546
ARPP19		0.048	0.280937987
FAM3C	ILEI	0.299	5.43E-12
GNMT		-0.294	1.17E-11
GPD1		-0.144	0.00112327
KAT2A	GCN5	NA	NA
MAEA	EMP	-0.050	0.260861322
GOT2	KYAT4	0.065	0.141592362
ATF3		0.020	0.660311632
ACADM		-0.057	0.198124068
PRKAA2	AMPK	0.050	0.260028454
GOT1		0.011	0.798903496
ALDOB	ALDB	-0.154	0.000467907
ALDOC	ALDC	0.209	1.96646E-06
CRY2	KIAA0658	-0.386	1.47E-19
SLC37A2		0.055	0.216922655
PGK2	PGKB	0.002	0.972617652
NLN	AGTBP	0.390	5.13E-20
SLC37A1	G3PP	-0.189	1.75504E-05
RMND5B	UNQ2508/PRO5996	-0.275	2.57E-10
FAM3A		-0.086	0.051698427
ERFE	C1QTNF15	NA	NA
SDHAF3	ACN9	0.210	1.68089E-06

Table S12: Correlation of mRNA levels between ALDH1B1 and glycolysis pathway-related molecules

Gene name	Secondary gene name	Rho	P-value
TIGAR	C12orf5	0.228	0.000000205
PFKM	PFKX	0.101	0.021960722
PKM	OIP3	0.174	7.48452E-05
ENO1	ENO1L1	0.251	9.16E-09
PFKL		-0.078	0.079509583
PFKP	PFKF	0.276	2.16E-10
PGK1	PGKA	0.341	2.33E-15
HK3		0.119	0.006976358
GPI		0.229	0.000000174
HK1		-0.021	0.636158646
FOXK1	MNF	0.089	0.043611904
GCK		0.105	0.017408431
GAPDH	GAPD	0.297	7.78E-12
ENO2		0.162	0.00023872
HK2		0.025	0.568038282
ALDOA	ALDA	0.160	0.000287929
TPI1	TPI	0.284	6.02E-11
ENO3		-0.157	0.000363757
PKLR	PK1	-0.025	0.57047491
FOXK2	ILF	0.146	0.000970992
ALDOC	ALDC	0.209	1.96646E-06
ENTPD5	CD39L4	0.019	0.670158123
GAPDHS	GAPD2	-0.053	0.235970965
PFKFB3		0.124	0.005070148
ALDOB	ALDB	-0.154	0.000467907
ADPGK	PSEC0260	-0.045	0.305582442
PGK2	PGKB	0.002	0.972617652
PGM2L1	BM32A	0.301	3.61E-12
PGAM1	PGAMA	0.382	3.6E-19
PGAM2	PGAMM	-0.043	0.334250159
PGAM4	PGAM3	0.365	1.47E-17
ATP6V1E1	ATP6E	-0.010	0.830158233
OGT		-0.115	0.009399197
TRAP1	HSP75	0.096	0.029952906
TPP2		-0.062	0.164424896
HIF1A	BHLHE78	0.313	4.45E-13
ISCU	NIFUN	-0.208	2.23061E-06
LIN28A	CSDD1	0.018	0.691262199
SLC16A1	MCT1	0.311	7.26E-13
PRKACB		-0.002	0.963106942
DHTKD1	KIAA1630	-0.132	0.002748198
PFKFB2		-0.191	1.45205E-05
PFKFB1	F6PK	-0.110	0.013140124
EIF6	EIF3A	0.079	0.07507229
PPBP	CTAP3	0.103	0.019448359
MACIR	C5orf30	0.030	0.505488461

MLXIP	BHLHE36	0.042	0.346291785
MR1		-0.069	0.120712877
GNPDA2	GNP2	0.118	0.007790387
BSG	UNQ6505/PRO21383	0.005	0.905155083
PFKFB4		0.328	2.86E-14
PRKACG		0.020	0.647219913
KLF10	TIEG	0.176	6.20675E-05
G6PD		0.018	0.690218381
EGLN3		0.256	4.24E-09
SLC2A6	GLUT6	0.095	0.031090945
SLC2A1	GLUT1	0.259	2.82E-09
NECAB3	APBA2BP	-0.255	5.48E-09
GIT1		0.191	1.47152E-05
ENO4	C10orf134	NA	NA
EP300	P300	-0.039	0.383230831
CBFA2T3	MTG16	-0.148	0.000816445
OGDH		-0.064	0.146907128
MLX	BHLHD13	0.043	0.32883269
OGDHL	KIAA1290	0.076	0.086250321
ADSS1	ADSSL1	-0.118	0.007833329
PDK2	PDHK2	-0.289	2.96E-11
BPGM		0.194	9.9888E-06
SRC	SRC1	-0.034	0.446969041
PRXL2C	AAED1	0.300	4.34E-12
SLC25A51	MCART1	0.299	5.11E-12
PGP		0.063	0.156677593
PPP2CA		0.148	0.000807226
PPP2CB		-0.090	0.041742859
NXNL1	TXNL6	-0.089	0.044413341
ZBTB7A	FBI1	-0.146	0.000915514
TKTL1	TKR	-0.041	0.36110745
ACMSD		-0.046	0.294680092
KEAP1	INRF2	-0.027	0.543182409
HKDC1		-0.126	0.004369809
INS		-0.035	0.436547219
PRKACA	PKACA	0.104	0.018498861
GNPDA1	GNPI	0.213	1.28046E-06
GCG		0.153	0.000538218
ECD		0.068	0.126300486
PPP2R5D		0.165	0.000180622
PPP2R1A		-0.068	0.122842122
PRKAA1	AMPK1	-0.109	0.01363993
PRKAA2	AMPK	0.050	0.260028454
H6PD	GDH	-0.181	4.06793E-05
PPP2R1B		0.045	0.314397105

Table S13: Correlation of mRNA levels between ALDH1B1 and cell surface markers and other ALDH isoforms

Gene name	Secondary gene name	Rho	P-value
CD166	ALCAM	-0,095	0,032919387
CD90	THY1	0,435	1,09412E-24
CD87	PLAUR	0,290	3,39056E-11
CD44		0,077	0,085231368
CD133	PROM1	-0,0065	0,885015164
ALDH1A1		-0,204	4,08794E-06
ALDH1A2		0,079	0,076415089
ALDH1A3		0,244	2,99103E-08
ALDH1L1		-0,020	0,6532997
ALDH1L2		0,361	5,68554E-17
ALDH2		-0,309	1,25882E-12
ALDH3A1		-0,286	6,59862E-11
ALDH3A2		-0,237	7,47876E-08
ALDH3B1		-0,345	1,55076E-15
ALDH3B2		-0,001	0,980524589
ALDH4A1		-0,231	1,48508E-07
ALDH5A1		-0,330	2,75697E-14
ALDH6A1		-0,128	0,004053923
ALDH7A1		-0,079	0,078196156
ALDH8A1		0,006	0,88917905
ALDH9A1		-0,177	6,69845E-05
ALDH16A1		-0,242	3,66551E-08
ALDH18A1		0,137	0,002088092
